# Chloroacetamide‐Modified Nucleotide and RNA for Bioconjugations and Cross‐Linking with RNA‐Binding Proteins

**DOI:** 10.1002/anie.202213764

**Published:** 2023-01-12

**Authors:** Mária Brunderová, Matouš Krömer, Marta Vlková, Michal Hocek

**Affiliations:** ^1^ Institute of Organic Chemistry and Biochemistry of the Czech Academy of Sciences Flemingovo nám. 2 16000 Prague 6 Czech Republic; ^2^ Department of Organic Chemistry Faculty of Science Charles University Hlavova 8 12843 Prague 2 Czech Republic

**Keywords:** Bioconjugations, Cross-Linking, Modified RNA, Proteins, RNA Polymerases

## Abstract

Reactive RNA probes are useful for studying and identifying RNA‐binding proteins. To that end, we designed and synthesized chloroacetamide‐linked 7‐deaza‐ATP which was a good substrate for T7 RNA polymerase in *in vitro* transcription assay to synthesize reactive RNA probes bearing one or several reactive modifications. Modified RNA probes reacted with thiol‐containing molecules as well as with cysteine‐ or histidine‐containing peptides to form stable covalent products. They also reacted selectively with RNA‐binding proteins to form cross‐linked conjugates in high conversions thanks to proximity effect. Our modified nucleotide and RNA probes are promising tools for applications in RNA (bio)conjugations or RNA proteomics.

## Graphical abstract

A modified nucleotide for construction of reactive RNA probes that form covalent cross‐linked conjugates with Cys‐ or His‐containing peptides and proteins is reported. The nucleotide and probes are promising tools for applications in RNA (bio)conjugations or RNA proteomics.

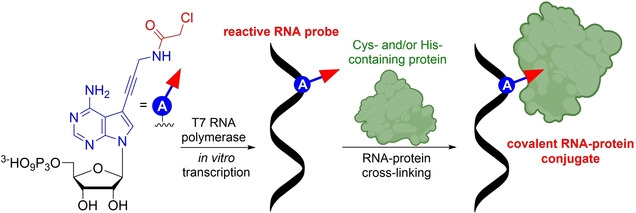

RNA‐protein interactions (RPIs) are of paramount importance in cell biology and are subject of intensive current research.^[1]^ Covalent cross‐linking of RNA with interacting proteins is the most efficient way to capture and identify RNA‐binding proteins (RBPs),^[2]^ in particular those that bind too weakly to survive non‐covalent pull‐down protocols. The most frequently used methods for formation of covalent conjugates are photochemical cross‐linking with either natural RNA,^[3]^ RNA containing photo‐activable groups^[4]^ or usage of external chemical cross‐linking agents, i.e. formaldehyde,[^3c^, ^5^] 1‐ethyl‐3‐dimethyl aminopropylcarbodiimide (EDC)^[6]^ or dithiothreitol (DTT).^[7]^ These approaches have inherent limitations, such as low efficiency and poor yields of product, non‐specific binding, and/or application of damaging UV light. In case of chemical cross‐linking, the probing RPI in living cells is hindered due to toxic nature of these agents and non‐specific cross‐linking of protein‐protein interactions can distort interpretation of obtained results.

Some of these drawbacks could be overcome with RNA probes bearing amino acid‐specific reactive groups. In DNA, several nucleotides bearing reactive modifications have been described that can be enzymatically incorporated to DNA probes, e.g. vinylsulfonamide reacting with cysteines (Cys),^[8]^ chloroacetamide reacting with Cys or histidines (His),^[9]^ aldehydes^[10]^ or squaramate^[11]^ for lysines (Lys), and glyoxal^[12]^ or 1,3‐diketones^[13]^ for arginines (Arg). Conversely, in RNA only scarce examples of the use of 5‐fluoro‐ or 5‐azapyrimidine nucleotides for metabolic labelling and cross‐linking with Cys‐containing methyltransferases have been reported.^[14]^ On the other hand, enzymatic synthesis of modified RNA with T7 RNA polymerase (T7 RNAP) is a well‐established method whereby modified ribonucleotides can replace natural counterparts at all sites[^15^, ^16^, ^17^] or at site‐specific positions in RNA.^[18]^ Several examples of reactive modifications for posttranscriptional labelling using bioorthogonal reactions[^19^, ^20^] (however unsuitable for cross‐linking with native proteins) or methyltransferase‐mediated modifications of RNA caps have been published.^[21]^ Also, syntheses of RNA‐peptide^[22]^ and RNA‐small molecule^[23]^ conjugates are of interest for the use in fluorescent labelling, affinity enrichment or targeted delivery. Here we present development of Cys‐ (thiol‐) or His‐reactive RNA probes easily accessible through enzymatic synthesis by incorporation of the corresponding chloroacetamide (CA)‐modified ribonucleoside triphosphate (rNTP) with T7 RNAP.

We designed ribonucleoside triphosphate **rA^CA^TP** bearing CA group at position 7 of 7‐deazaadenine base tethered through aminopropagyl linker. Synthesis of **rA^CA^TP** was performed by single step Sonogashira cross‐coupling reaction of 7‐iodo‐7‐deazaadenosine‐5′‐*O*‐triphosphate (**rA^I^TP**) with *N*‐(propargyl)‐chloroacetamide (**1**) in analogy to previous work.^[9]^ The reaction proceeded in presence of Pd(OAc)_2_, CuI, TPPTS and DIPEA in a mixture of H_2_O/acetonitrile (2 : 1) (Figure 1A, Section 1.2 in Supporting Information) and after reversed‐phase HPLC purification the product was isolated in satisfactory 24 % yield and fully characterized by MS and NMR spectroscopy (Figures S1–S4 in Supporting Information).

**Figure 1:**
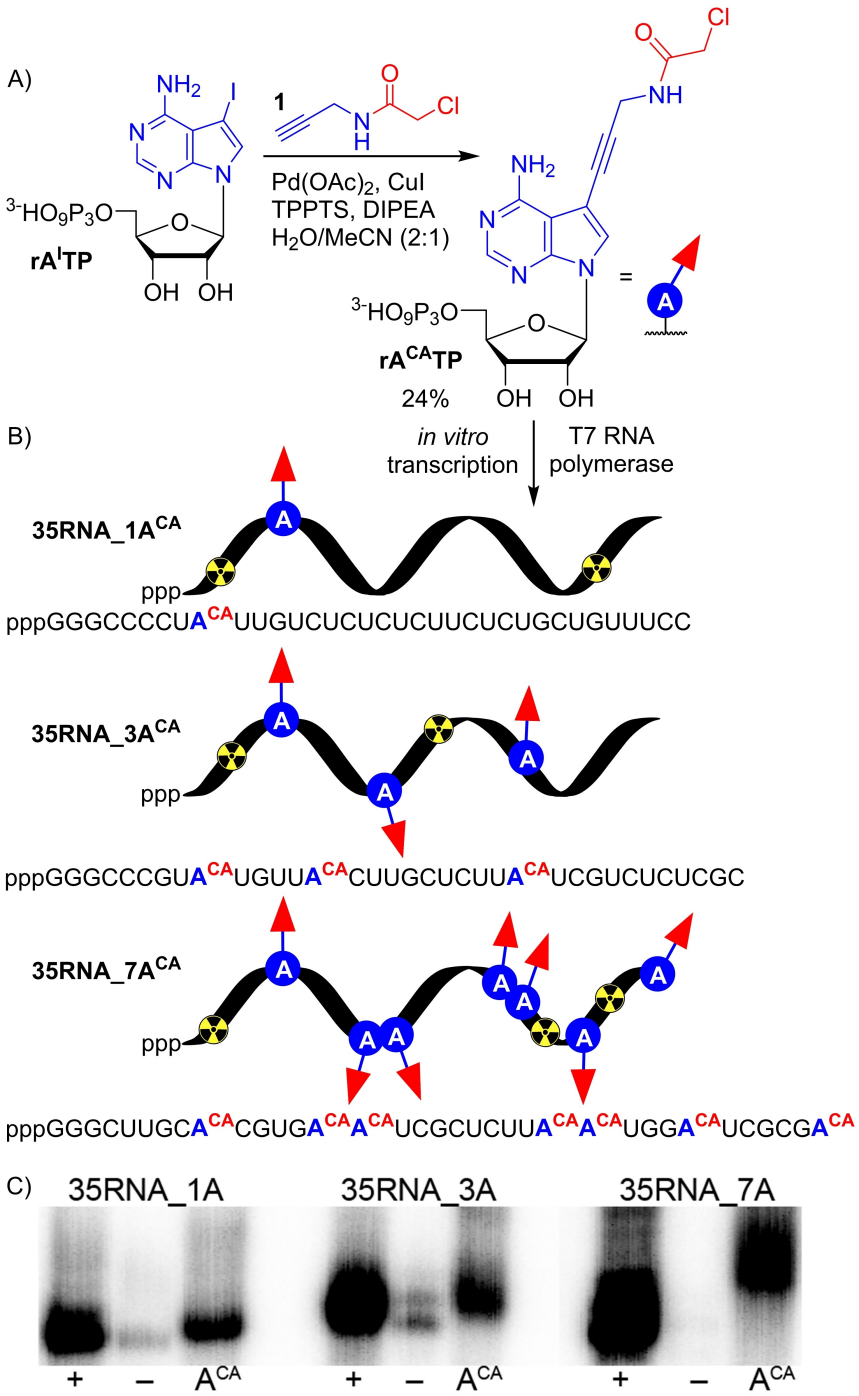
A) Chemical synthesis of **rA^CA^TP**. B) Enzymatic construction of (CA)‐modified RNA using T7 RNAP. C) Phosphor imaging of radioactive 12.5 % dPAGE analysis of transcription reactions performed with various DNA templates: **35DNA_1A**, **35DNA_3A** or **35DNA_7A**. (+) positive control, all natural rNTPs (rATP, rCTP, UTP, rGTP); (−) negative control, mixture of rCTP, UTP, rGTP and H_2_O; (A^CA^) mixture of **rA^CA^TP**, rCTP, UTP, rGTP. For uncropped gel scan see Figure S9 in Supporting Information.

The modified **rA^CA^TP** was subsequently tested as a putative substrate for T7 RNAP in transcription assays. The crucial step was to determine whether the reactive CA moiety cross‐links to T7 RNAP, since it contains 12 reduced cysteines.^[24]^ To this end, we performed control experiments (Figures S6, S7 in Supporting Information), that ruled out any significant cross‐linking of modified RNA to T7 RNAP and showed that the **rA^CA^TP** did not inhibit transcription (Section 2.6.4.1. in Supporting Information). Next, we designed three DNA templates with different sequences encoding for 35‐nucleotide (nt) RNA containing one (**35RNA_1A^CA^**), three (**35RNA_3A^CA^**) or seven (**35RNA_7A^CA^**) CA modifications (Figure 1B, Tables S2.2, S2.3 in Supporting Information). The **rA^CA^TP** was well tolerated by T7 RNAP in transcription reaction and full‐length RNA products were formed as elucidated by denaturing polyacrylamide gel electrophoresis (dPAGE, Figure 1C, uncropped gel scan Figure S9 in Supporting Information) and confirmed by MS‐MALDI‐TOF analysis (Figures S37–S39 in Supporting Information).

To test reactivity of the modified RNA probes, we performed model bioconjugation reactions with Cys‐ and His‐containing peptides and thiol‐linked biotin and fluorescein. To this end, we designed and prepared a short 20‐nt **20RNA_1A^CA^** containing one modification (Figures S8, S33, S35 in Supporting Information). To avoid usage of hazardous radioactive labelling, we tagged the short RNA with Cy5 fluorophore through ligation reaction^[25]^ using T4 RNA ligase and cytidine‐5′‐phosphate‐3′‐(Cy5‐aminohexyl)phosphate, pCp‐Cy5 (Figure 2A). The reaction proceeded smoothly to give the Cy5‐labelled 21‐nt long **21RNA_1A^CA^‐Cy5** in virtually full conversion as determined by dPAGE analysis (Figure S10 in Supporting Information). The product was also characterized by MS‐MALDI‐TOF analysis (Figure S41 in Supporting Information).

**Figure 2:**
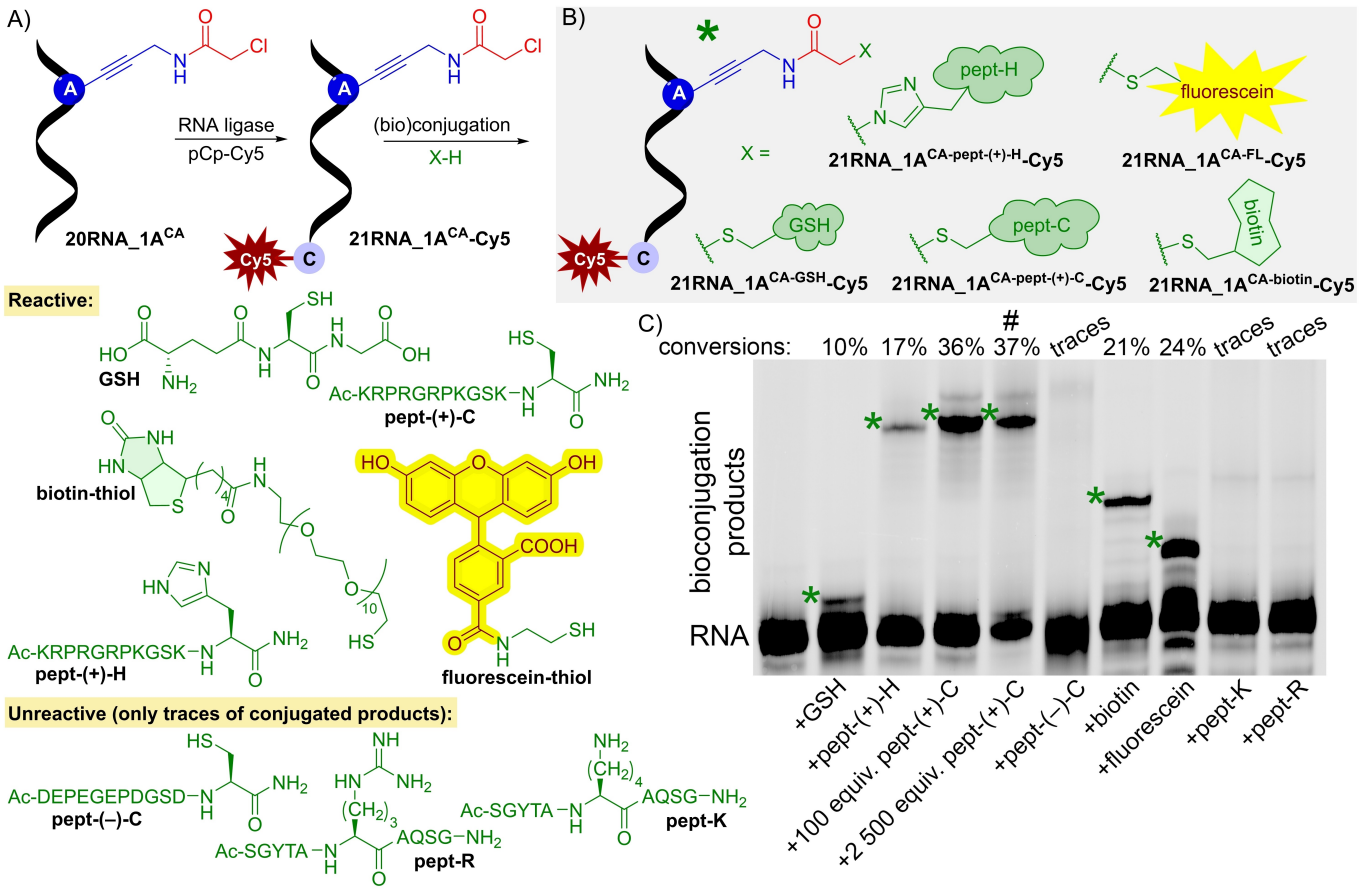
A) Fluorescent labelling of RNA via ligation reaction. B) Model bioconjugation reactions of modified RNA, **21RNA_1A^CA^‐Cy5** with various (bio)molecules. C) Cy5‐scan of 22.5 % dPAGE after bioconjugation. (RNA) modified RNA, standard; modified RNA in reaction with: 10 000 equiv of reduced glutathione (GSH); 10 000 equiv of pept‐(+)‐H; 100 equiv of pept‐(+)‐C; 2500 equiv of pept‐(+)‐C, (#) significant formation of side‐product affects calculated conversion, side‐product formed in 18 % yield, combined conversion (bioconjugated product and side‐product) in 55 % yield; 2 500 equiv of pept‐(−)‐C; 10 000 equiv of biotin‐thiol; 10 000 equiv of fluorescein‐thiol; 10 000 equiv of pept‐K and 10 000 equiv of pept‐R. For uncropped gel scan see Figure S12 in Supporting Information.

Model bioconjugation reactions were performed in presence of triethylammonium acetate buffer (TEAA) or in HEPES‐NaOH buffer at neutral pH in presence of 10 000 equiv excess of thiol‐linked (bio)molecules. Reactions with biotin‐ or fluorescein‐thiol proceeded with moderate conversions to give desired conjugation products **21RNA_1A^CA‐biotin^‐Cy5** (21 %), **21RNA_1A^CA‐FL^‐Cy5** (24 %), which were characterized by gel mobility shift assay (Figure 2C, uncropped gel scan Figure S12 in Supporting Information), MS‐MALDI‐TOF analysis (Figures S47, S48 in Supporting Information) and (the latter) by fluorescent measurements (Figure S15 in Supporting Information). In order to test reactivity of **21RNA_1A^CA^‐Cy5** with peptides (Figure 2B), we used reduced glutathione (GSH) and a set of positively or negatively charged peptide sequences containing one Cys, a positively charged peptide with one His, and two peptide sequences lacking Cys and His, but containing either Lys or Arg, as negative controls (Section 2.8.1 in Supporting Information). As we anticipated, due to strong affinity to negatively charged RNA and higher reactivity of Cys, cationic Cys‐containing peptide **pept‐(+)‐C** was the most reactive giving the conjugated product **21RNA_1A^CA‐pept‐(+)‐C^‐Cy5** in good 36 % yield with 100 equiv of the peptide. Remarkably, when the amount of **pept‐(+)‐C** was further increased, a lower‐mobile side‐product was observed on dPAGE (Figure 2C, uncropped gel scan Figure S12 in Supporting Information, Figure S13 in Supporting Information), which is probably a non‐covalent aggregate, since LC–MS analysis of purified mixture in this case showed only peaks corresponding to free RNA and RNA‐peptide conjugate (Figure S14 in Supporting Information). His‐containing cationic peptide **pept‐(+)‐H** was then less reactive giving **21RNA_1A^CA‐pept‐(+)‐H^‐Cy5** in 17 %. Also using large excess of GSH tripeptide gave the desired conjugate **21RNA_1A^CA‐GSH^‐Cy5** (10 %). All these bioconjugation products were fully characterized by dPAGE (Figure 2C, uncropped gel scan Figure S12 in Supporting Information), MS‐MALDI‐TOF (Figures S44–S46 in Supporting Information) and for **pept‐(+)‐C** also by LC‐ESI‐MS (Figures S51, S52 in Supporting Information). In case of negatively charged peptide with Cys and/or peptides not containing any Cys or His, reaction with modified RNA produced only traces of bioconjugation products **21RNA_1A^CA‐pept‐(−)‐C^‐Cy5**, **21RNA_1A^CA‐pept‐K^‐Cy5**, **21RNA_1A^CA‐pept‐R^‐Cy5** as determined by dPAGE analysis (Figure 2C, uncropped gel scan Figure S12 in Supporting Information).

Next, we advanced to the ultimate goal of this study, reacting the (CA)‐modified RNA with proteins. RNA‐protein cross‐linking was studied with three different RBPs with diverse biological functions, each of them containing several Cys and His amino acids (Section 4 in Supporting Information). We selected human argonaute 2 protein (hAgo2),^[26]^ human antigen R (HuR/ELAVL1)^[27]^ and HIV reverse transcriptase (HIV‐RT).^[28]^ First, we performed electrophoretic mobility shift assay (EMSA) to investigate whether the CA functionality does not interfere with binding of proteins to the modified RNA (Section 2.10 in Supporting Information). RNA‐protein binding studies were performed at physiological neutral pH, either with equimolar ratio (HuR) or with slight excess of 2 equiv (HIV‐RT, hAgo2) of proteins. Analysis of reactions by 5 % native‐PAGE showed in all cases a slower moving band compared to the corresponding free nucleic acid standard (Figures S16–S18 in Supporting Information) confirming presence of protein‐RNA complexes. Next, we sought to explore formation of covalent bond between RNA probe **21RNA_1A^CA^‐Cy5** and above‐mentioned proteins (Section 2.11 in Supporting Information). Contrary to studied model bioconjugation reactions, the cross‐linking reactions were performed with only 2 equiv of each of the proteins since we expected strong contribution of proximity effect. Cross‐linking was monitored by denaturing SDS‐PAGE that revealed lower mobility bands corresponding to formed covalent RNA‐protein adducts **21RNA_1A^CA‐HuR^‐Cy5** (30 %), **21RNA_1A^CA‐HIV‐RT^‐Cy5** (28 %) and **21RNA_1A^CA‐hAgo2^‐Cy5** (22 %) (Figure 3, SDS‐PAGE analysis, uncropped gel scan Figure S20 in Supporting Information). Simple kinetic studies of cross‐linking reaction in case of HIV‐RT (Figure S19 in Supporting Information) revealed presence of the product already within 30 minutes (3 % conversion). After 6 hours the conversion (24 %) was already exceeding established photocross‐linking methods.^[29]^ These results are comparable and competitive with the related proximity driven protein‐protein crosslinking methods employed in cell studies.^[30]^

**Figure 3:**
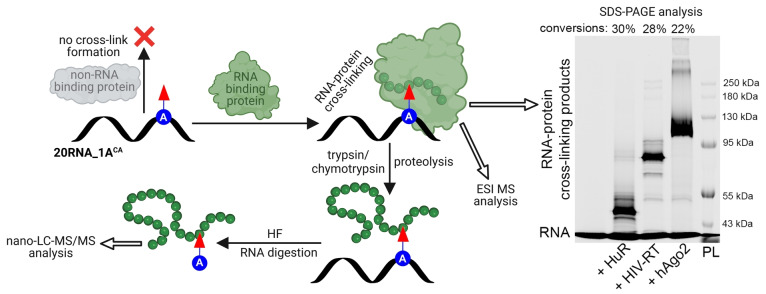
RNA cross‐linking with various proteins and intact ESI‐MS or nano‐LC–MS/MS analysis of cross‐linking products. Cy5 channel scan of 7 % denaturing SDS‐PAGE analysis of cross‐linking between modified RNA, **21RNA_1A^CA^‐Cy5** and RBPs (2 equiv of proteins). (RNA) modified RNA, standard; modified RNA in reaction with: (HuR) human antigen R protein; (HIV‐RT) HIV reverse transcriptase; (hAgo2) human argonaute 2 protein; (PL) pre‐stained protein ladder. For uncropped gel scan see Figure S20 in Supporting Information. For copies of spectra from nano‐LC–MS/MS analysis see Figures S71–S83 in Supporting Information. For copies of spectra from intact ESI MS analysis see Figures S61–S64 and Figures S67–S68.

To uncover relationship between number of modifications, cross‐linking efficiency and selectivity for RBPs, we prepared RNA probes with one (**36RNA_1A^CA^‐Cy5**), three (**36RNA_3A^CA^‐Cy5**) or seven (**36RNA_7A^CA^‐Cy5**) CA modifications (Figure S10 in Supporting Information) and compared them in cross‐linking reactions with HuR (Figure S23 in Supporting Information). BSA was used as off‐target control. Cross‐linking conversions increased with number of reactive groups, however for 7 modifications, the selectivity was already compromised (Figure S24 in Supporting Information). Hence, to balance specificity and conversions, density of modifications needs to be considered on case‐by‐case basis.

Cross‐linking was furthermore corroborated by western blot analysis (WB, Section 2.12 in Supporting Information). Shift of the proteins was observed only in case of modified **20RNA_1A^CA^**, which proves formation of covalent RNA‐protein conjugates **20RNA_1A^CA‐HuR^**, **20RNA_1A^CA‐HIV‐RT^**, **20RNA_1A^CA‐hAgo2^** through reactivity of CA group with either Cys or His (Figures S26–S28 in Supporting Information).

Furthermore, covalent **20RNA_1A^CA^** ^‐**protein**^ conjugates were confirmed in case of HIV‐RT and HuR protein also by intact ESI‐MS analysis (Section 2.13 in Supporting Information, Figures S61–S64, S67, S68 in Supporting Information). Locations of cross‐linked amino acids in HIV‐RT, HuR and/or hAgo2 protein were determined by nano‐LC–MS/MS analysis of protein‐nucleic acids digests (Section 2.13 in Supporting Information, Figures S71–S83 in Supporting Information). For hAgo2 protein, the representative example of RBP, we compared positions of cross‐linked residues with available crystal structure to infer any general relationship between covalent capture and position of the residue within RBP. Five, out of total 22 Cys present in hAgo2 protein cross‐linked. All captured residues are in neighborhood of RNA (7–18 Å, Figure S84 in Supporting Information). However, since other Cys in close distance were not captured in none of three performed experimental replicates, we hypothesize that suitable spatial orientation and accessibility of the amino acid and position of the modification are important factors for efficient cross‐linking (Section 2.13.9.1. in Supporting Information). Similar observations were made for HuR protein (Figure S85 in Supporting Information), in HIV‐RT all Cys were cross‐linked and are in proximity to RNA (Figure S86 in Supporting Information).

To prove that the covalent bond between modified RNA and proteins is selectively formed due to proximity effect, we performed a control reaction with various weakly‐ or non‐RBPs containing Cys and/or His (Section 2.11.3 in Supporting Information). We tested bovine serum albumin (BSA), single strand binding protein (SSB), galectin 1 (Gal1), lysozyme (lysoz.) and human recombinant histone H2A (H2A). These proteins gave no or only negligible conversions of cross‐linking products **21RNA_1A^CA‐BSA^‐Cy5** (0 %), **21RNA_1A^CA‐SSB^‐Cy5** (2 %), **21RNA_1A^CA‐Gal1^‐Cy5** (0 %), **21RNA_1A^CA‐lysoz^‐Cy5** (3 %) with exception of histone H2A that gave 9 % of **21RNA_1A^CA‐H2A^‐Cy5** conjugate according to SDS‐PAGE analysis (Figure S22 in Supporting Information) and ImageJ quantification. Hence, the formation of covalent bond between modified RNA and the protein of interest requires strong proximity effect of a RBP and presence of Cys or His in the recognition site.

To demonstrate that our probes are functional in complex environment, we performed incubation of both modified **36RNA_1A^CA^‐Cy5** and non‐modified **36RNA_1A‐Cy5** with HeLa cell lysate. SDS‐PAGE analysis (Figure S25 in Supporting Information) revealed presence of several cross‐linked products in experiment with modified RNA, but not with unmodified control probe. Next, we aimed to elucidate, whether cross‐linking even in cell lysate is dependent on binding affinity. HuR protein is known to tightly bind AU‐rich sequences, while binding to CG‐rich RNA is significantly weaker.^[31]^ To this end, we prepared a set of two modified probes **21RNA_3A^CA^‐bind** (AU‐rich) and **21RNA_3A^CA^‐non‐bind** (GC‐rich) as well as two controls of unmodified probes **21RNA_3A‐bind** (AU‐rich) and **21RNA_3A‐non‐bind** (GC‐rich) (Section 2.3 in Supporting Information). Each of them was incubated with HeLa cell lysate. SDS‐PAGE followed by western blot analysis against HuR protein revealed presence of major slower moving band (at ≈250 kDa, due to formation of HuR‐RNA linear oligomers,^[32]^ Figure S30 in Supporting Information) in reaction with modified HuR‐binding AU‐rich **21RNA_3A^CA^‐bind**, while other three RNA controls did not produce any shifted bands (Figure S29 in Supporting Information). This demonstrates that the cellular HuR protein was selectively cross‐linked to binding modified RNA probe, while modified non‐binding or non‐modified RNA probes remained unreacted. These experiments pave the way to further advanced applications of our toolbox in RNA proteomics and selective probes for target proteins.

In conclusion, we designed and prepared modified 7‐deazaadenosine rNTP bearing highly reactive CA moiety, which served as a good substrate for T7 RNAP in enzymatic construction of various reactive RNA probes with one or several modifications at specific positions. (CA)‐modified RNA probes reacted with excess of thiol‐containing molecules and/or Cys‐ or His‐containing positively charged peptides to form stable covalent conjugates. (CA)‐modified RNA probes also reacted selectively with RBPs under physiological conditions and formation of covalent conjugates was highly enhanced by proximity effect. RNA‐protein cross‐linked conjugates were successfully characterized by intact mass analysis and proteomic nano‐LC–MS/MS. Our novel method enables to pinpoint exact RNA binding sites within a protein without any need of external cross‐linking reagents or UV source for activation of the reactive moiety for cross‐linking, thereby overcoming UV‐related damage and toxicity issues. It also could be useful for labelling of RNA with fluorophores, biotin or other tags. Most importantly, our study paves the way for further extension of this method to *in cellulo* RNA proteomic studies, which are currently ongoing and for modifying the poly(A) tail in messenger RNAs with binding sites for a major class of regulatory factors, the poly(A) binding proteins.

## Conflict of interest

The authors declare no conflict of interest.

## Supporting information

As a service to our authors and readers, this journal provides supporting information supplied by the authors. Such materials are peer reviewed and may be re‐organized for online delivery, but are not copy‐edited or typeset. Technical support issues arising from supporting information (other than missing files) should be addressed to the authors.

Supporting Information

## Data Availability

The data that support the findings of this study are available in the Supporting Information of this article.
