# Supplementary material for: Chloroacetamide‐Modified Nucleotide and RNA for Bioconjugations and Cross‐Linking with RNA‐Binding Proteins
Source: Angew Chem Int Ed Engl. 2023 Jan 12;62(7):e202213764. doi: 10.1002/anie.202213764 (PMC10107093; doi:10.1002/anie.202213764)
Supplement: Supplementary file 1 — Supporting Information [file ANIE-62-0-s001.pdf]

Angewandte Chemie
Eine Zeitschrift der Gesellschaft Deutscher Chemiker

Supporting Information

Chloroacetamide-Modified Nucleotide and RNA for Bioconjugations and Cross-Linking with RNA-Binding Proteins

*M. Brunderová, M. Krömer, M. Vlková, M. Hocek**

Supporting Information

Chloroacetamide-Modified Nucleotide and RNA for Bioconjugations and Cross-Linking with RNA-Binding Proteins

*Mária Brunderová, Matouš Krömer, Marta Vlková, and Michal Hocek**

Table of contents

1 Synthetic part 5

1.1 General remarks 5

1.2 Chemical synthesis 5

1.3 Copies of NMR spectra 7

1.4 Copies of MS ESI⁻ spectra 9

2 Biochemical part 10

2.1 General remarks 10

2.2 List of DNA oligonucleotide sequences used in this study 11

2.3 List of RNA oligonucleotide sequences synthesized in this study 11

2.4 Methods for preparation of RNA-binding proteins (RBPs) and weakly- or non-RBP used in this study and preparation of HeLa cell lysate 12

2.4.1 Preparation of HIV reverse transcriptase (HIV-RT)[5] 12

2.4.2 Preparation of human antigen R protein (HuR)[6] 12

2.4.3 Preparation of galectin 1 protein (Gal1) 13

2.4.4 Preparation and quantification of HeLa cell lysate 14

2.5 General procedures 14

2.5.1 General purification procedure I (silica spin columns) 14

2.5.2 General purification procedure II (gel filtration spin columns) 14

2.5.3 General purification procedure III (gel extraction) 15

2.6 Enzymatic synthesis of natural and modified RNAs *via* transcription 15

2.6.1 Preparation of double stranded DNA (dsDNA) templates for transcription reaction 15

2.6.2 General procedure for denaturing polyacrylamide gel electrophoresis (dPAGE) analysis 15

2.6.3 Analysis of cross-linking of modified 20RNA_1A^{CA} to T7 RNA polymerase by denaturing SDS-PAGE 15

2.6.4 Denaturing PAGE analysis of inhibition of *in vitro* transcription reaction by rA^{CA}TP 16

2.6.4.1 Supplementary note no. 1 – Discussion of possible cross-linking with T7 RNAP 17

2.6.5 Incorporation of rA^{CA}TP using 20DNA_1A template in analytical scale for dPAGE analysis 18

2.6.6 Incorporation of rA^{CA}TP using 35DNA_1A template in analytical scale for dPAGE analysis 18

2.6.7 Incorporation of rA^{CA}TP using 35DNA_3A and/or 35DNA_7A template in analytical scale for dPAGE analysis 19

2.6.8 Enzymatic synthesis of 20RNA_1A or 20RNA_1A^{CA} in semi-preparative scale....20
2.6.9 Enzymatic synthesis of 35RNA_1A or **35RNA_1A^{CA} in semi-preparative scale**....21
2.6.10 Enzymatic synthesis of 35RNA_3A^{CA} and 35RNA_7A^{CA} in semi-preparative scale. 21
2.6.11 Enzymatic synthesis of 21RNA_3A-bind, 21RNA_3A^{CA}-bind and 21RNA_3A-non-bind, 21RNA_3A^{CA}-non-bind in preparative scale 21
2.6.12 Enzymatic synthesis of 20RNA_1A or 20RNA_1A^{CA} in preparative scale 22
2.6.13 Enzymatic synthesis of 35RNA_1A or 35RNA_1A^{CA} in preparative scale 22
2.6.14 Enzymatic synthesis of 35RNA_3A^{CA} or 35RNA_7A^{CA} in preparative scale....22
2.7 RNA labelling *via* ligation reactions....23
2.7.1 Structures of pCp-Cy5 and pCp-Bio....23
2.7.2 Preparation of 21RNA_1A-Cy5 and 21RNA_1A^{CA}-Cy5 using pCp-Cy5....23
2.7.3 Preparation of 21RNA_1A-Bio and 21RNA_1A^{CA}-Bio using pCp-Bio....23
2.7.4 Preparation of 36RNA_1A-Cy5 and 36RNA_1A^{CA}-Cy5 using pCp-Cy5....24
2.7.5 Preparation of 36RNA_3A^{CA}-Cy5 and 36RNA_7A^{CA}-Cy5 using pCp-Cy5....24
2.8 Bioconjugation reactions, denaturing PAGE and mass analysis....26
2.8.1 Structures of peptides and other (bio)molecules used in this study 26
2.8.2 Bioconjugation of natural or modified RNA with either glutathione (GSH), pept-(+)-H or biotin-thiol in analytical scale for dPAGE analysis 26
2.8.3 Bioconjugation of natural or modified RNA with either pept-(+)-C or pept-(–)-C in analytical scale for dPAGE analysis....27
2.8.4 Bioconjugation of natural or modified RNA with either pept-K or pept-R in analytical scale for dPAGE analysis....27
2.8.5 Bioconjugation of natural or modified RNA with fluorescein-thiol in analytical scale for dPAGE analysis 27
2.8.6 Bioconjugation of modified RNA with increasing concentration of pept-(+)-C in analytical scale for dPAGE analysis....30
2.8.7 Bioconjugation of modified RNA either with glutathione (GSH) or pept-(+)-H in semi-preparative scale for MS-MALDI-TOF analysis 31
2.8.8 Bioconjugation of modified RNA with pept-(+)-C in semi-preparative scale for MS-MALDI-TOF analysis 31
2.8.9 Bioconjugation of modified RNA with biotin-thiol in semi-preparative scale for MS-MALDI-TOF analysis 31
2.8.10 Bioconjugation of modified RNA with fluorescein-thiol in semi-preparative scale for MS-MALDI-TOF analysis 31
2.8.11 Bioconjugation of modified RNA with pept-(+)-C in semi-preparative scale for LC-MS analysis....32
2.9 Fluorescence measurements....33
2.9.1 Bioconjugation and fluorescence measurements of either natural or modified RNA with fluorescein-thiol....33
2.10 Electrophoretic mobility shift assays (EMSA) 34

2.10.1 EMSA of natural or modified RNA with human antigen R (HuR) 34
2.10.2 EMSA of natural or modified RNA with HIV reverse transcriptase (HIV-RT) ... 34
2.10.3 EMSA of natural or modified RNA with human argonaute 2 protein (hAgo2) 35
2.11 Cross-linking reactions of RNA with proteins and SDS-PAGE analysis 36
2.11.1 Kinetic study of cross-linking reaction of modified RNA with HIV reverse transcriptase (HIV-RT) 36
2.11.2 Cross-linking reaction of natural or modified RNA with RNA-binding proteins (RBPs) 37
2.11.3 Cross-linking reactions of modified RNA with HuR protein and weakly- or non-RNA-binding proteins (non-RBPs) 39
2.11.4 Cross-linking reactions of either natural 36RNA_1A-Cy5 or modified 36RNA-1A^{CA}-Cy5, 36RNA-3A^{CA}-Cy5 and 36RNA-7A^{CA}-Cy5 with HuR protein 40
2.11.5 Cross-linking reactions of either natural 36RNA_1A-Cy5 or modified 36RNA-1A^{CA}-Cy5, 36RNA-3A^{CA}-Cy5 and 36RNA-7A^{CA}-Cy5 with bovine serum albumin 40
2.11.6 Cross-linking reactions of either natural 36RNA_1A-Cy5 or modified 36RNA-1A^{CA}-Cy5 with HeLa cell lysate proteins 41
2.12 Western-blot (WB) analysis of cross-linking reactions 42
2.12.1 WB analysis of cross-linking reaction of natural or modified RNA with HuR protein 42
2.12.2 WB analysis of cross-linking reaction of natural or modified RNA with HIV-RT 43
2.12.3 WB analysis of cross-linking reaction of biotinylated natural or modified RNA with hAgo2 protein 44
2.12.4 Selective targeting of HuR protein by cross-linking reaction in HeLa cell lysate . 45
2.13 Characterization of RNA-protein conjugates by mass analysis (intact ESI-MS or nano-LC-MS/MS) 47
2.13.1 General procedure for intact ESI-MS analysis of RNA-protein conjugates 47
2.13.2 General procedure I for nano-LC-MS/MS analysis of proteolytic digests 48
2.13.3 General procedure II for nano-LC-MS/MS analysis of proteolytic digests 48
2.13.4 Intact ESI-MS analysis of 20RNA_1A$^{CA\text{-}HuR}$ conjugate 48
2.13.5 Intact ESI-MS analysis of 20RNA_1A$^{CA\text{-}HIV\text{-}RT}$ conjugate 49
2.13.6 LC-MS analysis of conjugation mixture of 20RNA_1A^{CA} with HIV-RT protein . 49
2.13.7 Preparation of 20RNA_1A$^{CA\text{-}HuR}$ conjugate digest for nano-LC-MS/MS analysis 50
2.13.8 Preparation of 20RNA_1A$^{CA\text{-}HIV\text{-}RT}$ conjugate digest for nano-LC-MS/MS analysis 51
2.13.9 Preparation of 20RNA_1A$^{CA\text{-}hAgo2}$ conjugate digest for nano-LC-MS/MS analysis 51
2.13.9.1 Supplementary note no. 2 – Discussion of the specificity of the cross-linking with different cysteins of hAgo2 52
3 Copies of mass spectra 54
3.1 Copies of MS-MALDI-TOF spectra 54

3.2 Copies of ESI-MS spectra 62
3.3 Copies of nano-LC-MS/MS spectra 74
4 Additional information 81
4.1 Protein sequence of human antigen R protein (HuR) used in this study 81
4.2 Protein sequence of HIV reverse-transcriptase (HIV-RT) used in this study 81
4.3 Protein sequence of human argonaute 2 protein (hAgo2) used in this study 82
4.4 Protein sequence of galectin 1 protein (Gal1) used in this study 83
4.5 Mapping of crosslinked amino acids on crystal structures 84
5 References 87

1 Synthetic part

1.1 General remarks

All solvents and reagents were purchased from commercial suppliers (Fluorochem, Sigma Aldrich, Lach-Ner) and used as received without further purification, unless otherwise specified. Phosphoryl chloride ($POCl_3$) and trimethyl phosphate [$PO(OMe)_3$] were distilled prior to use. Reactions were performed in heat gun-dried glassware under argon atmosphere. Milli-Q water was used in the synthetic part. Reactions were monitored by thin layer chromatography (TLC) on TLC Silica gel 60 F254 (Merck) and detected by UV (254 nm) and by Advion Expression Compact Mass Spectrometer connected with Plate Express® TLC Plate Reader using electrospray ionization (ESI). NMR spectra were measured on a Bruker AVANCE 500 NMR spectrometer (^{1}H at 500.0 MHz, ^{13}C 125.7 MHz and ^{31}P at 202.3 MHz) in D_2O at 25°C, referenced to *tert*-butanol-d9 as an internal standard [δ (^{1}H) = 1.25 ppm, δ (^{13}C) = 31.6 ppm]. Chemical shifts are given in ppm (δ-scale) and coupling constants (J) are in Hz. Multiplicity of the peaks is as followed: s = singlet, d = doublet, t = triplet, dd= doublet of doublets, ddd = doublet of doublets of doublets, bpent = broad pentet. Complete assignment of all NMR signals was achieved by using a combination of H,H-COSY, H,C-HSQC and H,C-HMBC experiments. Labelling of NMR signals assignments corresponds to a numbering depicted in compound formulas. High-resolution mass spectra were measured on LTQ Orbitrap XL (Thermo Fisher Scientific). All mass spectra were acquired by the MS service at IOCB. Column chromatography was performed using silica gel (40-63 µm, Fluorochem) by flash liquid chromatography system (FLC) Teledyne ISCO Combi Flash Rf 200 or 300. Purification of the ribonucleoside triphosphate was performed using HPLC (Waters modular HPLC system) on a Phenomenex Kinetex 5 µm EVO C18 100 Å, AXIA Packed LC column (250 X 21.2 mm) and POROS HQ 50 column (packed in our laboratory, 26 X 120 mm). Purity of all final compounds was determined by NMR spectroscopy.

1.2 Chemical synthesis

7-{3-[*N*-(2-Chloroacetamido)]-prop-2-yn-1-yl}-7-deazaadenosine-5'-*O*-triphosphate (rA^{CA}TP)

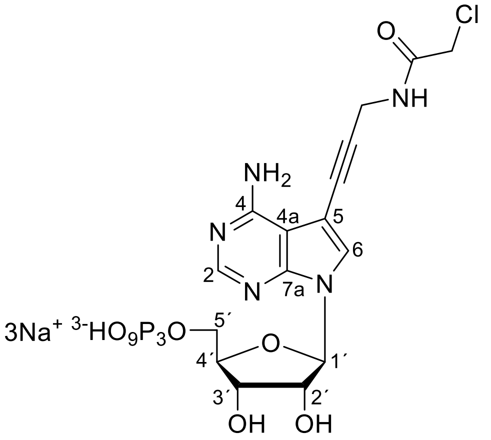

Iodo-modified ribonucleoside triphosphate in sodium form **rA^{I}TP**[1] (30 mg, 43.0 µmol, 1 equiv.), *N*-(propargyl)chloroacetamide (**1**)[2] (14.1 mg, 107.2 µmol, 2.5 equiv.), CuI (0.82 mg, 4.3 µmol, 10 mol%), $Pd(OAc)_2$ (0.48 mg, 2.1 µmol, 5 mol%) and TPPTS (2.44 mg, 4.3 µmol, 10 mol%) were dissolved in a mixture of H_2O/acetonitrile (2:1, 1.5 mL) in a microwave vial containing a stir bar under argon atmosphere. *N*,*N*-diisopropylethylamine (75 µL, 0.43 mmol, 10 equiv.) was added *via* syringe and the mixture was stirred under argon atmosphere at 60 °C for 4 h. After the reaction, the mixture was cooled down, the solvents were removed under reduced pressure and the residue was again dissolved in 5 mL of H_2O and filtrated through 0.22 µm HPLC filter prior to purification. The HPLC separation was performed on C18 reversed phase column using linear gradient from 0.1 M TEAB (triethylammonium bicarbonate, aq.) to 0.1 M TEAB in 50% MeOH

followed by several co-evaporations with H_2O. The second purification was performed on POROS HQ 50 column using linear gradient from H_2O to 400 mM TEAB (aq.) followed by several co-evaporations with H_2O. The pure product was isolated as a triethylammonium salt and converted to sodium salt using Dowex 50WX8 in Na^+ cycle. The solvent was evaporated, pure product was again dissolved in small amount of H_2O and freeze-dried overnight. The desired product **rA^{CA}TP** (as sodium salt) was obtained as a white solid powder (7.3 mg, 24%), [Figures S1-S4].

^{1}H NMR (500 MHz, D$_2$O): 4.16 (ddd, 1H, J_{gem} = 11.7 Hz, $J_{5'a,P}$ = 4.6 Hz, $J_{5'a,4'}$ = 3.2 Hz, H-5´a); 4.20 (s, 2H, CH_2Cl); 4.28 (ddd, 1H, J_{gem} = 11.7 Hz, $J_{5'b,P}$ = 6.6 Hz, $J_{5'b,4'}$ = 3.0 Hz, H-5´b); 4.29 (s, 2H,C≡CC**H$_2$**); 4.36 (bpent, 1H, $J_{4',3'}$ = $J_{4',5'a}$ = $J_{4',5'b}$ = $J_{4',P}$ = 2.9 Hz, H-4´); 4.58 (dd, 1H, $J_{3',2'}$ = 5.3 Hz, $J_{3',4'}$ = 2.8 Hz, H-3´); 4.68 (dd, 1H, $J_{2',1'}$ = 6.8 Hz, $J_{2',3'}$ = 5.3 Hz, H-2´); 6.24 (d, 1H, $J_{1',2'}$ = 6.8 Hz, H-1´); 7.78 (s, 1H, H-6); 8.15 (s, 1H, H-2).

^{13}C NMR (125.7 MHz, D$_2$O): 32.28 (C≡C**C**H$_2$); 44.13 (CH_2Cl); 67.40 (d, $J_{C,P}$ = 5.5 Hz, CH$_2$-5´); 72.54 (CH-3´); 75.85 (CH-2´); 77.30 (**C**≡CCH$_2$); 85.84 (d, $J_{C,P}$ = 9.1 Hz, CH-4´); 87.78 (CH-1´); 89.75 (C≡**C**CH$_2$); 98.46 and 105.14 (C-5,4a); 128.39 (CH-6); 151.35 (C-7a); 154.39 (CH-2); 159.46 (C-4); 171.89 (NHCO).

^{31}P NMR (202.4 MHz, D$_2$O): –21.63 (t, 1P, $J_{\beta,\alpha}$ = $J_{\beta,\gamma}$ = 19.8 Hz, P$_\beta$); –10.46 (d, 1P, $J_{\alpha,\beta}$ = 19.8 Hz, P$_\alpha$); –7.14 (d, 1P, $J_{\gamma,\beta}$ = 19.8 Hz, P$_\gamma$).

HR MS (ESI$^–$) of triphosphate: $C_{16}H_{20}O_{14}N_5(^{35}Cl)P_3$: calculated: 633.99023, found: 633.99023; **$C_{16}H_{20}O_{14}N_5(^{37}Cl)P_3$:** calculated: 635.98786, found: 635.98682.

HR MS (ESI$^–$) of diphosphate: $C_{16}H_{19}O_{11}N_5(^{35}Cl)P_2$: calculated: 554.02503, found: 554.02415; **$C_{16}H_{19}O_{11}N_5(^{37}Cl)P_2$:** calculated: 556.02153, found: 556.02139; **$C_{16}H_{18}O_{11}N_5(^{35}Cl)NaP_2$:** calculated: 576.00697, found: 576.00594; **$C_{16}H_{18}O_{11}N_5(^{37}Cl)NaP_2$:** calculated: 578.00348, found: 578.00276.

HR MS (ESI$^–$) of monophosphate: $C_{16}H_{18}O_8N_5(^{35}Cl)P$: calculated: 474.05870, found: 474.05783; **$C_{16}H_{18}O_8N_5(^{37}Cl)P$:** calculated: 476.05520, found: 476.05325.

MS (ESI$^–$): *m/z* (%):
327.5 [M (^{35}Cl)-3H+Na]$^{2–}$ (28); 576.0 [M (^{35}Cl)-PO$_3$H$_3$+Na]$^–$ (31); 556 [M (^{37}Cl)-PO$_3$H$_2$]$^–$ (34); 316.5 [M (^{35}Cl)-2H]$^{2–}$ (61); 554 [M (^{35}Cl)-PO$_3$H$_2$]$^–$ (100).

1.3 Copies of NMR spectra

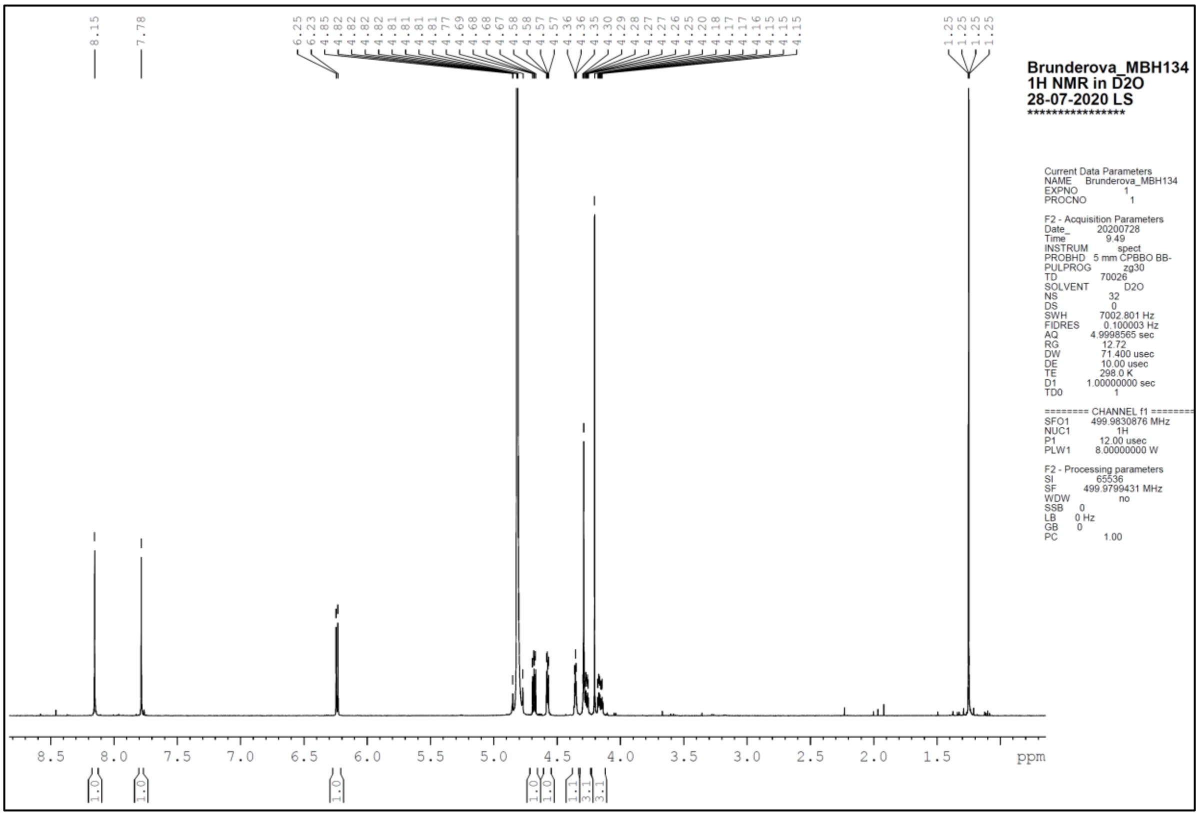

Figure S1. ^{1}H NMR spectrum of **rA$^{\mathbf{CA}}$TP**, full spectrum.

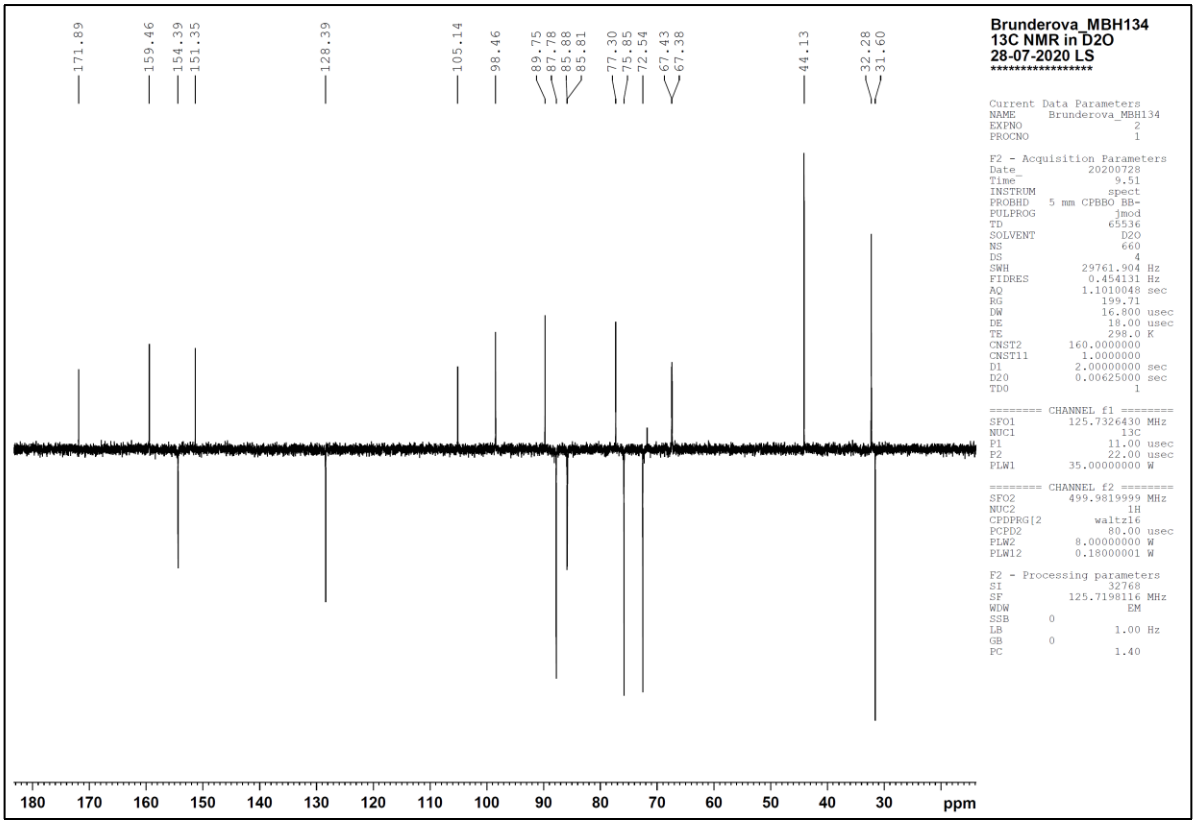

Figure S2. ^{13}C NMR spectrum of **rA$^{\mathbf{CA}}$TP**, full spectrum.

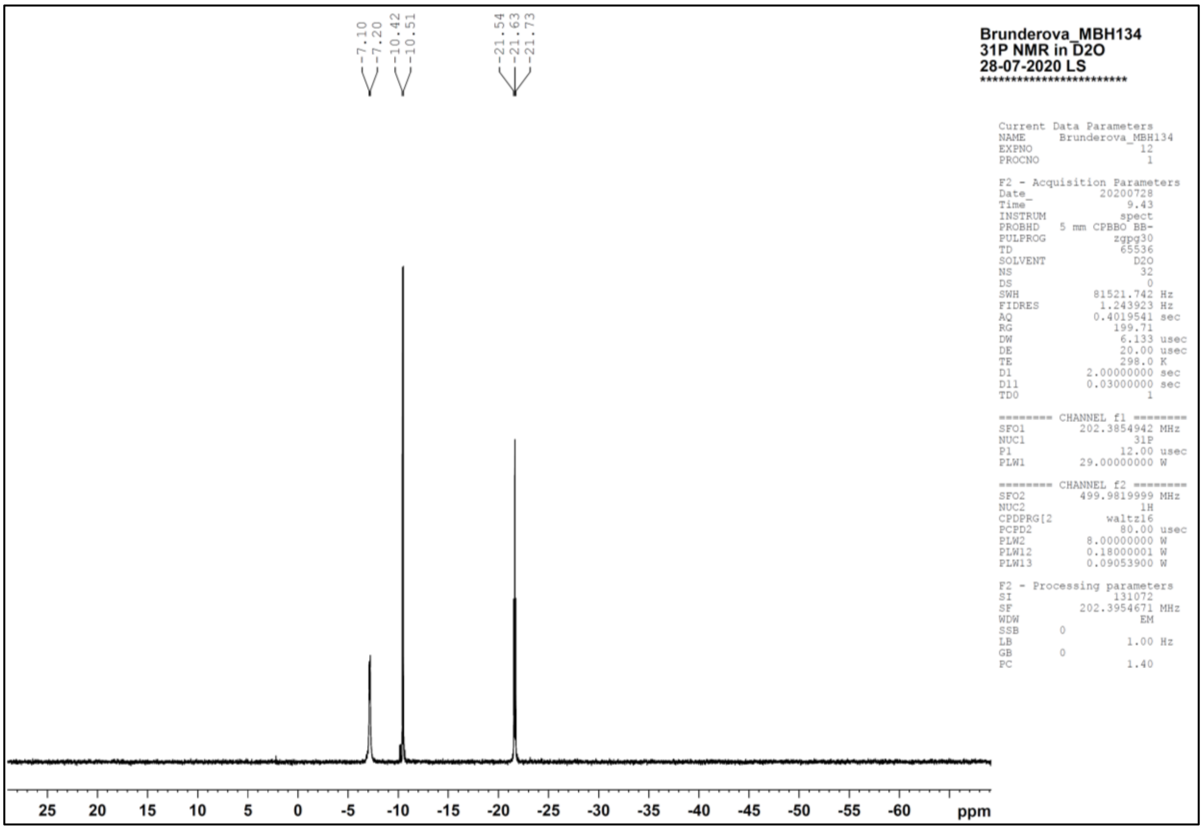

Figure S3 a). ^{31}P NMR spectrum of **rA^{CA}TP**, full spectrum.

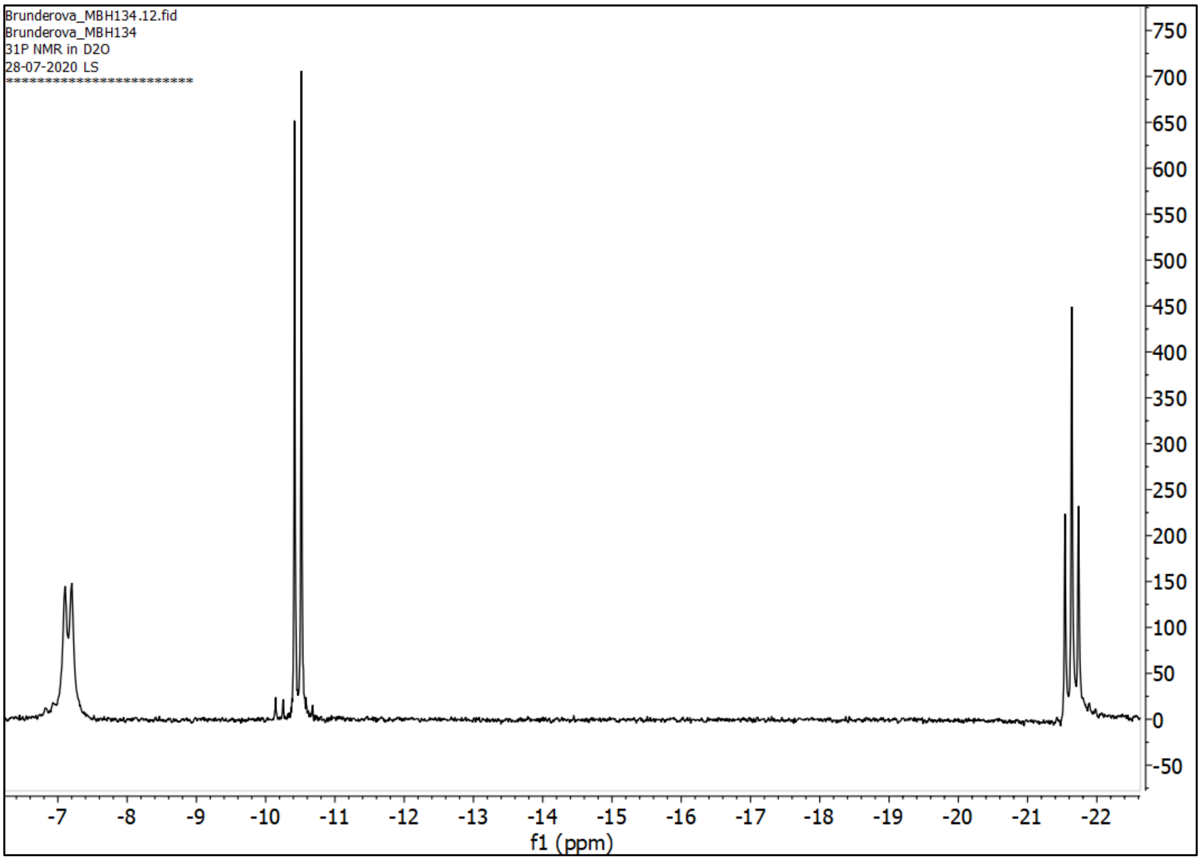

Figure S3 b). ^{31}P NMR spectrum of **rA^{CA}TP**, magnified area of interest.

1.4 Copies of MS ESI⁻ spectra

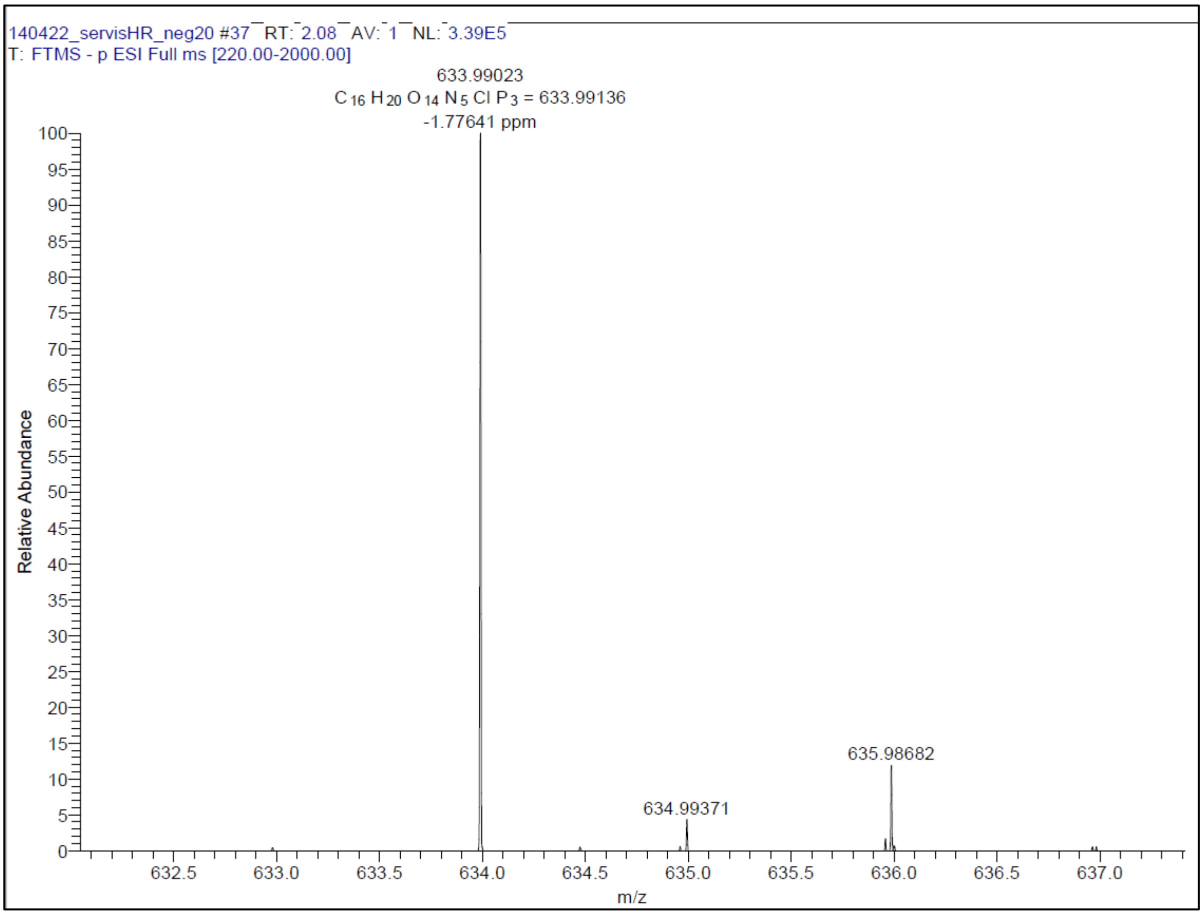

Figure S4. HR MS (ESI$^-$) spectrum of **rA^{CA}TP**, full spectrum.

2 Biochemical part

2.1 General remarks

All PAGE gels were analyzed by fluorescence and/or phosphor imaging using Typhoon FLA 9500 (GE Healthcare Life Sciences). Mass spectra of oligonucleotides were measured on UltrafleXtreme MALDI-TOF/TOF (Bruker) mass spectrometer with 1 kHz smartbeam II laser technology. The matrix consisted of 3-hydroxypicolinic acid (HPA)/picolinic acid (PA)/ammonium tartrate in ratio 9:1:1. The matrix (1 µL) was applied on the target (ground steel) and dried down at room temperature. The sample (1 µL) and the matrix (1 µL) were mixed and added on the top of the dried matrix preparation spot and dried down at room temperature. Samples were concentrated on CentriVap vacuum concentrator system (Labconco) and lyophilized on FreeZone 2.5 L freeze dryer (Labconco). Fluorescence spectra were measured in a 100 µL quartz cuvette at room temperature on a Fluoromax 4 spectrofluorometer (HORIBA Scientific). Single stranded DNA oligonucleotides for preparation of double stranded DNA templates were purchased from Generi Biotech. DNase I, T7 RNA polymerase with the corresponding transcription buffer, RiboLock RNase Inhibitor, Page Blue protein gel staining solution, proteases (Pierce trypsin protease, Pierce chymotrypsin protease, MS grade quality) and C18 spin columns were obtained from Thermo Fisher Scientific. The natural ribonucleoside triphosphates (rNTPs), protein ladder [Color Prestained Protein Standard, Broad Range (10-250 kDa)], T4 RNA ligase 1 and the corresponding ligase buffer and additives for ligation reaction and Monarch RNA purification kits (10 µg and 50 µg) were purchased from New England Biolabs. ZR small-RNA PAGE recovery kit was purchased from Zymo research. Microspin G-25 columns and Amicon ultra-0.5 centrifugal concentrator (10 kDa MWCO) were purchased from Merck. [α-^{32}P]-GTP (111 TBq/mmol, 370 MBq/mL) was obtained from MGP. The pCp-Cy5 (cytidine-5'-phosphate-3'-(6-aminohexyl)phosphate, labeled with Cy5) and pCp-biotin (cytidine-5'-phosphate-3'-(6-aminohexyl)phosphate, labeled with biotin) were purchased from Jena Bioscience. Fluorescein-thiol was purchased from BioActs and Biotin-thiol from Polypure. Peptides were prepared by automated solid-phase synthesis according to standard procedures, HPLC purified and characterized by MS-MALDI-TOF at IOCB. RNase/DNase free solutions for biochemical reactions were prepared using Milli-Q water, that was treated with DEPC and sterilized by autoclaving. Concentrations of the prepared RNA solutions were calculated using extinction coefficients obtained from on-line tool at https://www.atdbio.com/tools/oligo-calculator and A_{260} values measured on Nanodrop 1000 (Thermo Fischer Scientific). Intact ESI-MS analysis was carried out on AQUITY UPLC I-Class system (Waters) coupled to mass spectrometer Synapt G2 (Waters). The nano-LC-MS/MS analysis was performed on UltiMate 3000 RSLCnano system (Thermo Fisher Scientific) coupled to a mass spectrometer Orbitrap Fusion Lumos Tribrid (Thermo Fisher Scientific). LC-ESI-MS spectra were acquired on Agilent 1290 Infinity II Bio system with DAD detector and mass spectrometer MSD XT. LC-ESI-MS analysis of oligonucleotides were carried out according to standard procedures using mobile phases A (12.2 mM Et_3N, 300 mM HFIP in H_2O) and B (12.2 mM Et_3N, 300 mM HFIP in H_2O in 100% MeOH) by 10 min gradient from 5% B to 100% B in A using bioZen 1.7 µm oligo column 2.1 X 50 mm (Phenomenex) on Agilent UHPLC Bio system. Deconvolutions of LC-ESI-MS spectra were carried out using UniDec program[3]. Figure S30 was created in BioRender.

2.2 List of DNA oligonucleotide sequences used in this study

Name	Sequence (5´ → 3´)	Length
**20DNA_1A-s**	*TAATACGACTCACTATA*GGGCCCCTATTGTCTCTCTC	**37 nt**
**20DNA_1A-as**	[mG][mA]GAGAGACAATAGGGGCCC*TATAGTGAGTCGTATTA*	**37 nt**
**20DNA_1A-ds**	*TAATACGACTCACTATA*GGGCCCCTATTGTCTCTCTC<br>*ATTATGCTGAGTGATAT*CCCGGGGATAACAGAGAG[mA][mG]	**37 bp**
**35DNA_1A-s**	*TAATACGACTCACTATA*GGGCCCCTATTGTCTCTCTCTTCTCTGCTGTTTCC	**52 nt**
**35DNA_1A-as**	[mG][mG]AAACAGCAGAGAAGAGAGAGACAATAGGGGCCC*TATAGTGAGTCGTATTA*	**52 nt**
**35DNA_1A-ds**	*TAATACGACTCACTATA*GGGCCCCTATTGTCTCTCTCTTCTCTGCTGTTTCC<br>*ATTATGCTGAGTGATAT*CCCGGGGATAACAGAGAGAGAAGAGACGACAAA[mG][mG]	**52 bp**
**35DNA_3A-s**	*TAATACGACTCACTATA*GGGCCCGTATGTTACTTGCTCTTATCGTCTCTCGC	**52 nt**
**35DNA_3A-as**	[mG][mC]GAGAGACGATAAGAGCAAGTAACATACGGGCCC*TATAGTGAGTCGTATTA*	**52 nt**
**35DNA_3A-ds**	*TAATACGACTCACTATA*GGGCCCGTATGTTACTTGCTCTTATCGTCTCTCGC<br>*ATTATGCTGAGTGATAT*CCCGGGCATACAATGAACGAGAATAGCAGAGAG[mC][mG]	**52 bp**
**35DNA_7A-s**	*TAATACGACTCACTATA*GGGCTTGCACGTGAATCGCTCTTAATGGATCGCGA	**52 nt**
**35DNA_7A-as**	[mU][mC]GCGATCCATTAAGAGCGATTCACGTGCAAGCCC*TATAGTGAGTCGTATTA*	**52 nt**
**35DNA_7A-ds**	*TAATACGACTCACTATA*GGGCTTGCACGTGAATCGCTCTTAATGGATCGCGA<br>*ATTATGCTGAGTGATAT*CCCGAACGTGCACTTAGCGAGAATTACCTAGCG[mC][mU]	**52 bp**
**21DNA_3A-bind-s**	*TAATACGACTCACTATA*GGGTGATTTTATTTTATTCTC	**38 nt**
**21DNA_3A-bind-as**	[mG][mA]GAATAAAATAAAATCACCC*TATAGTGAGTCGTATTA*	**38 nt**
**21DNA_3A-bind-ds**	*TAATACGACTCACTATA*GGGTGATTTTATTTTATTCTC<br>*ATTATGCTGAGTGATAT*CCCACTAAAATAAAATAAG[mA][mG]	**38 bp**
**21DNA_3A-non-bind-s**	*TAATACGACTCACTATA*GGGTCACGTGACGCCAGTCCC	**38 nt**
**21DNA_3A-non-bind-as**	[mG][mG]GACTGGCGTCACGTGACCC*TATAGTGAGTCGTATTA*	**38 nt**
**21DNA_3A-non-bind-ds**	*TAATACGACTCACTATA*GGGTCACGTGACGCCAGTCCC<br>*ATTATGCTGAGTGATAT*CCCAGTGCACTGCGGTCAG[mG][mG]	**38 bp**

s = sense strand of the double stranded DNA template
as = anti-sense strand of the double stranded DNA template
ds = double stranded DNA template
Promoter region is highlighted in italics.
Last two nucleotides at the 5´-terminus in the anti-sense strand are 2´-*O*-Me modified [mN; N = any nucleotide] to minimize non-templated nucleotide addition.[4]

2.3 List of RNA oligonucleotide sequences synthesized in this study

Name	Sequence (5´ → 3´)	Length
**35RNA_1A^{CA}**	pppGGGCCCCU**A**^{CA}UUGUCUCUCUCUUCUCUGCUGUUUCC	**35 nt**
**36RNA_1A^{CA}-Cy5**	pppGGGCCCCU**A**^{CA}UUGUCUCUCUCUUCUCUGCUGUUUCCC-Cy5	**36 nt**
**35RNA_3A^{CA}**	pppGGGCCCGU**A**^{CA}UGUU**A**^{CA}CUUGCUCUU**A**^{CA}UCGUCUCUCGC	**35 nt**
**36RNA_3A^{CA}-Cy5**	pppGGGCCCGU**A**^{CA}UGUU**A**^{CA}CUUGCUCUU**A**^{CA}UCGUCUCUCGCC-Cy5	**36 nt**
**35RNA_7A^{CA}**	pppGGGCUUGC**A**^{CA}CGUG**A**^{CA}**A**^{CA}UCGCUCUU**A**^{CA}**A**^{CA}UGG**A**^{CA}UCGCG**A**^{CA}	**35 nt**
**36RNA_7A^{CA}-Cy5**	pppGGGCUUGC**A**^{CA}CGUG**A**^{CA}**A**^{CA}UCGCUCUU**A**^{CA}**A**^{CA}UGG**A**^{CA}UCGCG**A**^{CA}C-Cy5	**36 nt**
**20RNA_1A^{CA}**	pppGGGCCCCU**A**^{CA}UUGUCUCUCUC	**20 nt**
**21RNA_1A^{CA}-Cy5**	pppGGGCCCCU**A**^{CA}UUGUCUCUCUCC-Cy5	**21 nt**
**21RNA_1A^{CA}-Bio**	pppGGGCCCCU**A**^{CA}UUGUCUCUCUCC-Bio	**21 nt**
**21RNA_3A^{CA}-bind**	pppGGGUG**A**^{CA}UUUU**A**^{CA}UUUU**A**^{CA}UUCUC	**21 nt**
**21RNA_3A^{CA}-non-bind**	pppGGGUC**A**^{CA}CGUG**A**^{CA}CGCC**A**^{CA}GUCCC	**21 nt**
**35RNA_1A**	pppGGGCCCCUAUUGUCUCUCUCUUCUCUGCUGUUUCC	**35 nt**
**36RNA_1A-Cy5**	pppGGGCCCCUAUUGUCUCUCUCUUCUCUGCUGUUUCCC-Cy5	**36 nt**
**35RNA_3A**	pppGGGCCCGUAUGUUACUUGCUCUUAUCGUCUCUCGC	**35 nt**
**35RNA_7A**	pppGGGCUUGCACGUGAAUCGCUCUUAAUGGAUCGCGA	**35 nt**
**20RNA_1A**	pppGGGCCCCUAUUGUCUCUCUC	**20 nt**
**21RNA_1A-Cy5**	pppGGGCCCCUAUUGUCUCUCUCC-Cy5	**21 nt**
**21RNA_1A-Bio**	pppGGGCCCCUAUUGUCUCUCUCC-Bio	**21 nt**
**21RNA_3A-bind**	pppGGGUGAUUUUAUUUUAUUCUC	**21 nt**
**21RNA_3A-non-bind**	pppGGGUCACGUGACGCCAGUCCC	**21 nt**

Modified bases in transcripts are marked in bold and blue, modification on the nucleobase is marked with bold and red.
ppp (triphosphate residue at the 5´-terminus of the RNA sequence).

2.4 Methods for preparation of RNA-binding proteins (RBPs) and weakly- or non-RBP used in this study and preparation of HeLa cell lysate

2.4.1 Preparation of HIV reverse transcriptase (HIV-RT)[5]

E. coli BL21-AI cells were co-transformed with pET21a(+)-HIV RT-6xHis-RBS-prot (Addgene #159149, a gift from Andrea Pauli) in 10 mL LB medium in presence of 0.8% glucose, 100 µg/mL AMP overnight at 37 °C. Cells were centrifuged and resuspended in approximately 1 mL LB medium. 4 flasks (each containing 1 L of Terrific broth modified with 0.5% glycerol, 100 µg/mL AMP) were inoculated with four aliquots (4X 0.25 mL) of starting culture and grown at 160 rpm, at 37 °C until OD_{600} = 1.7. Then the temperature was reduced to 30 °C and the protein expression was induced with 1 mM IPTG and 0.2% L-arabinose. The culture was grown with continuous shaking (160 rpm) overnight at 30 °C for additional 16 hours. Cells were harvested at OD_{600} = 8.0 at 8 000X g at 4 °C for 10 min. Pellets were stored at –80 °C prior to lysis and purification. Pellets were resuspended in approximately 500 mL HisTrap loading buffer (20 mM Tris, 500 mM NaCl, 1 mM DTT, pH = 7.8, 10% glycerol) and lysed by three passes on homogenizer CF-1 at 15-20 psi followed by centrifugation at 38 420X g for 30 min at 4 °C. First purification using ÄKTA pure was performed on HisTrap HP column (5 mL) by gradient of 0 to 500 mM imidazole in loading buffer (500 mM imidazole, 20 mM Tris, 500 mM NaCl, 1 mM DTT, pH = 7.8, 10% glycerol). Fractions containing the desired protein were determined by 10% denaturing Tris-Gly-sodium dodecyl sulfate-polyacrylamide gel electrophoresis (denaturing Tris-Gly-SDS-PAGE, 37.5:1 mono:bis acrylamide, 375 mM Tris, 0.1% SDS) in 1X Tris-Gly-SDS running buffer at 180 V for 1.5 h at room temperature. The gel was stained with Page Blue staining solution and visualized by a fluorescent scanner using Cy5 channel scan. Fractions were pooled and diluted 1:5 with 25 mM Tris buffer, pH = 7.8 to get final 100 mM NaCl concentration. Solution was loaded on HiTrap Heparin HP column (5 mL) and eluted with gradient of 1X TBS to 1X TBS with 2 M NaCl, pH = 7.4. Eluted fractions containing the desired protein analyzed by 10% denaturing Tris-Gly-SDS-PAGE were concentrated on Vivaspin 6, 30 kDa MWCO. Concentrated fractions were then purified on Sephadex HiLoad 200 pg, 16/600 running in 40 mM HEPES-NaOH, 250 mM KCl, 2 mM EDTA, 2 mM TCEP, pH = 7.5. Eluted fractions containing the desired product (as a hetero and homodimer mixture) determined by 10% denaturing Tris-Gly-SDS-PAGE were again concentrated on Vivaspin 6, 30 kDa MWCO, combined with sterile glycerol to final 50% concentration, aliquoted, flash frozen in liquid nitrogen and stored at –80 °C prior to usage. For the final purity analysis 10 µL aliquot (5 µM) was combined with 2.5 µL of 5X SDS stop solution (500 mM DTT, 150 mM Tris, 20 mM EDTA, 10% SDS, 50% glycerol, pH = 6.8) and denatured at 95 °C for 5 min. 10 µL were analyzed by 15% denaturing Tris-Gly-SDS-PAGE (37.5:1 mono:bis acrylamide, 375 mM Tris, 0.1% SDS) in 1X Tris-Gly-SDS running buffer at 200 V for 1.5 h at room temperature. After that, the gel was stained with Page Blue staining solution and the gel was visualized by a fluorescent scanner with Cy5 channel scan. [Gel analysis: Figure S5].

2.4.2 Preparation of human antigen R protein (HuR)[6]

E. coli BL21-AI cells were co-transformed with pET28a His6HuR (Addgene #135943, a gift from Yimon Aye) in 10 mL LB medium in presence of 0.8% glucose, 30 µg/mL kanamycin overnight at 37 °C. Cells were centrifuged and resuspended in approximately 1 mL LB medium. 4 flasks (each containing 1 L of Terrific broth modified with 0.5% glycerol, 30 µg/mL kanamycin) were inoculated with four aliquots (4X 0.25 mL) of starting culture and grown

at 160 rpm at 37 °C until OD_{600} = 2.0. Then the temperature was reduced to 22 °C and the protein expression was induced with 1 mM IPTG and 0.2% L-arabinose. The culture was grown with continuous shaking (160 rpm) overnight at 22 °C for additional 16 h. Cells were harvested at OD_{600} = 5.0 at 8 000X g at 4 °C for 10 min. Pellets were stored at –80 °C prior to lysis and purification. Pellets were resuspended in approximately 400 mL HisTrap loading buffer (20 mM Tris, 500 mM NaCl, 1 mM DTT, pH = 7.8, 10% glycerol) and lysed by three passes on homogenizer CF-1 at 15-20 psi followed by centrifugation at 38 420X g for 30 min at 4 °C. First purification on ÄKTA pure was performed on HisTrap HP column (5 mL) by gradient of 0 to 500 mM imidazole in loading buffer (500 mM imidazole, 20 mM Tris, 500 mM NaCl, 1 mM DTT, pH = 7.8, 10% glycerol). Fractions containing the desired protein were determined by 10% denaturing Tris-Gly-SDS-PAGE (37.5:1 mono:bis acrylamide, 375 mM Tris, 0.1% SDS) in 1X Tris-Gly-SDS running buffer at 180 V for 1.5 h at room temperature. The gel was stained with Page Blue staining solution and visualized by a fluorescent scanner using Cy5 channel scan. Fractions were pooled and diluted 1:5 with 25 mM Tris buffer, pH = 7.8 to get final 100 mM NaCl concentration. Solution was loaded on HiTrap Heparin HP column (5 mL) and eluted with gradient of 1X TBS to 1X TBS with 2 M NaCl, pH = 7.4. Eluted fractions determined by 10% denaturing Tris-Gly-SDS-PAGE were concentrated on Vivaspin 6, 10 kDa MWCO in storage buffer (1X TBS, pH = 7.4, 250 mM NaCl), pooled, aliquoted, flash frozen in liquid nitrogen and stored at –80 °C prior to usage. For the final purity analysis 10 µL aliquot (5 µM) was combined with 2.5 µL of 5X SDS stop solution (500 mM DTT, 150 mM Tris, 20 mM EDTA, 10% SDS, 50% glycerol, pH = 6.8) and denatured at 95 °C for 5 min. 10 µL were analyzed by 15% denaturing Tris-Gly-SDS-PAGE (37.5:1 mono:bis acrylamide, 375 mM Tris, 0.1% SDS) in 1X Tris-Gly-SDS running buffer at 200 V for 1.5 h at room temperature. After that, the gel was stained with Page Blue staining solution and the gel was visualized by a fluorescent scanner with Cy5 channel scan. [Gel analysis: Figure S5].

2.4.3 Preparation of galectin 1 protein (Gal1)

E. coli BL21(DE3) containing pET-based plasmid bearing gene for Galectin 1 with C-terminal AviTag followed by 6X HisTag and pLEMO BirA plasmid (Addgene #119818, a gift from Alexander Gabibov) were grown overnight at 30 °C and 140 rpm in 100 mL LB medium containing ampicillin (100 µg/mL) and chloramphenicol (37 µg/mL). Cells were centrifuged and inoculated in 1 L of LB medium with antibiotics (ampicillin 100 µg/mL, chloramphenicol 37 µg/mL) and grown at 30 °C, 140 rpm until OD_{600} = 0.4. Induction of biotin ligase was performed with 0.2% L-rhamnose and the medium was supplemented with 0.1 mM biotin. After next 2.5 h, galectin expression was triggered by 1 mM IPTG. After next 3.5 h the bacteria were harvested, resuspended in loading buffer (20 mM Tris, 500 mM NaCl, 10% glycerol, pH = 8.0) and sonicated on ice by 2 sec bursts (40% amplitude), 15 sec pause for total 10 min. The resulting lysate was clarified by centrifugation at 50 000X g at 4 °C for 20 min. Supernatant (25 mL) was purified on ÄKTA pure using HisTrap FF column (1 mL). Elution was performed by gradient from 0 to 500 mM imidazole in loading buffer (500 mM imidazole, 20 mM Tris, 500 mM NaCl, 10% glycerol, pH = 8.0). Fractions containing the desired protein were determined by 15% denaturing Tris-Gly-SDS-PAGE (37.5:1 mono:bis acrylamide, 375 mM Tris, 0.1% SDS) in 1X Tris-Gly-SDS running buffer at 180 V for 2 h at room temperature. The gel was stained with Page Blue staining solution and visualized by a fluorescent scanner using Cy5 channel scan. Fractions were pooled, concentrated on Vivaspin 20, 3 kDa MWCO in storage buffer (50 mM Tris, 137 mM NaCl, 2.7 mM KCl, pH = 7.6) and combined with sterile glycerol to final 50% concentration, aliquoted, flash frozen in liquid nitrogen and stored at –80 °C prior to usage. For the final purity analysis 10 µL aliquot (5 µM) was combined with 2.5 µL of 5X SDS stop solution (500 mM DTT, 150 mM Tris,

20 mM EDTA, 10% SDS, 50% glycerol, pH = 6.8) and denatured at 95 °C for 5 min. 10 μL were analyzed by 15% denaturing Tris-Gly-SDS-PAGE (37.5:1 mono:bis acrylamide, 375 mM Tris, 0.1% SDS) in 1X Tris-Gly-SDS running buffer at 200 V for 1.5 h at room temperature. After that, the gel was stained with Page Blue staining solution and the gel was visualized by a fluorescent scanner with Cy5 channel scan. [Gel analysis: Figure S5].

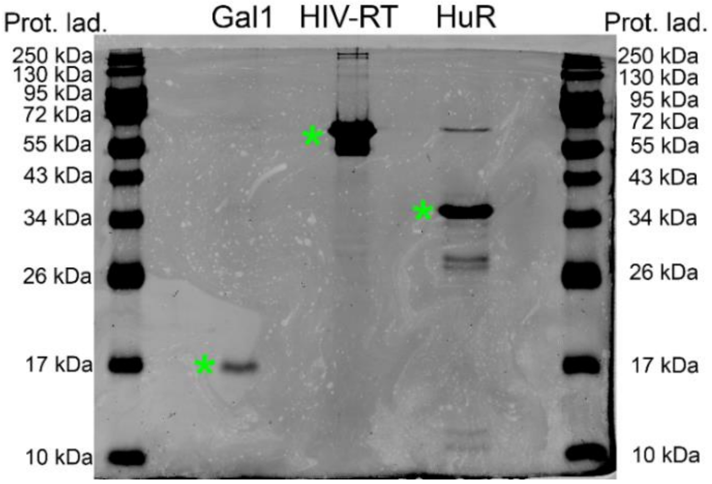

Figure S5. Final purity analysis of the prepared proteins. Cy5 channel scan of 15% denaturing Tris-Gly-SDS-PAGE. (Prot. lad.) pre-stained protein ladder; (HuR) human antigen R, protein mass: 38.5 kDa; (HIV-RT) HIV reverse transcriptase, protein mass: 65.5 kDa (large subunit) and 51.3 kDa (small subunit); (Gal1) galectin 1, protein mass: 17.6 kDa.

2.4.4 Preparation and quantification of HeLa cell lysate

Cells (3×10^7) were thawed on ice and resuspended in 150 μL of lysis buffer (50 mM Tris, 150 mM NaCl, 1% Triton X-100, 5 mM EDTA, pH = 7.4) containing HaltProtease inhibitor (1X, Thermo Fisher Scientific). Cell disruption was carried out by sonication by 3X 30 sec of 100% power bursts on ice (Hielscher digital ultrasonic generator UP200St) with tip probe. Cellular debris was removed at 20 000X g for 20 min at 4 °C. Proteins were quantified by QuantiPro BCA assay kit (Sigma Aldrich) with BSA as a standard.

2.5 General procedures

2.5.1 General purification procedure I (silica spin columns)

The samples (adjusted with H_2O to final volume 50 μL) were purified using the Monarch RNA purification kit (50 μg), according to standard supplier´s protocol. Elution of the samples was performed into DNase/RNase free eppendorf tube with 50 μL of H_2O.

2.5.2 General purification procedure II (gel filtration spin columns)

The Microspin G-25 columns were pre-washed with 2X 500 μL of H_2O. The analyzed sample (adjusted with H_2O to final volume 50 μL) was loaded on the column and purified according to standard supplier´s protocol. Elution of the samples was performed into DNase/RNase free eppendorf tube.

2.5.3 General purification procedure III (gel extraction)

The samples after preparative scale transcription reaction (400 μL) were mixed with 400 μL of 2X stop solution (95% [v/v] formamide, 0.5 mM EDTA, 0.025% [w/v] bromophenol blue, 0.025% [w/v] SDS in H_2O) and denatured by heating at 95 °C for 2 min followed by cooling on ice. Samples were loaded on preparative 22.5% denaturing polyacrylamide gel (dPAGE, 19:1 mono:bis acrylamide) containing 1X TBE buffer (pH = 8) and urea (7 M). Separation was performed at 20 mA for 2-3 h (until the dye migrated to the bottom third of the gel). The RNA transcripts were visualized by UV shadowing (254 nm) and the purification was performed according to standard supplier´s protocol using ZR small-RNA PAGE recovery kit (Zymo research). Elution of the RNA was performed into DNase/RNase free eppendorf tube with 2X 20 μL of H_2O. Purified RNAs were further used in labelling reactions (Section 2.7.2; 2.7.4; 2.7.5).

2.6 Enzymatic synthesis of natural and modified RNAs *via* transcription

2.6.1 Preparation of double stranded DNA (dsDNA) templates for transcription reaction

A solution of complementary single stranded DNA oligonucleotides (10 μL, 100 μM of each) in annealing buffer (10 mM Tris, 50 mM NaCl, 1 mM EDTA, pH = 7.8) was heated up to 95 °C for 5 min in a thermal cycler (with heated lid to 105 °C) and then slowly cooled down to 25 °C (0.02 °C/s^{-1}), (for DNA sequences see Section 2.2).

2.6.2 General procedure for denaturing polyacrylamide gel electrophoresis (dPAGE) analysis

The samples after transcription reaction (10 μL) were stopped by mixing with 2X stop solution (10 μL) containing 95% [v/v] formamide, 0.5 mM EDTA, 0.025% [w/v] bromophenol blue, 0.025% [w/v] SDS in H_2O. Samples were denatured by heating at 65 °C for 10 min and then immediately cooled on ice. Aliquots of the samples (10 μL) were subjected to vertical gel electrophoresis on denaturing polyacrylamide gel (dPAGE, 19:1 mono:bis acrylamide) containing 1X TBE buffer (pH = 8) and urea (7 M) at 42 mA for 30-40 min (until the dye migrated to the bottom third of the gel). The gel was then autoradiographed at least for 1 h and then visualized by a phosphor imager.

2.6.3 Analysis of cross-linking of modified 20RNA_1A^{CA} to T7 RNA polymerase by denaturing SDS-PAGE

In vitro transcription reactions were performed in total volume of 20 μL in transcription reaction buffer (5X, 4 μL) containing either natural rATP (2 mM) or modified **rA^{CA}TP** (2 mM), three natural rNTPs (2 mM, rCTP, UTP, rGTP), $MgCl_2$ (25 mM), RiboLock RNase inhibitor (1 U/μL), Triton X-100 (0.1%), dsDNA template (1.5 μM, **20DNA_1A**, prepared according to above-mentioned procedure, Section 2.6.1), T7 RNA polymerase (5 U/μL) and [α-^{32}P]-GTP (111 TBq/mmol, 370 MBq/mL, 0.2 μL). Transcription reactions were performed at 37 °C for 2 h in a thermal cycler with heated lid (75 °C). After this time, 10 μL aliquots of natural and modified RNAs were combined with 2.5 μL of 5X SDS stop solution (500 mM DTT, 150 mM Tris, 20 mM EDTA, 10% SDS, 50% glycerol, pH = 6.8) and denatured at 95 °C for 5 min. The remaining 10 μL aliquots of natural and modified RNAs were combined with

DNase I (1 μL, 1 U/μL) and further incubated at 37 °C for 15 min. After that, the samples were purified using Monarch RNA purification kit (10 μg), according to standard supplier´s protocol and eluted in 10 μL of H_2O. Eluted samples were combined with 2.5 μL of 5X SDS stop solution (500 mM DTT, 150 mM Tris, 20 mM EDTA, 10% SDS, 50% glycerol, pH = 6.8) and denatured at 95 °C for 5 min. All purified and non-purified samples of natural and modified RNAs were analyzed by 10% denaturing Tris-Gly-SDS-PAGE (37.5:1 mono:bis acrylamide, 375 mM Tris, 0.1% SDS) in 1X Tris-Gly-SDS running buffer at 180 V for 1 h at room temperature. The gel was then autoradiographed at least for 1 h and then visualized by a phosphor imager. [Gel analysis: Figure S6].

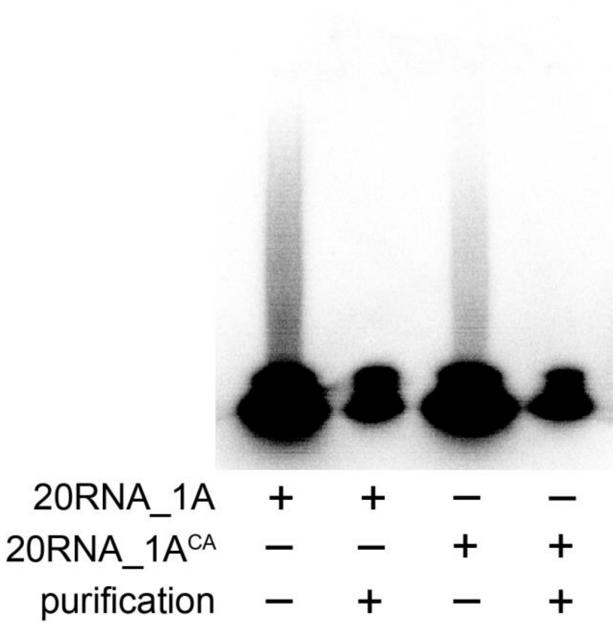

Figure S6. Phosphor imaging of 10% denaturing Tris-Gly-SDS-PAGE analysis. Comparison of T7 transcription reaction with all four natural rNTPs (rATP, rCTP, UTP, rGTP) with transcription using modified **rA^{CA}TP** and three natural rNTPs (rCTP, UTP, rGTP) either with or without purification.

2.6.4 Denaturing PAGE analysis of inhibition of *in vitro* transcription reaction by rA^{CA}TP

In vitro transcription reactions were performed in total volume of 10 μL in transcription reaction buffer (5X, 2 μL) containing all four natural rNTPs (2 mM, rATP, rCTP, UTP, rGTP), $MgCl_2$ (25 mM), RiboLock RNase inhibitor (1 U/μL), Triton X-100 (0.1%), dsDNA template (1.5 μM, **20DNA_1A**, prepared according to above-mentioned procedure, Section 2.6.1), T7 RNA polymerase (5 U/μL) and [α-^{32}P]-GTP (111 TBq/mmol, 370 MBq/mL, 0.1 μL) either with or without addition of **rA^{CA}TP** (2 mM). Transcription reactions were performed at 37 °C for 2 h in a thermal cycler with heated lid (75 °C). Samples were analyzed by 20% dPAGE (General procedure for dPAGE analysis, Section 2.6.2). [Gel analysis: Figure S7].

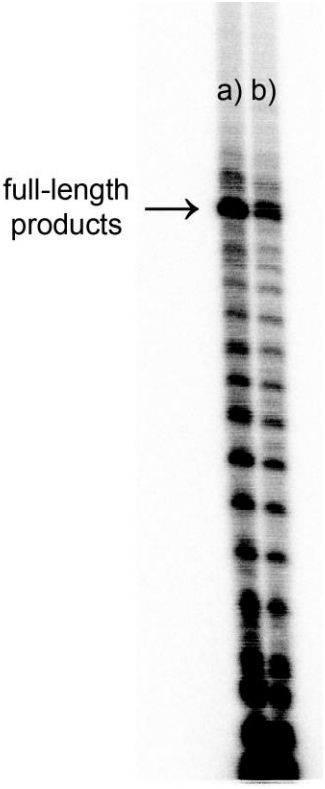

Figure S7. Phosphor imaging of 20% denaturing PAGE analysis. Comparison of T7 transcription reaction with all four natural rNTPs with transcription using all four natural rNTPs and extra addition of modified **rA^{CA}TP**. a) Transcription reaction using T7 RNA polymerase and rATP, rCTP, UTP, rGTP (2 mM) and **20DNA_1A** template. b) Transcription reaction using T7 RNA polymerase and **rA^{CA}TP**, rATP, rCTP, UTP, rGTP (2 mM) and **20DNA_1A** template.

2.6.4.1 Supplementary note no. 1 – Discussion of possible cross-linking with T7 RNAP

The crude reaction mixture after radioactive IVT with modified rA^{CA}TP and three natural rNTPs (rCTP, UTP, rGTP) was directly analyzed by denaturing SDS-PAGE analysis in comparison with IVT with natural rNTPs (rATP, rCTP, UTP, rGTP). No shifted band corresponding to the cross-linking product of modified RNA with T7 RNA polymerase was observed on SDS-PAGE. Additionally, we explored the possibility that T7 polymerase cross-links to the modified nucleotide before incorporation. This would also lead to polymerase inhibition. To elucidate this, we carried out transcription either with natural rNTPs or natural rNTPs with addition of rA^{CA}TP. The transcription yielded about the same amount of the product, suggesting that covalent inhibition by our modified nucleotide is negligible. These results strongly support our conclusions that T7 polymerase does not significantly cross-link to the modified nucleotide or RNA (although we cannot entirely rule out that traces of cross-linked conjugate are present, due to inherent detection limit of methods used). In any case, T7 RNA polymerase transcription performs well enough to enable preparation of reasonable amounts of modified RNA.

2.6.5 Incorporation of rA^{CA}TP using 20DNA_1A template in analytical scale for dPAGE analysis

In vitro transcription reactions were performed in total volume of 10 μL in transcription reaction buffer (5X, 2 μL) containing modified **rA^{CA}TP** (2 mM), three natural rNTPs (2 mM, rCTP, UTP, rGTP), $MgCl_2$ (25 mM), RiboLock RNase inhibitor (1 U/μL), Triton X-100 (0.1%), dsDNA template (1.5 μM, **20DNA_1A**, prepared according to above-mentioned procedure, Section 2.6.1), T7 RNA polymerase (5 U/μL) and [α-^{32}P]-GTP (111 TBq/mmol, 370 MBq/mL, 0.1 μL). The negative control experiment was performed under the same conditions with H_2O used instead of the solution of modified **rA^{CA}TP** and with natural rATP (2 mM) in case of positive control. Transcription reactions were performed at 37 °C for 2 h in a thermal cycler with heated lid (75 °C). Samples were analyzed by 20% dPAGE (General procedure for dPAGE analysis, Section 2.6.2). [Gel analysis: Figure S8].

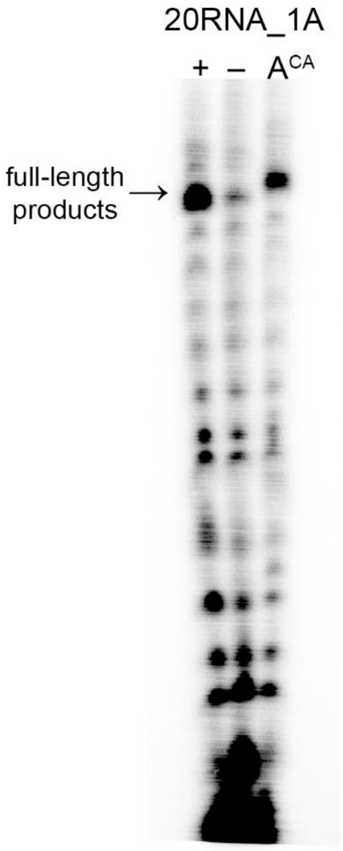

Figure S8. Phosphor imaging of 20% denaturing PAGE. Transcription reaction analysis using **20DNA_1A** template and T7 RNA polymerase. (+) positive control, all natural rNTPs (rATP, rCTP, UTP, rGTP); (–) negative control, mixture of rCTP, UTP, rGTP and H_2O; (A^{CA}) modification, mixture of rCTP, UTP, rGTP and **rA^{CA}TP**.

2.6.6 Incorporation of rA^{CA}TP using 35DNA_1A template in analytical scale for dPAGE analysis

In vitro transcription reactions were performed in total volume of 10 μL in transcription reaction buffer (5X, 2 μL) containing modified **rA^{CA}TP** (2 mM), three natural rNTPs (2 mM, rCTP, UTP, rGTP), $MgCl_2$ (15 mM), RiboLock RNase inhibitor (1 U/μL), Triton X-100

(0.1%), dsDNA template (1.25 μM, **35DNA_1A**, prepared according to above-mentioned procedure, Section 2.6.1), T7 RNA polymerase (4 U/μL) and [α-^{32}P]-GTP (111 TBq/mmol, 370 MBq/mL, 0.1 μL). The negative control experiment was performed under the same conditions with H_2O used instead of the solution of modified **rA^{CA}TP** and with natural rATP (2 mM) in case of positive control. Transcription reactions were performed at 37 °C for 2 h in a thermal cycler with heated lid (75 °C). Samples were analyzed by 12.5% dPAGE (General procedure for dPAGE analysis, Section 2.6.2). [Gel analysis: Figure S9-a) for **35DNA_1A** template].

2.6.7 Incorporation of rA^{CA}TP using 35DNA_3A and/or 35DNA_7A template in analytical scale for dPAGE analysis

In vitro transcription reactions were performed in total volume of 10 μL in transcription reaction buffer (5X, 2 μL) containing modified **rA^{CA}TP** (3 mM), three natural rNTPs (2 mM, rCTP, UTP, rGTP), $MgCl_2$ (15 mM), RiboLock RNase inhibitor (1 U/μL), Triton X-100 (0.1%), dsDNA template (1.25 μM, **35DNA_3A** or **35DNA_7A**, prepared according to above-mentioned procedure, Section 2.6.1), T7 RNA polymerase (6 U/μL) and [α-^{32}P]-GTP (111 TBq/mmol, 370 MBq/mL, 0.1 μL). The negative control experiment was performed under the same conditions with H_2O used instead of the solution of modified **rA^{CA}TP** and with natural rATP (3 mM) in case of positive control. Transcription reactions were performed at 37 °C for 2 h in a thermal cycler with heated lid (75 °C). Samples were analyzed by 12.5% dPAGE (General procedure for dPAGE analysis, Section 2.6.2). [Gel analysis: Figure S9-b) for **35DNA_3A** template; Figure S9-c) for **35DNA_7A** template].

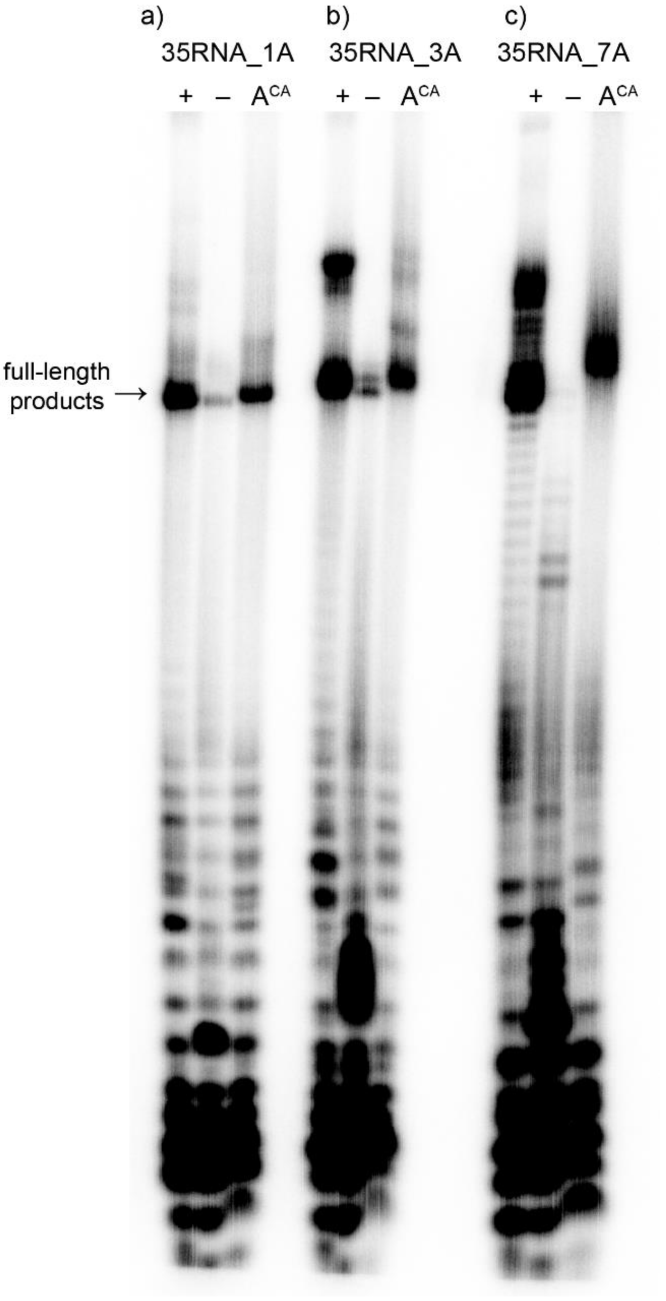

Figure S9. Phosphor imaging of 12.5% denaturing PAGE. Transcription reaction analysis using a) **35DNA_1A**, b) **35DNA_3A** or c) **35DNA_7A** template and T7 RNA polymerase. (+) positive control, all natural rNTPs (rATP, rCTP, UTP, rGTP); (–) negative control, mixture of rCTP, UTP, rGTP and H_2O; (A^{CA}) modification, mixture of rCTP, UTP, rGTP and **rA^{CA}TP**.

2.6.8 Enzymatic synthesis of 20RNA_1A or 20RNA_1A^{CA} in semi-preparative scale

In vitro transcription reactions were performed in total volume of 50 μL in transcription reaction buffer (5X, 10 μL) containing either natural rATP (2 mM) or modified **rA^{CA}TP** (2 mM), three natural rNTPs (2 mM, rCTP, UTP, rGTP), $MgCl_2$ (25 mM), RiboLock RNase inhibitor (1 U/μL), Triton X-100 (0.1%), dsDNA template (1.5 μM, **20DNA_1A**, prepared according to above-mentioned procedure, Section 2.6.1) and T7 RNA polymerase (5 U/μL). Transcription reactions were performed at 37 °C for 2 h in a thermal cycler with heated lid

(75 °C). After this time, DNase I (5 μL, 1 U/μL) was added to the mixture and the solution was further heated for 30 min at 37 °C in a thermal cycler. The samples were purified (General purification procedure I, Section 2.5.1) and either directly analyzed by MS-MALDI-TOF or evaporated to dryness and then dissolved in 10 μL of H_2O followed by mass analysis. [MS-MALDI-TOF analysis: Figure S32 for natural **20RNA_1A**, Figure S33 for modified **20RNA_1A^{CA}**].

2.6.9 Enzymatic synthesis of 35RNA_1A or 35RNA_1A^{CA} in semi-preparative scale

In vitro transcription reactions were performed in total volume of 30 μL in transcription reaction buffer (5X, 6 μL) containing either natural rATP (2 mM) or modified **rA^{CA}TP** (2 mM), three natural rNTPs (2 mM, rCTP, UTP, rGTP), $MgCl_2$ (15 mM), RiboLock RNase inhibitor (1 U/μL), Triton X-100 (0.1%), dsDNA template (1.25 μM, **35DNA_1A**, prepared according to above-mentioned procedure, Section 2.6.1) and T7 RNA polymerase (4 U/μL). Transcription reactions were performed at 37 °C for 2 h in a thermal cycler with heated lid (75 °C). After this time, DNase I (3 μL, 1 U/μL) was added to the mixture and the solution was further heated for 30 min at 37 °C in a thermal cycler. The samples were purified (General purification procedure I, Section 2.5.1) and either directly analyzed by MS-MALDI-TOF or evaporated to dryness and then dissolved in 10 μL of H_2O followed by mass analysis. [MS-MALDI-TOF analysis: Figure S36 for natural **35RNA_1A**, Figure S37 for modified **35RNA_1A^{CA}**].

2.6.10 Enzymatic synthesis of 35RNA_3A^{CA} and 35RNA_7A^{CA} in semi-preparative scale

In vitro transcription reactions were performed in total volume of 30 μL in transcription reaction buffer (5X, 6 μL) containing modified **rA^{CA}TP** (3 mM), three natural rNTPs (2 mM, rCTP, UTP, rGTP), $MgCl_2$ (15 mM), RiboLock RNase inhibitor (1 U/μL), Triton X-100 (0.1%), dsDNA template (1.25 μM, **35DNA_3A** or **35DNA_7A**, prepared according to above-mentioned procedure, Section 2.6.1) and T7 RNA polymerase (6 U/μL). Transcription reactions were performed at 37 °C for 2 h in a thermal cycler with heated lid (75 °C). After this time, DNase I (3 μL, 1 U/μL) was added to the mixture and the solution was further heated for 30 min at 37 °C in a thermal cycler. The samples were purified (General purification procedure I, Section 2.5.1) and either directly analyzed by MS-MALDI-TOF or evaporated to dryness and then dissolved in 10 μL of H_2O followed by mass analysis. [MS-MALDI-TOF analysis: Figure S38 for **35RNA_3A^{CA}**; Figure S39 for **35RNA_7A^{CA}**].

2.6.11 Enzymatic synthesis of 21RNA_3A-bind, 21RNA_3A^{CA}-bind and 21RNA_3A-non-bind, 21RNA_3A^{CA}-non-bind in preparative scale

In vitro transcription reactions were performed in total volume of 50 μL in transcription reaction buffer (5X, 10 μL) containing modified **rA^{CA}TP** (2 mM), three natural rNTPs (2 mM, rCTP, UTP, rGTP), $MgCl_2$ (25 mM), RiboLock RNase inhibitor (1 U/μL), Triton X-100 (0.1%), dsDNA template (1.5 μM, either **21DNA_3A-bind** or **21DNA_3A-non-bind**, prepared according to above-mentioned procedure, Section 2.6.1) and T7 RNA polymerase (5 U/μL). The positive control experiment was performed under the same conditions with natural rATP (2 mM) used instead of the solution of modified **rA^{CA}TP**. Transcription reactions

were performed at 37 °C for 2 h in a thermal cycler with heated lid (75 °C). After this time, DNase I (5 μL, 1 U/μL) was added to the mixture and the solution was further heated for 30 min at 37 °C in a thermal cycler. The samples were purified (General purification procedure I, Section 2.5.1) and directly analyzed by LC-MS analysis. [MS-analysis: Figures S53, S54 for **21RNA_3A-bind**; Figures S55, S56 for **21RNA_3A^{CA}-bind**; Figures S57, S58 for **21RNA_3A-non-bind**; Figures S59, S60 for **21RNA_3A^{CA}-non-bind**]. The prepared samples were further used for selective cross-linking reactions with HeLa cell lysate and western-blot analysis (Section 2.12.4).

2.6.12 Enzymatic synthesis of 20RNA_1A or 20RNA_1A^{CA} in preparative scale

In vitro transcription reactions were performed in total volume of 400 μL in transcription reaction buffer (5X, 80 μL) containing either natural rATP or modified **rA^{CA}TP** (2 mM), three natural rNTPs (2 mM, rCTP, UTP, rGTP), $MgCl_2$ (25 mM), RiboLock RNase inhibitor (1 U/μL), Triton X-100 (0.1%), dsDNA template (1.5 μM, **20DNA_1A**, prepared according to above-mentioned procedure, Section 2.6.1) and T7 RNA polymerase (5 U/μL). Transcription reactions were performed at 37 °C for 2 h in a thermal cycler with heated lid (75 °C). After this time, DNase I (80 μL, 1 U/μL) was added to the mixture and the solution was further heated for 30 min at 37 °C in a thermal cycler. The samples were purified (General purification procedure III, Section 2.5.3), [MS-MALDI-TOF analysis: Figure S34 for **20RNA_1A**; Figure S35 for **20RNA_1A^{CA}**]. [Figure S10 for gel analysis of **20RNA_1A^{CA}**]. Prepared samples were further used in ligation reaction with pCp-Cy5 (Section 2.7.2).

2.6.13 Enzymatic synthesis of 35RNA_1A or 35RNA_1A^{CA} in preparative scale

In vitro transcription reactions were performed in total volume of 400 μL in transcription reaction buffer (5X, 80 μL) containing either natural rATP or modified **rA^{CA}TP** (2 mM), three natural rNTPs (2 mM, rCTP, UTP, rGTP), $MgCl_2$ (15 mM), RiboLock RNase inhibitor (1 U/μL), Triton X-100 (0.1%), dsDNA template (1.25 μM, **35DNA_1A**, prepared according to above-mentioned procedure, Section 2.6.1) and T7 RNA polymerase (4 U/μL). Transcription reactions were performed at 37 °C for 2 h in a thermal cycler with heated lid (75 °C). After this time, DNase I (80 μL, 1 U/μL) was added to the mixture and the solution was further heated for 30 min at 37 °C in a thermal cycler. The samples were purified (General purification procedure III, Section 2.5.3). [Gel analysis: Figure S10 for **35RNA_1A^{CA}**]. Prepared samples were further used in ligation reaction with pCp-Cy5 (Section 2.7.4).

2.6.14 Enzymatic synthesis of 35RNA_3A^{CA} or 35RNA_7A^{CA} in preparative scale

In vitro transcription reactions were performed in total volume of 400 μL in transcription reaction buffer (5X, 80 μL) containing modified **rA^{CA}TP** (3 mM), three natural rNTPs (2 mM, rCTP, UTP, rGTP), $MgCl_2$ (15 mM), RiboLock RNase inhibitor (1 U/μL), Triton X-100 (0.1%), dsDNA template (1.25 μM, either **35DNA_3A** or **35DNA_7A**, prepared according to above-mentioned procedure, Section 2.6.1) and T7 RNA polymerase (6 U/μL). Transcription reactions were performed at 37 °C for 2 h in a thermal cycler with heated lid (75 °C). After this time, DNase I (80 μL, 1 U/μL) was added to the mixture and the solution was further heated for 30 min at 37 °C in a thermal cycler. The samples were purified (General purification

procedure III, Section 2.5.3). [Gel analysis: Figure S10 for **35RNA_3A^{CA}** and **35RNA_7A^{CA}**]. Prepared samples were further used in ligation reaction with pCp-Cy5 (Section 2.7.5).

2.7 RNA labelling *via* ligation reactions

2.7.1 Structures of pCp-Cy5 and pCp-Bio

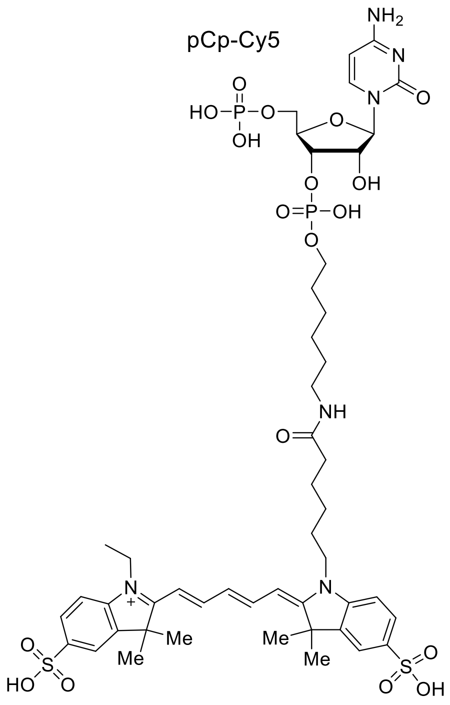

2.7.2 Preparation of 21RNA_1A-Cy5 and 21RNA_1A^{CA}-Cy5 using pCp-Cy5

The ligation reaction[7] was performed in total volume of 50 µL in T4 RNA ligase buffer (10X, 5 µL), DMSO (10%) and PEG 8000 (5%) with either natural **20RNA_1A** or modified **20RNA_1A^{CA}** (10 µM, prepared according to above-mentioned procedures: Enzymatic synthesis in preparative scale, Section 2.6.12 and General purification procedure III, Section 2.5.3), rATP (1 mM), pCp-Cy5 (50 µM) and T4 RNA ligase 1 (6 U/µL) in presence of RiboLock RNase inhibitor (1 U/µL). The mixture was incubated at 16 °C in a thermal cycler (with heated lid 65 °C) for 18 h. The mixture was freeze-dried, dissolved in 50 µL of H_2O and purified (General purification procedure I, Section 2.5.1), [MS-MALDI-TOF analysis: Figure S40 for **21RNA_1A-Cy5**; Figure S41 for **21RNA_1A^{CA}-Cy5]**, [Gel analysis: Figure S10 for **21RNA_1A^{CA}-Cy5**]. The labelled **21RNA_1A-Cy5** and **21RNA_1A^{CA}-Cy5** were after purification further used in bioconjugation reactions with (bio)molecules (Section 2.8) for dPAGE analysis, ImageJ quantification and for electrophoretic mobility shift assays (Section 2.10) or in cross-linking reactions with RBPs (Section 2.11) and Tris-Gly-SDS-PAGE analysis with ImageJ quantification.

2.7.3 Preparation of 21RNA_1A-Bio and 21RNA_1A^{CA}-Bio using pCp-Bio

The ligation reaction[8] was performed in total volume of 50 µL in T4 RNA ligase buffer (10X, 5 µL), DMSO (10%) and PEG 8000 (5%) with either natural **20RNA_1A** or modified

20RNA_1A^{CA} (10 μM, prepared according to above-mentioned procedures: Enzymatic synthesis in semi-preparative scale, Section 2.6.8 and General purification procedure I, Section 2.5.1), rATP (1 mM), pCp-Bio (50 μM) and T4 RNA ligase 1 (6 U/μL) in presence of RiboLock RNase inhibitor (1 U/μL). The mixture was incubated at 16 °C in a thermal cycler (with heated lid 65 °C) for 18 h. The mixture was freeze-dried, dissolved in 50 μL of H_2O and purified (General purification procedure I, 2.5.1), [MS-MALDI-TOF analysis: Figure S42 for **21RNA_1A-Bio**; Figure S43 for **21RNA_1A^{CA}-Bio**] The labelled **21RNA_1A-Bio** and **21RNA_1A^{CA}-Bio** were after purification further used in cross-linking reaction with hAgo2 protein for western-blot analysis (Section 2.12.3).

2.7.4 Preparation of 36RNA_1A-Cy5 and 36RNA_1A^{CA}-Cy5 using pCp-Cy5

The ligation reaction[7] was performed in total volume of 50 μL in T4 RNA ligase buffer (10X, 5 μL), DMSO (10%) and PEG 8000 (5%) with either natural **35RNA_1A** or modified **35RNA_1A^{CA}** (3 μM, prepared according to above-mentioned procedures: Enzymatic synthesis in preparative scale, Section 2.6.13 and General purification procedure III, Section 2.5.3), rATP (1 mM), pCp-Cy5 (50 μM) and T4 RNA ligase 1 (6 U/μL) in presence of RiboLock RNase inhibitor (1 U/μL). The mixture was incubated at 16 °C in a thermal cycler (with heated lid 65 °C) for 18 h. The mixture was freeze-dried, dissolved in 50 μL of H_2O and purified (General purification procedure I, Section 2.5.1), [Gel analysis: Figure S10 for **36RNA-1A^{CA}-Cy5**]. The labelled **36RNA_1A-Cy5** and **36RNA_1A^{CA}-Cy5** were after purification further used in cross-linking reactions (Section 2.11) and Tris-Gly-SDS-PAGE analysis with ImageJ quantification.

2.7.5 Preparation of 36RNA_3A^{CA}-Cy5 and 36RNA_7A^{CA}-Cy5 using pCp-Cy5

The ligation reaction[7] was performed in total volume of 50 μL in T4 RNA ligase buffer (10X, 5 μL), DMSO (10%) and PEG 8000 (5%) with either **35RNA_3A^{CA}** or **35RNA_7A^{CA}** (3 μM, prepared according to above-mentioned procedures: Enzymatic synthesis in preparative scale, Section 2.6.14 and General purification procedure III, Section 2.5.3), rATP (1 mM), pCp-Cy5 (50 μM) and T4 RNA ligase 1 (6 U/μL) in presence of RiboLock RNase inhibitor (1 U/μL). The mixture was incubated at 16 °C in a thermal cycler (with heated lid 65 °C) for 18 h. The mixture was freeze-dried, dissolved in 50 μL of H_2O and purified (General purification procedure I, Section 2.5.1), [Gel analysis: Figure S10 for **36RNA-3A^{CA}-Cy5** and **36RNA-7A^{CA}-Cy5**]. The labelled **36RNA_3A^{CA}-Cy5** and **36RNA_7A^{CA}-Cy5** were after purification further used in cross-linking reactions (Section 2.11) and Tris-Gly-SDS-PAGE analysis with ImageJ quantification.

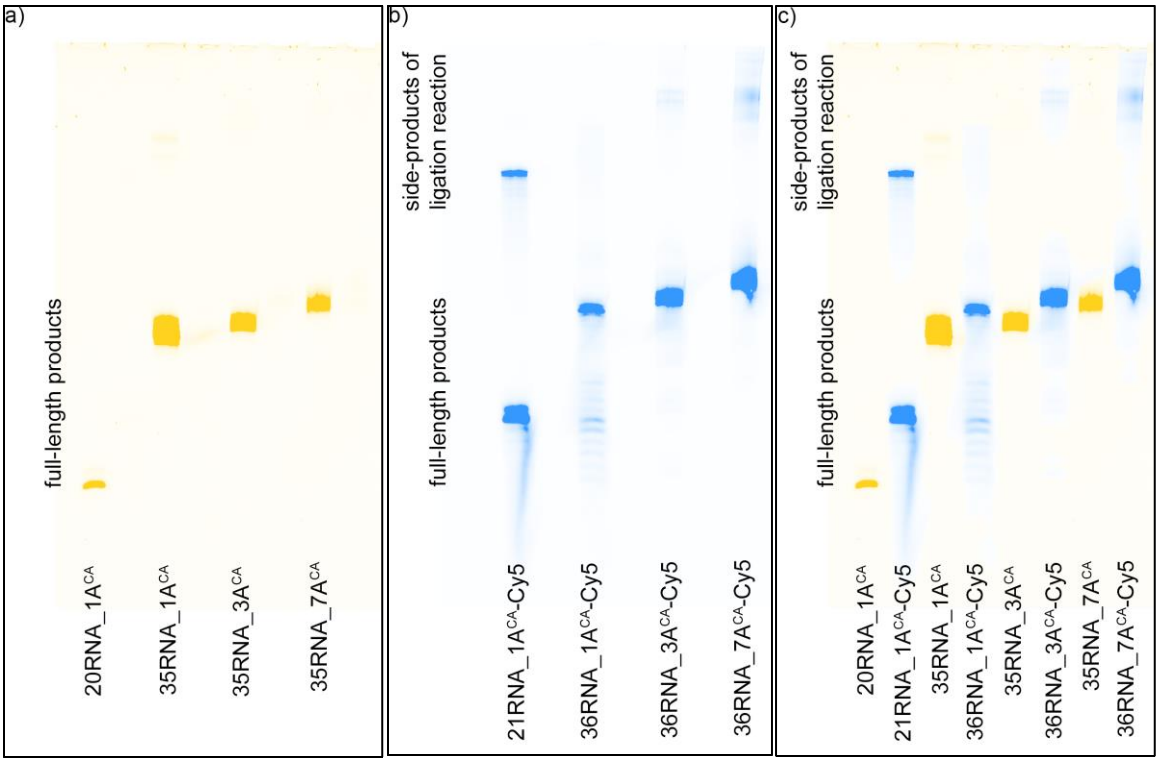

Figure S10. Denaturing PAGE analysis of ligation reaction with pCp-Cy5 of **20RNA_1A^{CA}**, **35RNA_1A^{CA}**, **35RNA_3A^{CA}** and **35RNA_7A^{CA}**. a) SybrGold channel scan after SybrGold staining of the gel. Visualization of **20RNA_1A^{CA}**, **35RNA_1A^{CA}**, **35RNA_3A^{CA}** and **35RNA_7A^{CA}** before labelling with pCp-Cy5. b) Cy5 channel scan of the gel. Visualization of **21RNA_1A^{CA}-Cy5**, **36RNA_1A^{CA}-Cy5**, **36RNA_3A^{CA}-Cy5** and **36RNA_7A^{CA}-Cy5** after labelling with pCp-Cy5. c) SybrGold and Cy5 channel scan merged.

2.8 Bioconjugation reactions, denaturing PAGE and mass analysis

2.8.1 Structures of peptides and other (bio)molecules used in this study

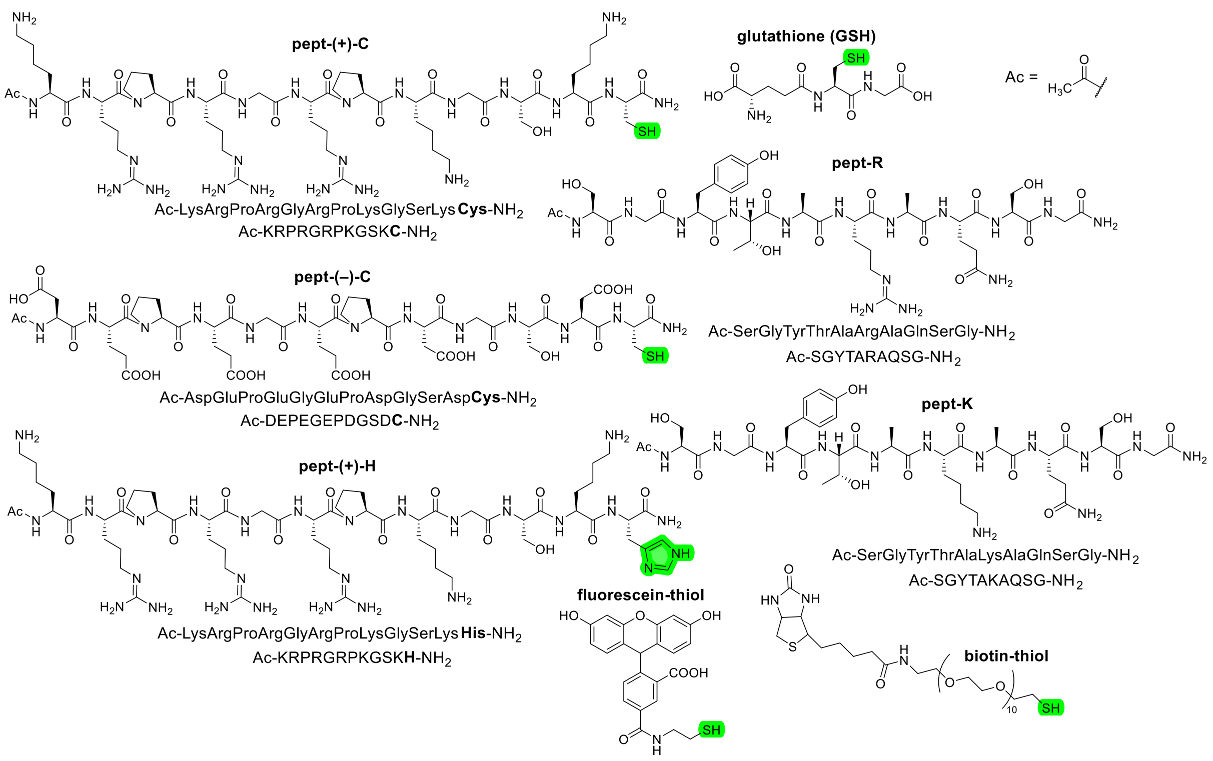

2.8.2 Bioconjugation of natural or modified RNA with either glutathione (GSH), pept-(+)-H or biotin-thiol in analytical scale for dPAGE analysis

The reaction mixture (10 µL) containing labelled RNA: **21RNA_1A-Cy5** or **21RNA_1A^{CA}-Cy5** (0.5 µM, prepared according to Ligation procedure with pCp-Cy5, Section 2.7.2) and the corresponding peptide (5 mM, 10 000 equiv. of GSH or Ac-KRPRGRPKGSKH-NH_2, pept-(+)-H, dissolved in H_2O) or biotin-thiol (5 mM, 10 000 equiv., dissolved in H_2O) was incubated in TEAA (triethylammonium acetate buffer, 0.3 M, pH = 8.0 at 25 °C) at 25 °C in a thermal cycler (with heated lid 65 °C) for 48 h. After this time, the mixture was combined with 10 µL of 2X stop solution (95% [v/v] formamide, 0.5 mM EDTA, 0.025% [w/v] bromophenol blue, 0.025% [w/v] SDS in H_2O) and denatured by heating at 95 °C for 2 min and immediately cooled on ice. Aliquots of the samples (10 µL) were subjected to vertical gel electrophoresis on 22.5% dPAGE (19:1 mono:bis acrylamide) containing 1X TBE buffer (pH = 8) and urea (7 M) at 20 mA for 2-3 h (until the dye migrated to the bottom third of the gel). The gel was visualized by a fluorescent scanner using Cy5 channel scan. [Gel analysis: Figure S11, lanes: 1, 2, 5 for bioconjugation of natural **21RNA_1A-Cy5** and Figure S12, lanes: 1, 2, 6 for bioconjugation of modified **21RNA_1A^{CA}-Cy5**].

2.8.3 Bioconjugation of natural or modified RNA with either pept-(+)-C or pept-(–)-C in analytical scale for dPAGE analysis

The reaction mixture (10 μL) containing labelled RNA: **21RNA_1A-Cy5** or **21RNA_1A^{CA}-Cy5** (0.5 μM, prepared according to Ligation procedure with pCp-Cy5, Section 2.7.2) and the corresponding peptide (either 50 μM, 100 equiv. or 1.25 mM, 2 500 equiv. of Ac-KRPRGRPKGSKC-NH_2, pept-(+)-C) or 1.25 mM, 2 500 equiv. of Ac-DEPEGEPDGSDC-NH_2, pept-(–)-C, dissolved in H_2O) was incubated in TEAA (triethylammonium acetate buffer, 0.3 M, pH = 8.0 at 25 °C) at 25 °C in a thermal cycler (with heated lid 65 °C) for 48 h. After this time, the mixture was combined with 10 μL of 2X stop solution (95% [v/v] formamide, 0.5 mM EDTA, 0.025% [w/v] bromophenol blue, 0.025% [w/v] SDS in H_2O) and denatured by heating at 95 °C for 2 min and immediately cooled on ice. Aliquots of the samples (10 μL) were then subjected to vertical gel electrophoresis on 22.5% dPAGE (19:1 mono:bis acrylamide) containing 1X TBE buffer (pH = 8) and urea (7 M) at 20 mA for 2-3 h (until the dye migrated to the bottom third of the gel). The gel was visualized by a fluorescent scanner using Cy5 channel scan. [Gel analysis: Figure S11, lines: 3, 4 for bioconjugation of natural **21RNA_1A-Cy5** and Figure S12, line: 3 for bioconjugation of modified **21RNA_1A^{CA}-Cy5** with 100 equiv. of pept-(+)-C, line: 4 for bioconjugation of modified **21RNA_1A^{CA}-Cy5** with 2 500 equiv. of pept-(+)-C, line: 5 for bioconjugation of modified **21RNA_1A^{CA}-Cy5** with 2 500 equiv. of pept-(–)-C].

2.8.4 Bioconjugation of natural or modified RNA with either pept-K or pept-R in analytical scale for dPAGE analysis

The reaction mixture (10 μL) containing labelled RNA: **21RNA_1A-Cy5** or **21RNA_1A^{CA}-Cy5** (0.5 μM, prepared according to Ligation procedure with pCp-Cy5, Section 2.7.2) and the corresponding peptide (5 mM, 10 000 equiv. of Ac-SGYTARAQSG-NH_2, pept-R or Ac-SGYTAKAQSG-NH_2, pept-K, dissolved in H_2O) was incubated in TEAA (triethylammonium acetate buffer, 0.3 M, pH = 8.0 at 25 °C) at 25 °C in a thermal cycler (with heated lid 65 °C) for 48 h. After this time, the mixture was combined with 10 μL of 2X stop solution (95% [v/v] formamide, 0.5 mM EDTA, 0.025% [w/v] bromophenol blue, 0.025% [w/v] SDS in H_2O) and denatured by heating at 95 °C for 2 min and after that immediately cooled on ice. Aliquots of the samples (10 μL) were then subjected to vertical gel electrophoresis on 22.5% dPAGE (19:1 mono:bis acrylamide) containing 1X TBE buffer (pH = 8) and urea (7 M) at 20 mA for 2-3 h (until the dye migrated to the bottom third of the gel). The gel was visualized by a fluorescent scanner using Cy5 channel scan. [Gel analysis: Figure S11, lanes: 7, 8 for bioconjugation of natural **21RNA_1A-Cy5** and Figure S12, lanes: 8, 9 for bioconjugation of modified **21RNA_1A^{CA}-Cy5**].

2.8.5 Bioconjugation of natural or modified RNA with fluorescein-thiol in analytical scale for dPAGE analysis

The reaction mixture (10 μL) containing labelled RNA: **21RNA_1A-Cy5** or **21RNA_1A^{CA}-Cy5** (0.5 μM, prepared according to Ligation procedure pCp-Cy5, Section 2.7.2) and fluorescein-thiol (5 mM, 10 000 equiv., stock in DMSO) was incubated in a solution of 5% DMSO in TEAA (triethylammonium acetate buffer, 0.3 M, pH = 8.0 at 25 °C) at 25 °C in a thermal cycler (with heated lid 65 °C) for 48 h. After this time, the mixture was combined with 10 μL of 2X stop solution (95% [v/v] formamide, 0.5 mM EDTA, 0.025% [w/v]

bromophenol blue, 0.025% [w/v] SDS in H_2O) and denatured by heating at 95 °C for 2 min and after that immediately cooled on ice. Aliquots of the samples (10 μL) were then subjected to vertical gel electrophoresis on 22.5% dPAGE (19:1 mono:bis acrylamide) containing 1X TBE buffer (pH = 8) and urea (7 M) at 20 mA for 2-3 h (until the dye migrated to the bottom third of the gel). The gel was visualized by a fluorescent scanner using Cy5 channel scan. [Gel analysis: Figure S11, lane: 6 for bioconjugation of natural **21RNA_1A-Cy5** and Figure S12, lane: 7 for bioconjugation of modified **21RNA_1A^{CA}-Cy5**].

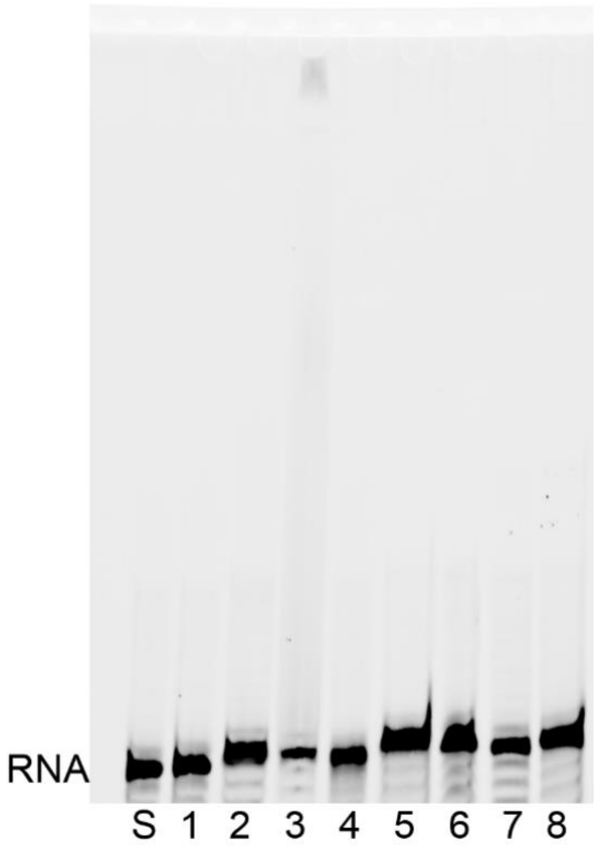

Figure S11. Cy5 channel scan of 22.5% denaturing PAGE analysis. Bioconjugation reaction of natural **21RNA_1A-Cy5** with various (bio)molecules. (S, RNA) natural RNA, standard; natural RNA in reaction with: (1) 10 000 equiv. of reduced glutathione (GSH); (2) 10 000 equiv. of pept-(+)-H; (3) 2 500 equiv. of pept-(+)-C; (4) 2 500 equiv. of pept-(–)-C; (5) 10 000 equiv. of biotin-thiol; (6) 10 000 equiv. of fluorescein-thiol; (7) 10 000 equiv. of pept-K; (8) 10 000 equiv. of pept-R.

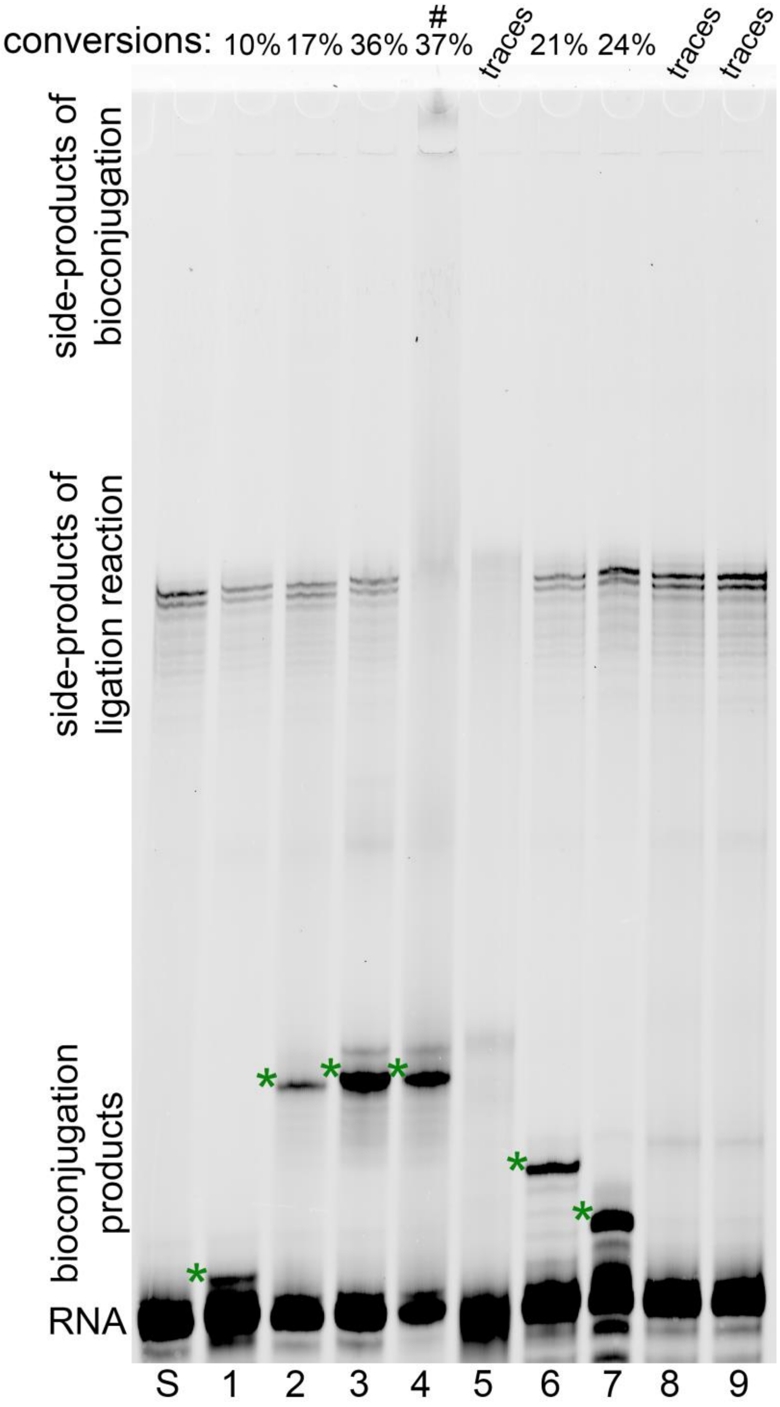

Figure S12. Cy5 channel scan of 22.5% denaturing PAGE analysis. Bioconjugation reaction of modified **21RNA_1A^{CA}-Cy5** with various (bio)molecules. (*) desired products of bioconjugation reaction; (S) modified RNA, standard; modified RNA in reaction with: (1) 10 000 equiv. of reduced glutathione (GSH); (2) 10 000 equiv. of pept-(+)-H; (3) 100 equiv. of pept-(+)-C; (4) 2 500 equiv. of pept-(+)-C, (#) significant formation of side-product affects the calculated conversion, side-product formation in 18% yield, total conversion of all products is 55%; (5) 2 500 equiv. of pept-(–)-C; (6) 10 000 equiv. of biotin-thiol; (7) 10 000 equiv. of fluorescein-thiol; (8) 10 000 equiv. of pept-K; (9) 10 000 equiv. of pept-R.

2.8.6 Bioconjugation of modified RNA with increasing concentration of pept-(+)-C in analytical scale for dPAGE analysis

The reaction mixture (10 μL) containing labelled RNA: **21RNA_1A^{CA}-Cy5** (0.5 μM, prepared according to Ligation procedure with pCp-Cy5, Section 2.7.2) and the corresponding peptide [5 μM (10 equiv.), 50 μM (100 equiv.), 250 μM (500 equiv.), 500 μM (1 000 equiv.), 1.25 mM, (2 500 equiv.), Ac-KRPRGRPKGSKC-NH_2, pept-(+)-C, dissolved in H_2O] was incubated in TEAA (triethylammonium acetate buffer, 0.3 M, pH = 8.0 at 25 °C) at 25 °C in a thermal cycler (with heated lid 65 °C) for 48 h. After this time, the mixture was combined with 10 μL of 2X stop solution (95% [v/v] formamide, 0.5 mM EDTA, 0.025% [w/v] bromophenol blue, 0.025% [w/v] SDS in H_2O) and denatured by heating at 95 °C for 2 min and immediately cooled on ice. Aliquots of the samples (10 μL) were then subjected to vertical gel electrophoresis on 22.5% dPAGE (19:1 mono:bis acrylamide) containing 1X TBE buffer (pH = 8) and urea (7 M) at 20 mA for 2-3 h (until the dye migrated to the bottom third of the gel). The gel was visualized by a fluorescent scanner using Cy5 channel scan. [Gel analysis: Figure S13].

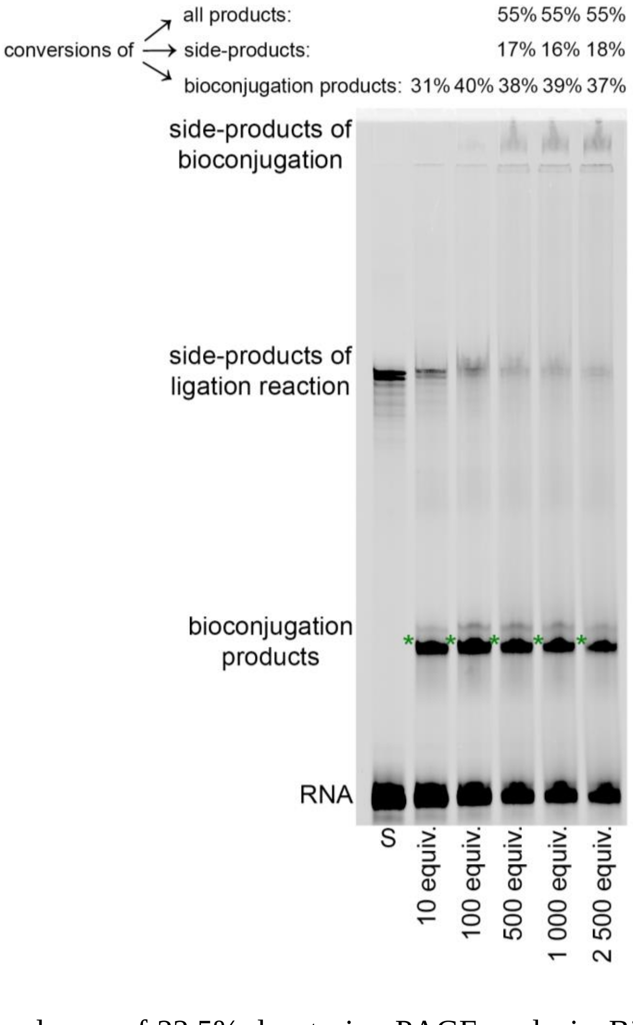

Figure S13. Cy5 channel scan of 22.5% denaturing PAGE analysis. Bioconjugation reaction of modified **21RNA_1A^{CA}-Cy5** with various concentrations of pept-(+)-C. (*) desired products of bioconjugation reaction; significant formation of side-product affects the calculated conversions in reaction with 500 equiv., 1 000 equiv. and 2 500 equiv. of pept-(+)-C; (S) modified RNA, standard.

2.8.7 Bioconjugation of modified RNA either with glutathione (GSH) or pept-(+)-H in semi-preparative scale for MS-MALDI-TOF analysis

The reaction mixture (50 μL) containing modified **20RNA_1A$^{\mathbf{CA}}$** (5 μM, prepared according to above-mentioned procedures: Enzymatic synthesis in semi-preparative scale, Section 2.6.8 and General purification procedure I, Section 2.5.1) and the corresponding peptide (50 mM, 10 000 equiv., GSH or Ac-KRPRGRPKGSKH-NH_2, pept-(+)-H, dissolved in H_2O) was incubated in TEAA (triethylammonium acetate buffer, 0.3 M, pH = 8.0 at 25 °C) at 25 °C in a thermal cycler (with heated lid 65 °C) for 48 h. The reaction mixture was purified (in case of reaction with pept-(+)-H, General purification procedure I, Section 2.5.1 was used and in case of GSH, General purification procedure II, Section 2.5.2 was used). The sample was evaporated to dryness and dissolved in 10 μL of H_2O. The sample was analyzed by MS-MALDI-TOF. [MS-MALDI-TOF analysis: Figure S44 for bioconjugation with GSH, **20RNA_1A$^{\mathbf{CA\text{-}GSH}}$**; Figure S46 for bioconjugation with pept-(+)-H, **20RNA_1A$^{\mathbf{pept\text{-}(+)\text{-}H}}$**].

2.8.8 Bioconjugation of modified RNA with pept-(+)-C in semi-preparative scale for MS-MALDI-TOF analysis

The reaction mixture (50 μL) containing modified **20RNA_1A$^{\mathbf{CA}}$** (5 μM, prepared according to above-mentioned procedures: Enzymatic synthesis in semi-preparative scale, Section 2.6.8 and General purification procedure I, Section 2.5.1) and the corresponding peptide (12.5 mM, 2 500 equiv., Ac-KRPRGRPKGSKC-NH_2, pept-(+)-C, dissolved in H_2O) was incubated in TEAA (triethylammonium acetate buffer, 0.3 M, pH = 8.0 at 25 °C) at 25 °C in a thermal cycler (with heated lid 65 °C) for 48 h. The reaction mixture was purified (General purification procedure I, Section 2.5.1). The sample was evaporated to dryness and dissolved in 10 μL of H_2O. The sample was analyzed by MS-MALDI-TOF. [MS-MALDI-TOF analysis: Figure S45, **20RNA_1A$^{\mathbf{CA\text{-}pept\text{-}(+)\text{-}C}}$**].

2.8.9 Bioconjugation of modified RNA with biotin-thiol in semi-preparative scale for MS-MALDI-TOF analysis

The reaction mixture (50 μL) containing modified **20RNA_1A$^{\mathbf{CA}}$** (5 μM, prepared according to above-mentioned procedures: Enzymatic synthesis in semi-preparative scale, Section 2.6.8 and General purification procedure I, Section 2.5.1) and biotin-thiol (50 mM, 10 000 equiv., dissolved in H_2O) was incubated in TEAA (triethylammonium acetate buffer, 0.3 M, pH = 8.0 at 25 °C) at 25 °C in a thermal cycler (with heated lid 65 °C) for 48 h. The reaction mixture was purified (General purification procedure II, Section 2.5.2). The sample was evaporated to dryness and dissolved in 10 μL of H_2O. The sample was analyzed by MS-MALDI-TOF. [MS-MALDI-TOF analysis: Figure S47, **20RNA_1A$^{\mathbf{CA\text{-}biotin}}$**].

2.8.10 Bioconjugation of modified RNA with fluorescein-thiol in semi-preparative scale for MS-MALDI-TOF analysis

The reaction mixture (10 μL) containing modified **20RNA_1A$^{\mathbf{CA}}$** (12.5 μM, prepared according to above-mentioned procedures: Enzymatic synthesis in semi-preparative scale, Section 2.6.8 and General purification procedure I, Section 2.5.1) and fluorescein-thiol (50 mM, 10 000 equiv., stock in DMSO) was incubated in a solution of 10% DMSO in TEAA (triethylammonium acetate buffer, 0.3 M, pH = 8.0 at 25 °C) at 25 °C in a thermal cycler (with

heated lid 65 °C) for 48 h. The reaction mixture was purified (General purification procedure I, Section 2.5.1). The sample was evaporated to dryness and dissolved in 10 µL of H_2O. The sample was analyzed by MS-MALDI-TOF. [MS-MALDI-TOF analysis: Figure S48, **20RNA_1A^{CA-FL}**].

2.8.11 Bioconjugation of modified RNA with pept-(+)-C in semi-preparative scale for LC-MS analysis

The reaction mixture (50 µL) containing modified **20RNA_1A^{CA}** (5 µM, prepared according to above-mentioned procedures: Enzymatic synthesis in preparative scale, Section 2.6.12 and General purification procedure III, Section 2.5.3) and the corresponding peptide (12.5 mM, 2 500 equiv., Ac-KRPRGRPKGSKC-NH_2, pept-(+)-C, dissolved in H_2O) was incubated in TEAA (triethylammonium acetate buffer, 0.3 M, pH = 8.0 at 25 °C) at 25 °C in a thermal cycler (with heated lid 65 °C) for 48 h. The reaction mixture was purified (General purification procedure I, Section 2.5.1). The sample was evaporated to dryness and dissolved in 20 µL of H_2O. 20 µL of the purified reaction mixture were injected on Waters ACQUITY Premier CSH C18 1.7 µm, 2.1 X 150 mm column [LC-MS separation chromatogram: Figure S14]. [ESI-MS spectra: Figures S49-S52]. Separation was performed at 60 °C column temperature with flow rate 0.25 mL/min with mobile phases: A (12.2 mM Et_3N, 300 mM HFIP in water) and B (12.2 mM Et_3N, 300 mM HFIP in 100% MeOH). The gradient was as follows:

**Time (min)**	**Mobile phase A (%)**	**Mobile phase B (%)**
0.00	95	5
30.00	0	100
32.00	0	100

MS settings were as follows: capillary voltage –3 kV, drying gas flow 12 L/min, nebulizer pressure 35 psig, drying gas temperature 350 °C, fragmentor 70 V, mass range 500 – 3000 m/z, 0.1 Da step size, 1.46 sec per scan cycle.

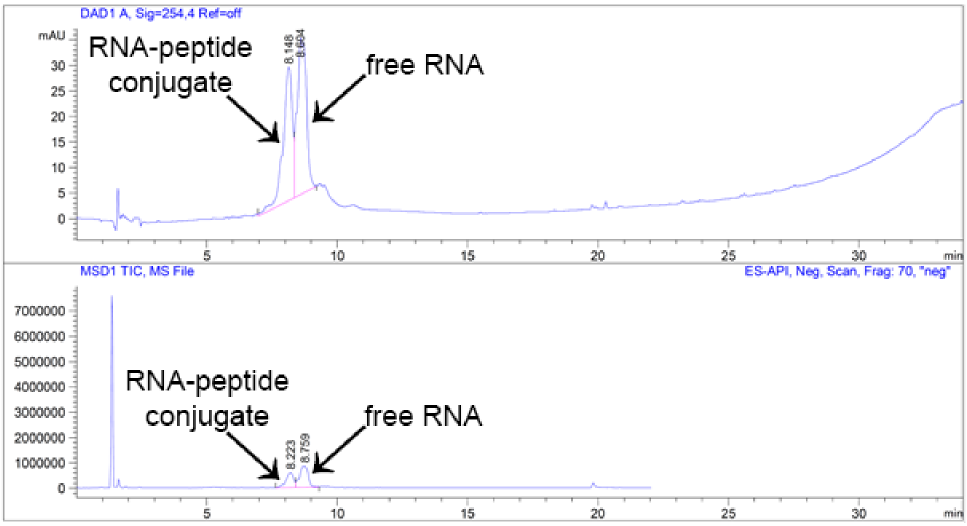

Figure S14. LC-MS separation chromatogram of purified conjugation mixture of **20RNA_1A^{CA}** and pept-(+)-C.

Quantitative data from UV (254 nm) chromatogram:

Peak	Retention time (min)	Width (min)	Area (mAU·s)	Height (mAU)	Area (%)
1 (conjugate)	8.148	0.4157	651.99976	26.13856	46.2
2 (free RNA)	8.604	0.4121	759.23291	30.70231	53.8

2.9 Fluorescence measurements

2.9.1 Bioconjugation and fluorescence measurements of either natural or modified RNA with fluorescein-thiol

The reaction mixture (10 μL) containing either natural **20RNA_1A** or modified **20RNA_1A^{CA}** (12.5 μM, prepared according to above-mentioned procedures: Enzymatic synthesis in semi-preparative scale, Section 2.6.8 and General purification procedure I, Section 2.5.1) and fluorescein-thiol (50 mM, 10 000 equiv., stock in DMSO) was incubated in a solution of 10% DMSO in TEAA (triethylammonium acetate buffer, 0.3 M, pH = 8.0 at 25 °C) at 25 °C in a thermal cycler (with heated lid 65 °C) for 48 h. The reaction mixture was purified (General purification procedure I, Section 2.5.1) and adjusted with H_2O to final volume of 100 μL and transferred to a quartz cuvette. Emission fluorescent spectra were measured at 25 °C and the excitation wavelength was set to 490 nm. [Fluorescence spectra: Figure S15 for **20RNA_1A**, **20RNA_1A^{CA}**, **20RNA_1A^{FL}**, **20RNA_1A^{CA-FL}**].

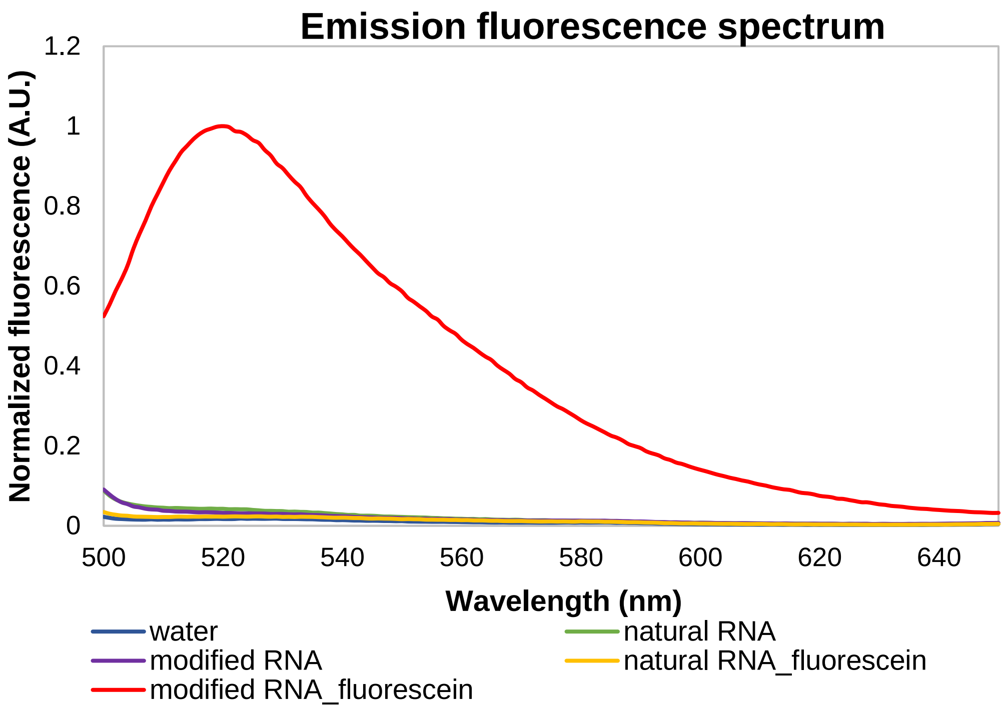

Figure S15. Normalized emission fluorescence spectra of either modified **20RNA_1A^{CA}** or natural **20RNA_1A** after bioconjugation with fluorescein-thiol.

2.10 Electrophoretic mobility shift assays (EMSA)

2.10.1 EMSA of natural or modified RNA with human antigen R (HuR)

The reaction mixture (10 μL) containing labelled RNA: **21RNA_1A-Cy5** or **21RNA_1A^{CA}-Cy5** (0.5 μM, 1 equiv., prepared according to Ligation procedure with pCp-Cy5, Section 2.7.2) in 30 mM HEPES-NaOH buffer (pH = 7.45 at 25 °C) was incubated with HuR protein (0.5 μM, 1 equiv., prepared according to above-mentioned procedure, Section 2.4.2) at 37 °C for 18 h in a thermal cycler (with heated lid at 65 °C). After this time, samples were combined with 3 μL of sterile glycerol and analyzed by 5% (0.5X TBE) native-PAGE (37.5:1 mono:bis acrylamide) in 0.5X TBE running buffer at 60 V for 2 h with cooling (4 °C). The gel was visualized by a fluorescent scanner using Cy5 channel scan. [Gel analysis: Figure S16].

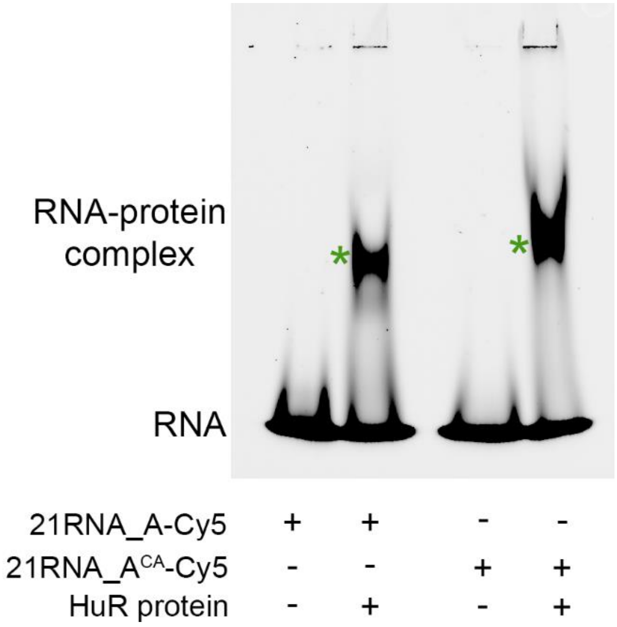

Figure S16. EMSA analysis of natural **21RNA_1A-Cy5** and modified **21RNA_1A^{CA}-Cy5** with HuR protein. Cy5 channel scan of 5% (0.5X TBE) native-PAGE. (*) RNA-protein complex.

2.10.2 EMSA of natural or modified RNA with HIV reverse transcriptase (HIV-RT)

The reaction mixture (10 μL) containing labelled RNA: **21RNA_1A-Cy5** or **21RNA_1A^{CA}-Cy5** (0.5 μM, 1 equiv., prepared according to Ligation procedure with pCp-Cy5, Section 2.7.2) in 30 mM HEPES-NaOH buffer (pH = 7.45 at 25 °C) was incubated with HIV-RT (1 μM, 2 equiv., prepared according to above-mentioned procedure, Section 2.4.1) at 20 °C for 21 h in a thermal cycler (with heated lid at 65 °C). After this time, samples were combined with 3 μL of sterile glycerol and analyzed by 5% (0.5X TBE) native-PAGE (37.5:1 mono:bis acrylamide) in 0.5X TBE running buffer at 60 V for 2 h with cooling (4 °C). The gel was visualized by a fluorescent scanner using Cy5 channel scan. [Gel analysis: Figure S17].

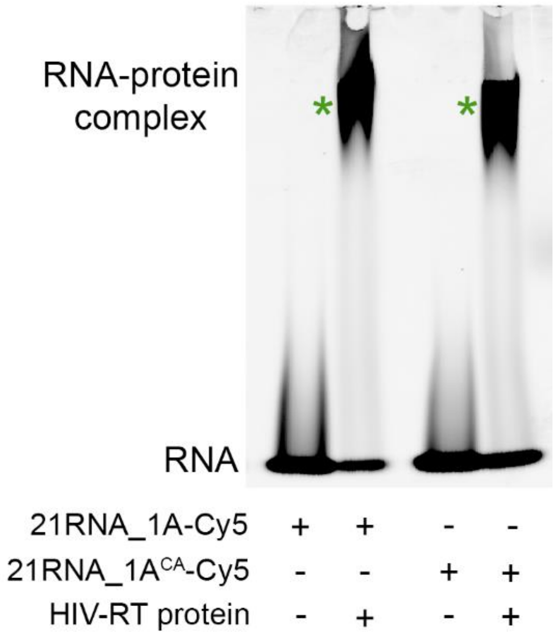

Figure S17. EMSA analysis of natural **21RNA_1A-Cy5** and modified **21RNA_1A^{CA}-Cy5** with HIV reverse transcriptase. Cy5 channel scan of 5% (0.5X TBE) native-PAGE. (*) RNA-protein complex.

2.10.3 EMSA of natural or modified RNA with human argonaute 2 protein (hAgo2)

The reaction mixture (10 μL) containing labelled RNA: **21RNA_1A-Cy5** or **21RNA_1A^{CA}-Cy5** (0.5 μM, 1 equiv., prepared according to Ligation procedure with pCp-Cy5, Section 2.7.2) in 30 mM HEPES-NaOH buffer (pH = 7.45 at 25 °C) was incubated with hAgo2 protein (1 μM, 2 equiv., Sino Biological) at 37 °C for 21 h in a thermal cycler (with heated lid at 65 °C). After this time, the samples were combined with 3 μL of sterile glycerol and analyzed by 5% (0.5X TBE) native-PAGE (37.5:1 mono:bis acrylamide) in 0.5X TBE running buffer at 60 V for 2 h with cooling (4 °C). The gel was visualized by a fluorescent scanner using Cy5 channel scan. [Gel analysis: Figure S18].

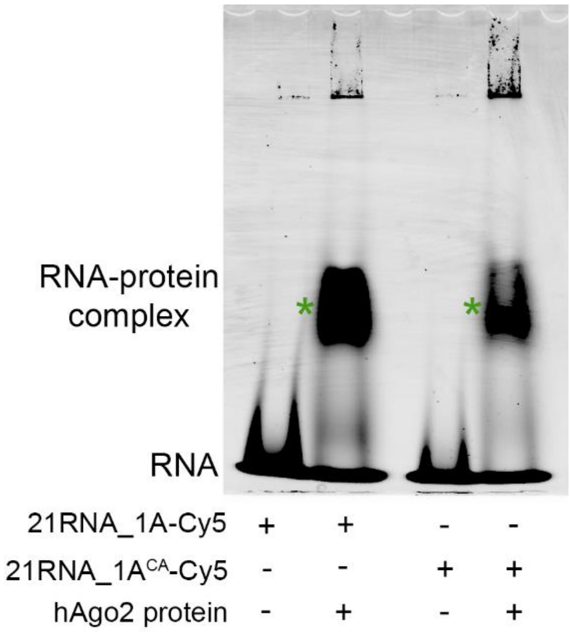

Figure S18. EMSA analysis of natural **21RNA_1A-Cy5** and modified **21RNA_1A^{CA}-Cy5** with hAgo2 protein. Cy5 channel scan of 5% (0.5X TBE) native-PAGE. (∗) RNA-protein complex.

2.11 Cross-linking reactions of RNA with proteins and SDS-PAGE analysis

2.11.1 Kinetic study of cross-linking reaction of modified RNA with HIV reverse transcriptase (HIV-RT)

The reaction mixture (10 μL) containing labelled RNA: **21RNA_1A^{CA}-Cy5** (0.5 μM, 1 equiv., prepared according to Ligation procedure with pCp-Cy5, Section 2.7.2) in 30 mM HEPES-NaOH buffer (pH = 7.45 at 25 °C) was incubated with HIV-RT (1 μM, 2 equiv., prepared according to above-mentioned procedure, Section 2.4.1) at 20 °C in a thermal cycler (with heated lid at 65 °C) for different time periods: 0.5 h, 1 h, 6 h, 10 h and 24 h. Samples were combined with 2.5 μL of 5X SDS stop solution (500 mM DTT, 150 mM Tris, 20 mM EDTA, 10% SDS, 50% glycerol, pH = 6.8) denatured at 95 °C for 5 min and analyzed by 10% denaturing Tris-Gly-SDS-PAGE (37.5:1 mono:bis acrylamide, 375 mM Tris, 0.1% SDS) in 1X Tris-Gly-SDS running buffer at 180 V for 1.5 h at room temperature. The gel was visualized by a fluorescent scanner using Cy5 channel scan. [Gel analysis: Figure S19].

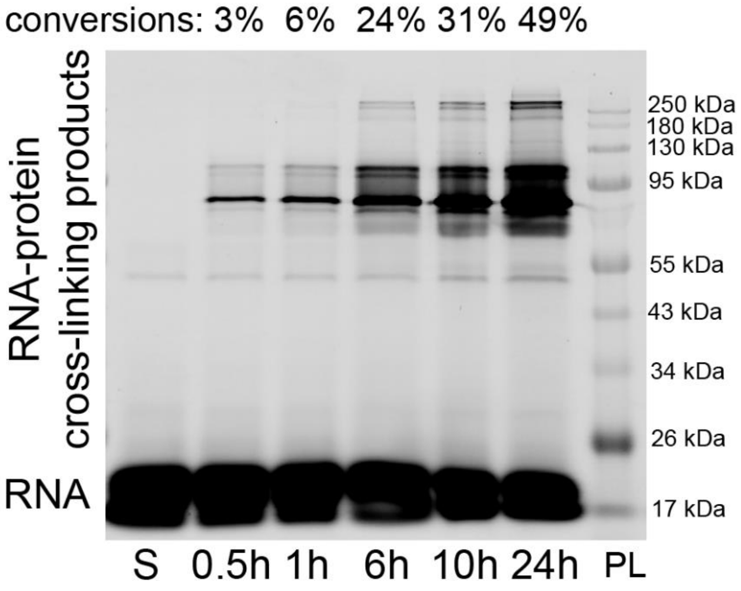

Figure S19. Analysis of cross-linking reaction of modified **21RNA_1A^{CA}-Cy5** with HIV reverse transcriptase in different time periods. Cy5 channel scan of 10% denaturing Tris-Gly-SDS-PAGE. (S; RNA) modified RNA, standard; (PL) pre-stained protein ladder.

2.11.2 Cross-linking reaction of natural or modified RNA with RNA-binding proteins (RBPs)

The reaction mixture (10 μL) containing labelled RNA: **21RNA_1A-Cy5** or **21RNA_1A^{CA}-Cy5** (0.5 μM, 1 equiv., prepared according to Ligation procedure with pCp-Cy5, Section 2.7.2) in 30 mM HEPES-NaOH buffer (pH = 7.45 at 25 °C) was incubated either with HuR protein (1 μM, 2 equiv., prepared according to above-mentioned procedure, Section 2.4.2), HIV-RT (1 μM, 2 equiv., prepared according to above-mentioned procedure, Section 2.4.1) or with hAgo2 protein (1 μM, 2 equiv., Sino Biological). Reactions with HuR and hAgo2 proteins were incubated at 37 °C and the reaction with HIV-RT was incubated at 20 °C in a thermal cycler (with heated lid at 65 °C) for 20 h. After this time, samples were combined with 2.5 μL of 5X SDS stop solution (500 mM DTT, 150 mM Tris, 20 mM EDTA, 10% SDS, 50% glycerol, pH = 6.8) denatured at 95 °C for 5 min and analyzed by 7% denaturing Tris-Gly-SDS-PAGE (37.5:1 mono:bis acrylamide, 375 mM Tris, 0.1% SDS) in 1X Tris-Gly-SDS running buffer at 180 V for 1 h at room temperature. The gel was visualized by a fluorescent scanner using Cy5 channel scan. [Gel analysis: Figure S20 for cross-linking of modified **21RNA_1A^{CA}-Cy5** and Figure S21 for cross-linking of natural **21RNA_1A-Cy5**].

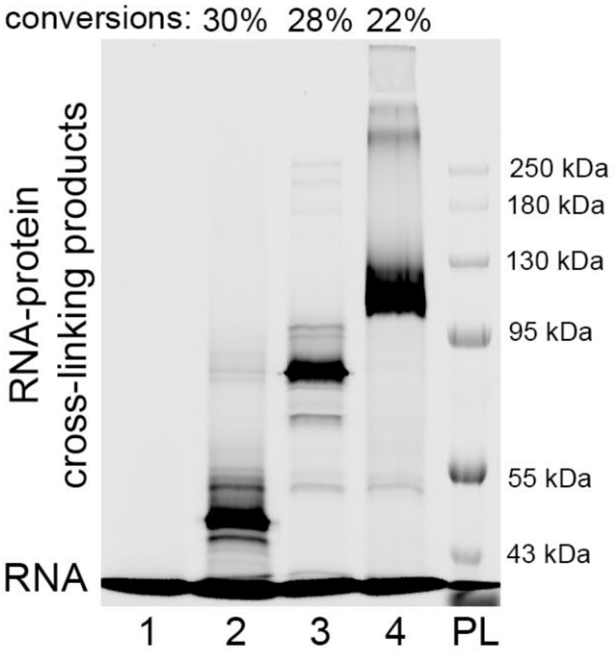

Figure S20. Analysis of cross-linking reaction of modified **21RNA_1A^{CA}-Cy5** with various RBPs. Cy5 channel scan of 7% denaturing Tris-Gly-SDS-PAGE. (RNA, 1) modified RNA, standard; modified RNA in reaction with (2) human antigen R protein (HuR); (3) HIV reverse transcriptase (HIV-RT); (4) human argonaute protein 2 (hAgo2); (PL) pre-stained protein ladder.

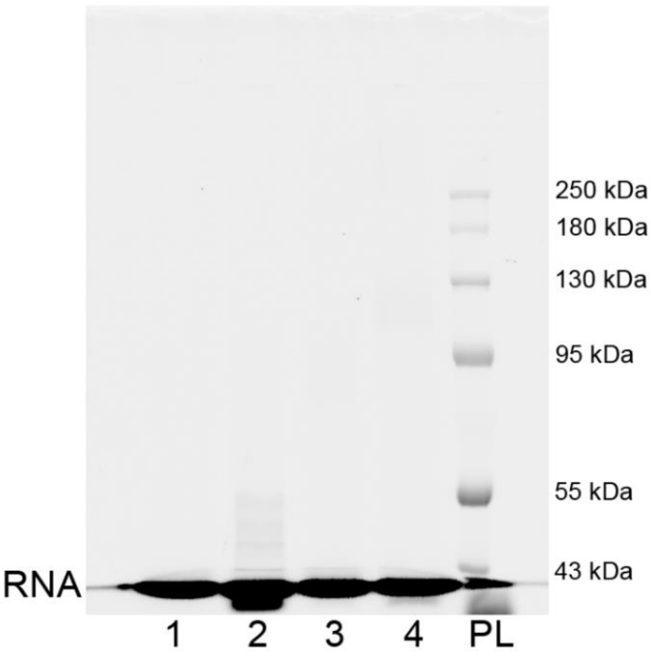

Figure S21. Analysis of cross-linking reaction of natural **21RNA_1A-Cy5** with various RBPs. Cy5 channel scan of 7% denaturing Tris-Gly-SDS-PAGE. (RNA, 1) natural RNA, standard; natural RNA in reaction with (2) human antigen R protein (HuR); (3) HIV reverse transcriptase (HIV-RT); (4) human argonaute protein 2 (hAgo2); (PL) pre-stained protein ladder.

2.11.3 Cross-linking reactions of modified RNA with HuR protein and weakly- or non-RNA-binding proteins (non-RBPs)

The reaction mixture (10 μL) containing labelled RNA: **21RNA_1A^{CA}-Cy5** (0.5 μM, 1 equiv., prepared according to Ligation procedure with pCp-Cy5, Section 2.7.2) in 30 mM HEPES-NaOH buffer (pH = 7.45 at 25 °C) was incubated either with HuR protein (1 μM, 2 equiv., prepared according to above-mentioned procedure, Section 2.4.2) or with various non-RBPs: BSA (1 μM, 2 equiv., Merck), SSB (1 μM, 2 equiv., Merck), Gal1 (1 μM, 2 equiv., prepared according to above-mentioned procedure, Section 2.4.3), lysozyme (1 μM, 2 equiv., Merck) and histone H2A (1 μM, 2 equiv., New England Biolabs) at 37 °C for 18 h in a thermal cycler (with heated lid at 65 °C). After this time, the samples were combined with 2.5 μL of 5X SDS stop solution (500 mM DTT, 150 mM Tris, 20 mM EDTA, 10% SDS, 50% glycerol, pH = 6.8) denatured at 95 °C for 5 min and analyzed by 15% denaturing Tris-Gly-SDS-PAGE (37.5:1 mono:bis acrylamide, 375 mM Tris, 0.1% SDS) in 1X Tris-Gly-SDS running buffer at 180 V for 2 h at room temperature. The gel was visualized by a fluorescent scanner using Cy5 channel scan. [Gel analysis: Figure S22].

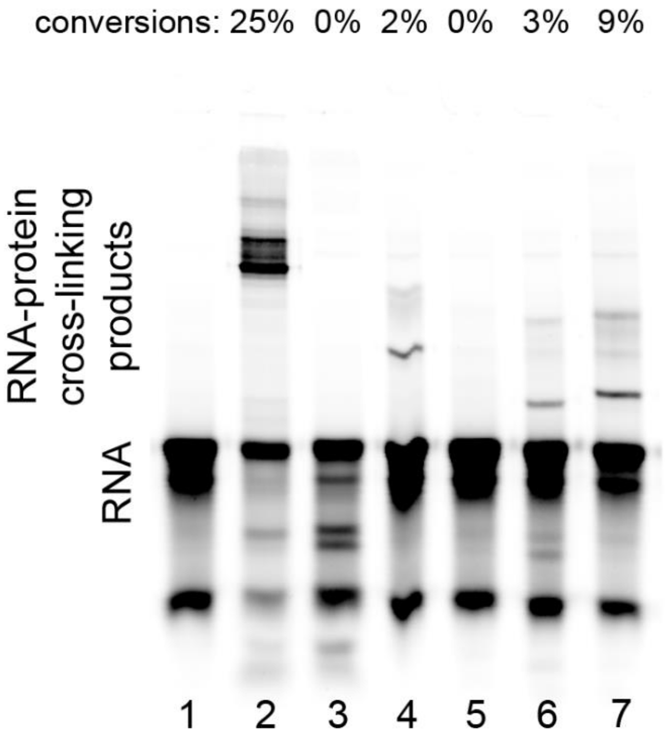

Figure S22. Analysis of cross-linking reaction of modified **21RNA_1A^{CA}-Cy5** with various proteins. Cy5 channel scan of 15% denaturing Tris-Gly-SDS-PAGE. (RNA, 1) modified RNA, standard; modified RNA in reaction with: (2) human antigen R (HuR); (3) bovine serum albumin (BSA); (4) single strand binding protein (SSB); (5) galectin 1 (Gal1); (6) lysozyme (lysoz.); (7) human recombinant histone H2A (H2A).

2.11.4 Cross-linking reactions of either natural 36RNA_1A-Cy5 or modified 36RNA-1A^{CA}-Cy5, 36RNA-3A^{CA}-Cy5 and 36RNA-7A^{CA}-Cy5 with HuR protein

The reaction mixture (10 μL) containing labelled RNA: **36RNA_1A-Cy5** or **36RNA_1A^{CA}-Cy5**, **36RNA_3A^{CA}-Cy5**, **36RNA_7A^{CA}-Cy5** (0.5 μM, 1 equiv., prepared according to Ligation procedure with pCp-Cy5, Section 2.7.4 and Section 2.7.5) in 30 mM HEPES-NaOH buffer (pH = 7.45 at 25 °C) in presence of RiboLock RNase inhibitor (1 μL, 40 U/μL) was incubated with HuR protein (1 μM, 2 equiv., prepared according to above-mentioned procedure, Section 2.4.2) at 37 °C for 15 h in a thermal cycler (with heated lid at 65 °C). After this time, the samples were combined with 2.5 μL of 5X SDS stop solution (500 mM DTT, 150 mM Tris, 20 mM EDTA, 10% SDS, 50% glycerol, pH = 6.8) denatured at 95 °C for 5 min and analyzed by 7% denaturing Tris-Gly-SDS-PAGE (37.5:1 mono:bis acrylamide, 375 mM Tris, 0.1% SDS) in 1X Tris-Gly-SDS running buffer at 180 V for 1 h at room temperature. The gel was visualized by a fluorescent scanner using Cy5 channel scan. [Gel analysis: Figure S23].

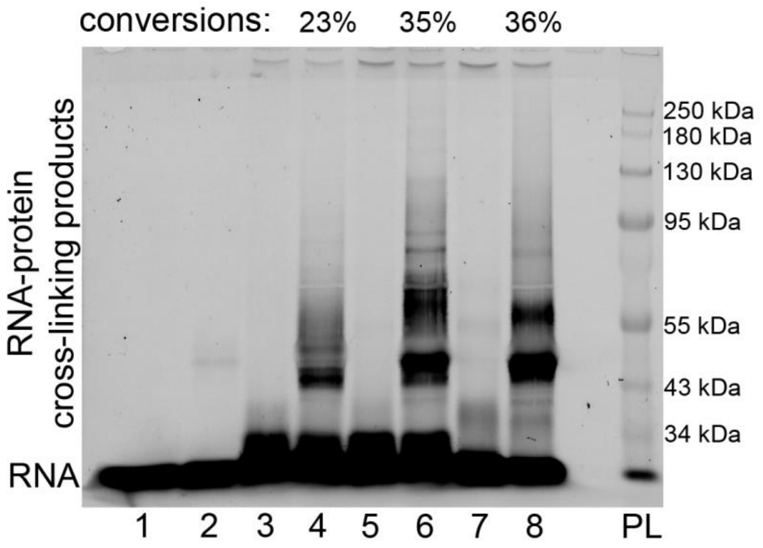

Figure S23. Analysis of cross-linking reaction of either natural **36RNA_1A-Cy5** or various modified RNA probes: **36RNA_1A^{CA}-Cy5**, **36RNA_3A^{CA}-Cy5**, **36RNA_7A^{CA}-Cy5** with HuR protein. Cy5 channel scan of 7% denaturing Tris-Gly-SDS-PAGE. (1) natural **36RNA_1A-Cy5**, standard; (2) natural **36RNA_1A-Cy5** with HuR protein; (3) modified **36RNA_1A^{CA}-Cy5**, standard; (4) modified **36RNA_1A^{CA}-Cy5** with HuR protein; (5) modified **36RNA_3A^{CA}-Cy5**, standard; (6) modified **36RNA_3A^{CA}-Cy5** with HuR protein; (7) modified **36RNA_7A^{CA}-Cy5**, standard; (8) modified **36RNA_7A^{CA}-Cy5** with HuR protein; (PL) pre-stained protein ladder.

2.11.5 Cross-linking reactions of either natural 36RNA_1A-Cy5 or modified 36RNA-1A^{CA}-Cy5, 36RNA-3A^{CA}-Cy5 and 36RNA-7A^{CA}-Cy5 with bovine serum albumin

The reaction mixture (10 μL) containing labelled RNA: **36RNA_1A-Cy5** or **36RNA_1A^{CA}-Cy5**, **36RNA_3A^{CA}-Cy5**, **36RNA_7A^{CA}-Cy5** (0.5 μM, 1 equiv., prepared according to Ligation procedure with pCp-Cy5, Section 2.7.4 and Section 2.7.5) in 30 mM HEPES-NaOH buffer (pH = 7.45 at 25 °C) in presence of RiboLock RNase inhibitor (1 μL, 40 U/μL) was

incubated with BSA (1 μM, 2 equiv., Merck) at 37 °C for 17 h in a thermal cycler (with heated lid at 65 °C). After this time, the samples were combined with 2.5 μL of 5X SDS stop solution (500 mM DTT, 150 mM Tris, 20 mM EDTA, 10% SDS, 50% glycerol, pH = 6.8) denatured at 95 °C for 5 min and analyzed by 7% denaturing Tris-Gly-SDS-PAGE (37.5:1 mono:bis acrylamide, 375 mM Tris, 0.1% SDS) in 1X Tris-Gly-SDS running buffer at 180 V for 1 h at room temperature. The gel was visualized by a fluorescent scanner using Cy5 channel scan. [Gel analysis: Figure S24].

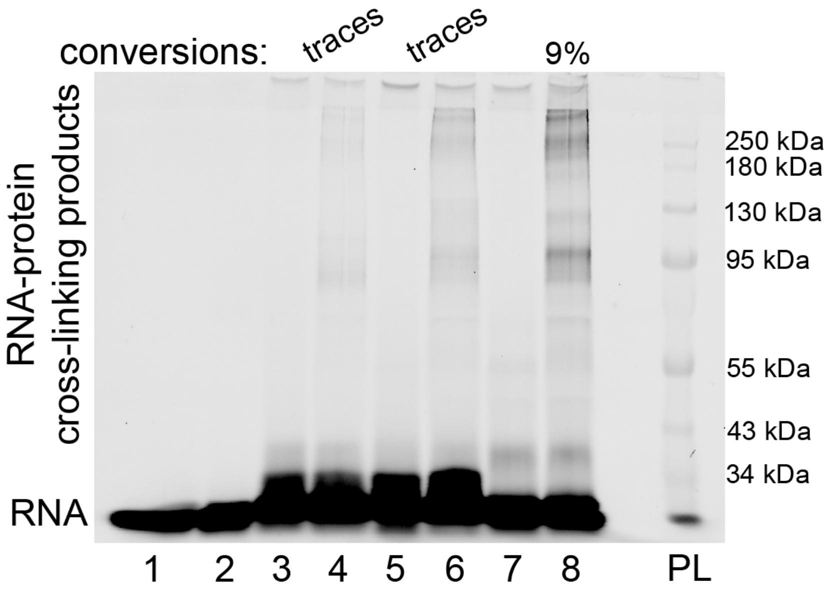

Figure S24. Analysis of cross-linking reaction of either natural **36RNA_1A-Cy5** or various modified RNA probes: **36RNA_1A^{CA}-Cy5**, **36RNA_3A^{CA}-Cy5**, **36RNA_7A^{CA}-Cy5** with BSA. Cy5 channel scan of 7% denaturing Tris-Gly-SDS-PAGE. (1) natural **36RNA_1A-Cy5**, standard; (2) natural **36RNA_1A-Cy5** with BSA; (3) modified **36RNA_1A^{CA}-Cy5**, standard; (4) modified **36RNA_1A^{CA}-Cy5** with BSA; (5) modified **36RNA_3A^{CA}-Cy5**, standard; (6) modified **36RNA_3A^{CA}-Cy5** with BSA; (7) modified **36RNA_7A^{CA}-Cy5**, standard; (8) modified **36RNA_7A^{CA}-Cy5** with BSA; (PL) pre-stained protein ladder.

2.11.6 Cross-linking reactions of either natural 36RNA_1A-Cy5 or modified 36RNA-1A^{CA}-Cy5 with HeLa cell lysate proteins

The reaction mixture (10 μL) containing labelled RNA: **36RNA_1A-Cy5** or **36RNA_1A^{CA}-Cy5** (0.5 μM, 1 equiv., prepared according to Ligation procedure with pCp-Cy5, Section 2.7.4) in 30 mM HEPES-NaOH buffer (pH = 7.45 at 25 °C) in presence of RiboLock RNase inhibitor (1 μL, 40 U/μL) was incubated with HeLa cell lysate (5 μL, 2.72 μg/μL; prepared according to above-mentioned procedure, Section 2.4.4) at 37 °C for 16 h in a thermal cycler (with heated lid at 65 °C). After this time, the samples were combined with 2.5 μL of 5X SDS stop solution (500 mM DTT, 150 mM Tris, 20 mM EDTA, 10% SDS, 50% glycerol, pH = 6.8) and dextran sulfate (1 μL, 50 mM) and denatured at 95 °C for 5 min and analyzed by 7% denaturing Tris-Gly-SDS-PAGE (37.5:1 mono:bis acrylamide, 375 mM Tris, 0.1% SDS) in 1X Tris-Gly-SDS running buffer at 180 V for 1 h at room temperature. The gel was visualized by a fluorescent scanner using Cy5 channel scan. [Gel analysis: Figure S25].

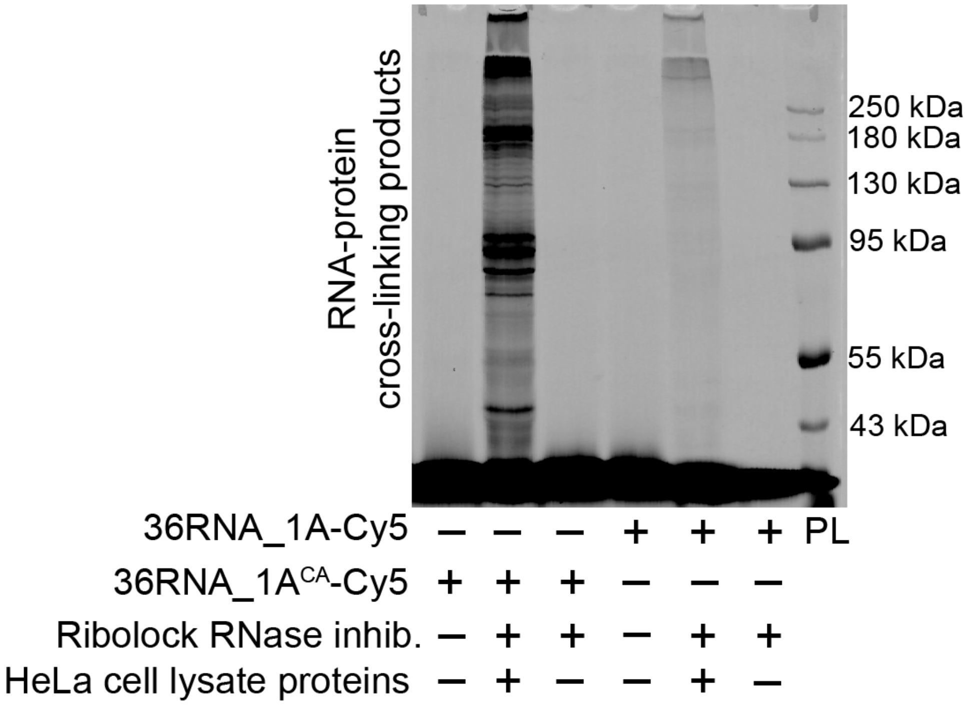

Figure S25. Analysis of cross-linking reaction of either natural **36RNA_1A-Cy5** or modified **36RNA_1A^{CA}-Cy5** with HeLa cell lysate proteins. Cy5 channel scan of 7% denaturing Tris-Gly-SDS-PAGE. (PL) pre-stained protein ladder.

2.12 Western-blot (WB) analysis of cross-linking reactions

2.12.1 WB analysis of cross-linking reaction of natural or modified RNA with HuR protein

The reaction mixture (10 μL) containing **20RNA_1A** or **20RNA_1A^{CA}** (3 μM, 1.5 equiv., prepared according to above-mentioned procedures: Enzymatic synthesis in semi-preparative scale, Section 2.6.8 and General purification procedure I, Section 2.5.1) in 30 mM HEPES-NaOH buffer (pH = 7.45 at 25 °C) was incubated with HuR protein (2 μM, 1 equiv., prepared according to above-mentioned procedure, Section 2.4.2) at 37 °C for 21 h in a thermal cycler (with heated lid at 65 °C). The samples were combined with 2.5 μL of 5X SDS stop solution (500 mM DTT, 150 mM Tris, 20 mM EDTA, 10% SDS, 50% glycerol, pH = 6.8) denatured at 95 °C for 5 min and analyzed by 10% denaturing Tris-Gly-SDS-PAGE (37.5:1 mono:bis acrylamide, 375 mM Tris, 0.1% SDS) in 1X Tris-Gly-SDS running buffer at 150 V for 1.5 h at room temperature. The WB was performed using the WET/TANK blotting system (Mini Trans-Blot® Cell, from Bio-Rad) and PVDF transfer membrane (Sigma Aldrich). After protein transfer, the membrane was incubated in blocking buffer (5.5% casein buffer, from SDT GmbH) at room temperature for 1 h and then with the fluorescent (Alexa Fluor 488) mouse monoclonal IgG_1 anti-HuR/ELAVL1 antibody (1:200 diluted, Santa Cruz Biotechnology) and subsequently with fluorescent secondary antibody Alexa Fluor 488 goat anti-mouse IgG (H+L) cross-adsorbed secondary antibody (1:400 diluted, Thermo Fisher Scientific) overnight at 4 °C. The membrane was visualized by a fluorescent scanner using Alexa Fluor 488 channel scan. [Gel analysis: Figure S26].

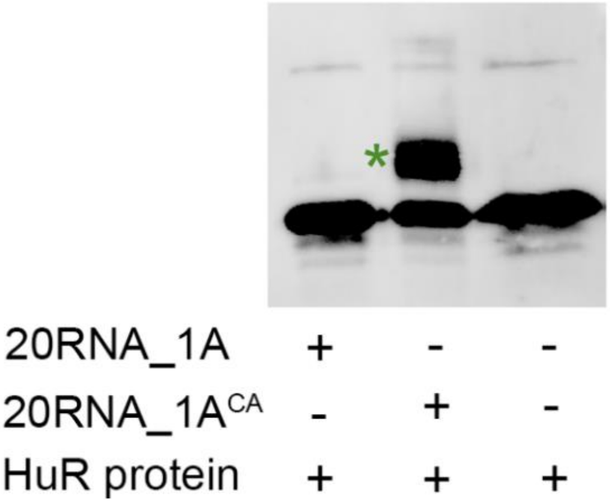

Figure S26. Analysis of cross-linking reaction of either modified **20RNA_1A^{CA}** or natural **20RNA_1A** with human antigen R protein (HuR) by western blot. Alexa Fluor 488 channel scan of the membrane. (∗) RNA-protein cross-link.

2.12.2 WB analysis of cross-linking reaction of natural or modified RNA with HIV-RT

The reaction mixture (20 μL) containing **20RNA_1A** or **20RNA_1A^{CA}** (3 μM, 1.5 equiv., prepared according to above-mentioned procedures: Enzymatic synthesis in semi-preparative scale, Section 2.6.8 and General purification procedure I, Section 2.5.1) in 30 mM HEPES-NaOH buffer (pH = 7.45 at 25 °C) was incubated with HIV-RT (2 μM, 1 equiv., prepared according to above-mentioned procedure, Section 2.4.1) at 20 °C for 20 h in a thermal cycler (with heated lid at 65 °C). The samples were combined with 5 μL of 5X SDS stop solution (500 mM DTT, 150 mM Tris, 20 mM EDTA, 10% SDS, 50% glycerol, pH = 6.8) denatured at 95 °C for 5 min and analyzed by 10% denaturing Tris-Gly-SDS-PAGE (37.5:1 mono:bis acrylamide, 375 mM Tris, 0.1% SDS) in 1X Tris-Gly-SDS running buffer at 150 V for 1.5 h at room temperature. The WB was performed using WET/TANK blotting system (Mini Trans-Blot® Cell, from Bio-Rad) and PVDF transfer membrane (Sigma Aldrich). After protein transfer, the membrane was incubated in blocking buffer (5.5% casein buffer, from SDT GmbH) at room temperature for 1 h and then with rabbit anti-HIV 1 reverse transcriptase polyclonal antibody (1:500 diluted, Thermo Fisher Scientific) and subsequently with fluorescent secondary antibody Alexa Fluor 488 affinity pure goat anti-rabbit IgG (1:500 diluted, Jackson ImmunoResearch) overnight at 4 °C. The membrane was visualized by a fluorescent scanner using Alexa Fluor 488 channel scan. [Gel analysis: Figure S27].

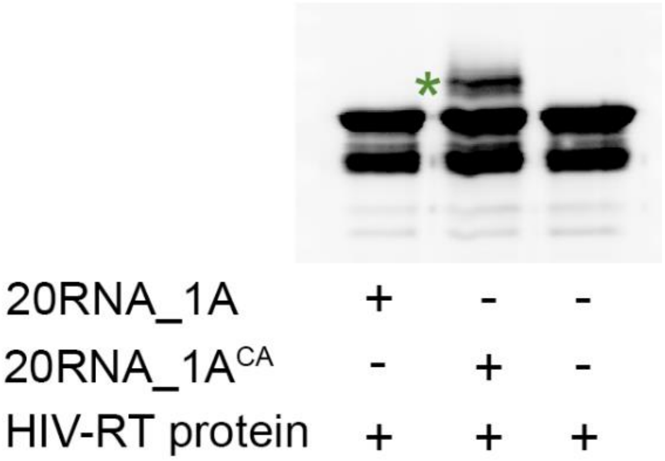

Figure S27. Analysis of cross-linking reaction of either modified **20RNA_1A^{CA}** or natural **20RNA_1A** with HIV reverse transcriptase (HIV-RT) by western blot. Alexa Fluor 488 channel scan of the membrane. (*) RNA-protein cross-link.

2.12.3 WB analysis of cross-linking reaction of biotinylated natural or modified RNA with hAgo2 protein

The reaction mixture (10 μL) containing **21RNA_1A-Bio** or **21RNA_1A^{CA}-Bio** (7 μM, 3.5 equiv., prepared according to Ligation procedure with pCp-Bio, Section 2.7.3) in 30 mM HEPES-NaOH buffer (pH = 7.45 at 25 °C) was incubated with hAgo2 protein (2 μM, 1 equiv., Sino Biological) at 37 °C for 19 h in a thermal cycler (with heated lid at 65 °C). The samples were combined with 2.5 μL of 5X SDS stop solution (500 mM DTT, 150 mM Tris, 20 mM EDTA, 10% SDS, 50% glycerol, pH = 6.8) denatured at 95 °C for 5 min and analyzed by 7% denaturing Tris-Gly-SDS-PAGE (37.5:1 mono:bis acrylamide, 375 mM Tris, 0.1% SDS) in 1X Tris-Gly-SDS running buffer at 180 V for 1 h at room temperature. The WB was performed using WET/TANK blotting system (Mini Trans-Blot® Cell, from Bio-Rad) and PVDF transfer membrane (Sigma Aldrich). After protein transfer, the membrane was incubated in blocking buffer (5.5% casein buffer, from SDT GmbH) at room temperature for 1 h and then with rabbit anti-Argonaute-2 polyclonal antibody (1:500 diluted, Sino Biological), following by fluorescent secondary antibody Alexa Fluor 488 affinity pure goat anti-rabbit IgG (1:500 diluted, Jackson ImmunoResearch) and subsequently with eBioscience Streptavidin APC conjugate (1:500 diluted, Thermo Fisher Scientific) overnight at 4 °C. The membrane was visualized by a fluorescent scanner using Alexa Fluor 488 channel and Cy5 channel scans. [Gel analysis: Figure S28].

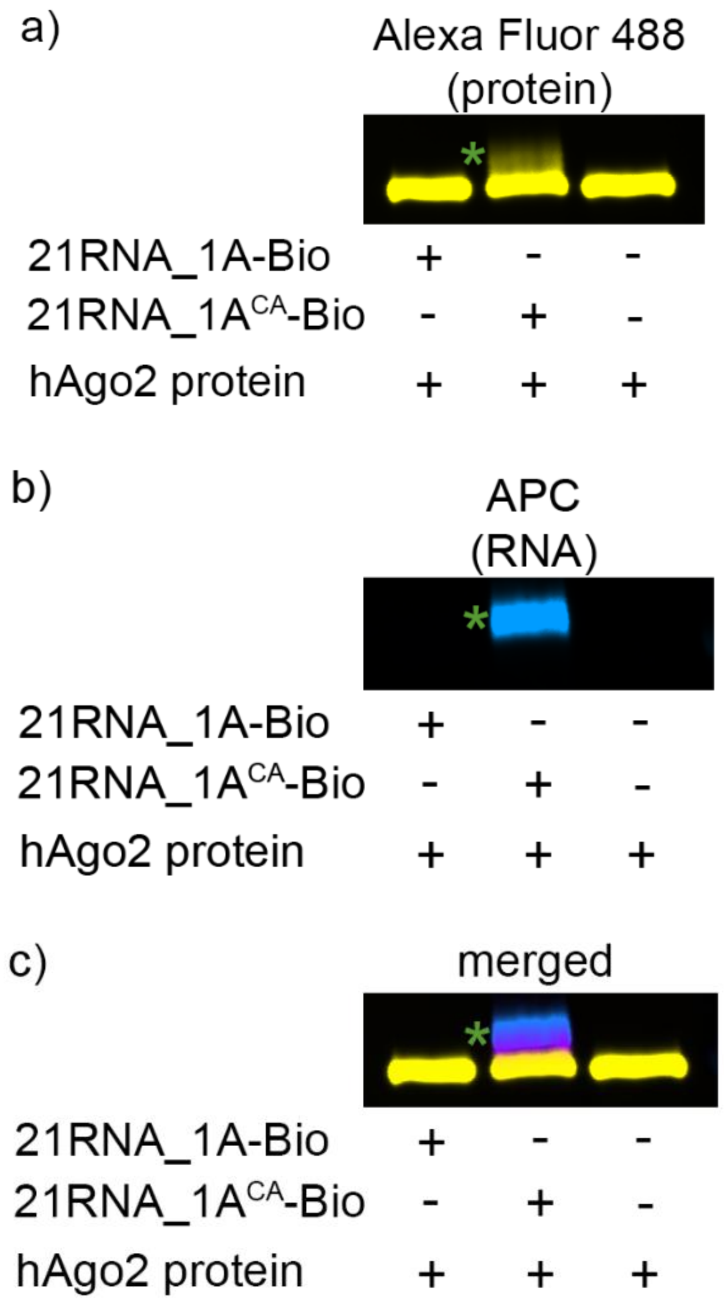

Figure S28. Analysis of cross-linking reaction of either modified **21RNA_1A^{CA}-Bio** or natural **21RNA_1A-Bio** with human argonaute protein 2 (hAgo2) by western blot. a) Alexa Fluor 488 channel scan of the membrane, visualization of the protein; b) APC (Cy5) channel scan of the membrane, visualization of cross-linked modified **21RNA_1A^{CA}-Bio**; c) merged, Alexa Fluor 488 channel and APC (Cy5) channel scans. (*) RNA-protein cross-link.

2.12.4 Selective targeting of HuR protein by cross-linking reaction in HeLa cell lysate

The reaction mixture (20 μL) containing either binding sequences for HuR: natural **21RNA_3A-bind** or modified **21RNA_3A^{CA}-bind** or non-binding sequences for HuR: natural **21RNA_3A-non-bind** or modified **21RNA_3A^{CA}-non-bind** (20 μM, prepared according to above-mentioned procedures: Enzymatic synthesis in semi-preparative scale, Section 2.6.11 and General purification procedure I, Section 2.5.1) was incubated with HeLa cell lysate

(9.5 μL, 15 μg/μL, prepared according to above-mentioned procedure, Section 2.4.4) in presence of RiboLock RNase inhibitor (0.5 μL, 40 U/μL) at 37 °C for 16 h in a thermal cycler (with heated lid at 65 °C). After this time, the samples were combined with 5 μL of 5X SDS stop solution (500 mM DTT, 150 mM Tris, 20 mM EDTA, 10% SDS, 50% glycerol, pH = 6.8) and denatured at 95 °C for 5 min and analyzed by 7% denaturing Tris-Gly-SDS-PAGE (37.5:1 mono:bis acrylamide, 375 mM Tris, 0.1% SDS) in 1X Tris-Gly-SDS running buffer at 180 V for 45 min at room temperature. The WB was performed using WET/TANK blotting system (Mini Trans-Blot® Cell, from Bio-Rad) and PVDF transfer membrane (Sigma Aldrich). After protein transfer, the membrane was incubated in blocking buffer (5.5% casein buffer, from SDT GmbH) for 48 h at 4 °C and then with the fluorescent (Alexa Fluor 488) mouse monoclonal IgG_1 anti-HuR/ELAVL1 antibody (1:200 diluted, Santa Cruz Biotechnology) for 2.5 h at room temperature and subsequently with horse anti-mouse IgG, HRP-linked secondary antibody (1:2 500 diluted, Cell Signaling Technology) for 2 h at room temperature. The membrane was incubated with chemiluminescent substrate Radiance ECL (Azure biosystems) for 2 min. The membrane was visualized by chemiluminescence blot imaging by a chemiluminescent scanner Syngene G:BOX Chemi XRQ (UK). [Gel analysis: Figure S29].

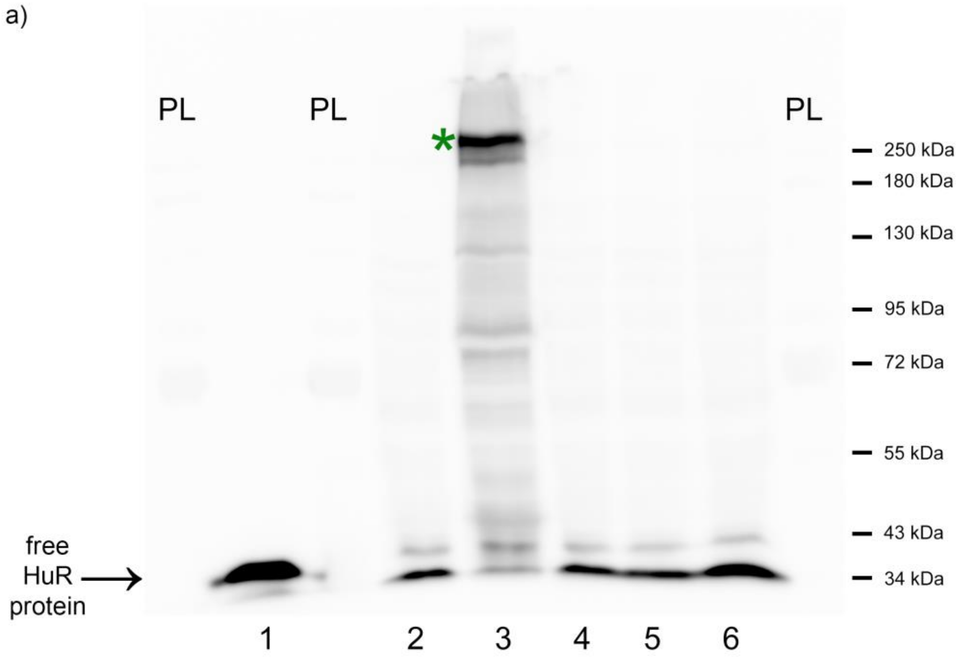

b)

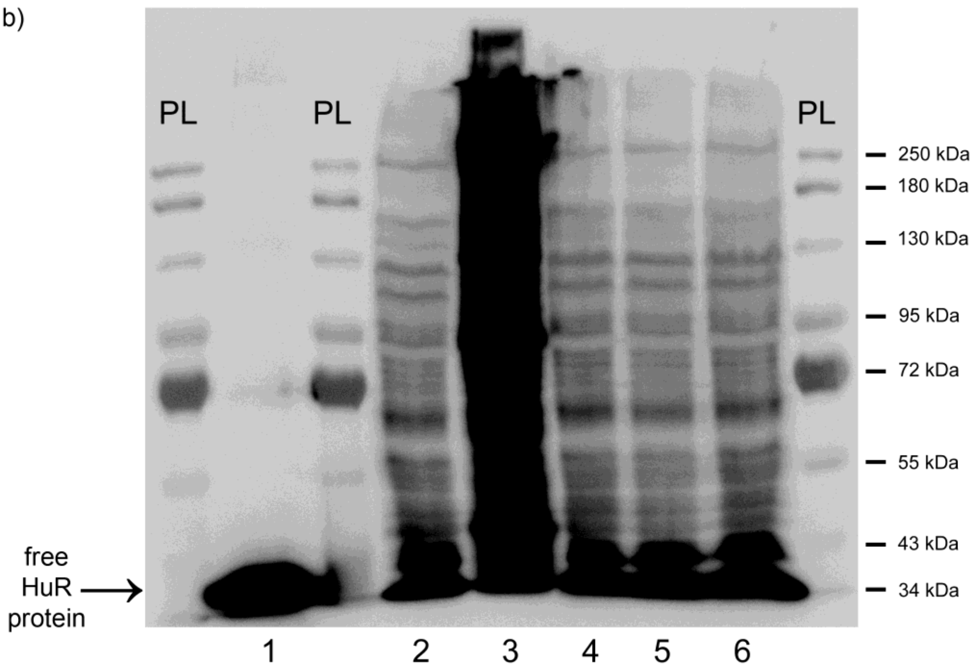

Figure S29. Analysis of selective cross-linking reaction with HuR protein in HeLa cell lysate by western-blot. Analysis by chemiluminescence blot imaging. (1) HuR protein (0.5 μM), standard; (2) **21RNA_3A-bind** with HeLa cell lysate; (3) **21RNA_3A^{CA}-bind** with HeLa cell lysate, formation of HuR-RNA oligomers (Figure S30); (4) HeLa cell lysate, standard; (5) **21RNA_3A-non-bind** with HeLa cell lysate; (6) **21RNA_3A^{CA}-non-bind** with HeLa cell lysate; (PL) pre-stained protein ladder. Figure S29 a) short exposition; Figure S29 b) long exposition. (*) RNA-protein cross-link in Figure S29 a).

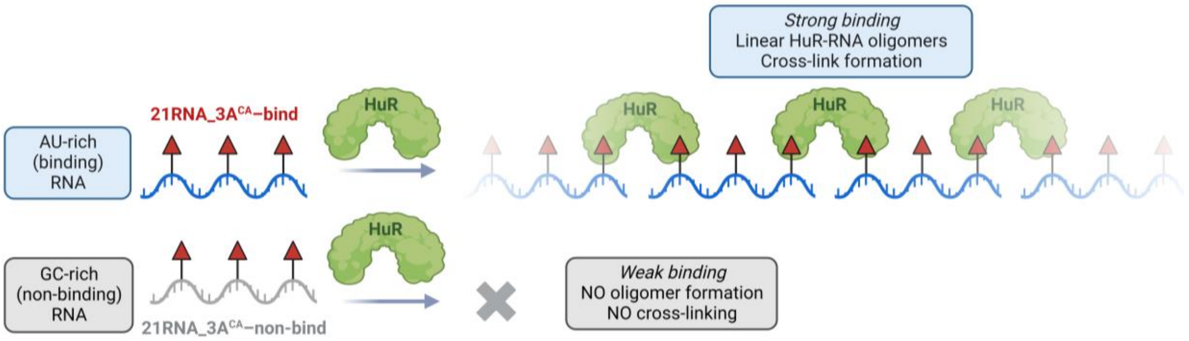

Figure S30. Mechanism of formation of HuR-RNA oligomers.

2.13 Characterization of RNA-protein conjugates by mass analysis (intact ESI-MS or nano-LC-MS/MS)

2.13.1 General procedure for intact ESI-MS analysis of RNA-protein conjugates

The sample was injected onto a MassPREP Micro desalting column (20 µm, 5 X 2.1 mm ID, Waters), desalted and eluted by fast gradient (4 min). Mobile phase A (10 mM ammonium acetate, aq., pH = 9.0) and mobile phase B (acetonitrile) were used for elution. Separation was carried out by AQUITY UPLC I-Class system on-line coupled to Mass Spectrometer Synapt

G2 to acquire mass spectra using electrospray ionization in positive mode. TOF mass range was set from m/z = 500 to 4000. Raw spectrum was subtracted and deconvoluted (MaxEnt1, Waters) to produce the final spectrum.

2.13.2 General procedure I for nano-LC-MS/MS analysis of proteolytic digests

The sample was dissolved in 15 µL of 0.1% trifluoroacetic acid (TFA, aq.) and 5 µL aliquot of the sample was injected on an UltiMate 3000 RSLC nano system coupled to a mass spectrometer Orbitrap Fusion Lumos Tribrid. The peptides were trapped on a PepMap100 column (5 µm, 5 mm by 300 µm internal diameter (ID), Thermo Fisher Scientific) and desalted with 2% acetonitrile in 0.1% formic acid (FA, aq.) at a low rate of 5 µL/min. Eluted peptides were separated using an EASY-Spray PepMap100 C18 analytical column (2 µm, 50 cm by 75 µm ID, Thermo Fisher Scientific). The 30 min elution gradient at a constant flow rate of 300 nL/min was set to start at 5% phase B (0.1% FA in 99.9% acetonitrile) and 95% phase A (0.1% FA, aq.). Then the content of acetonitrile was increased gradually up to 50% of phase B. The Orbitrap mass range was set from m/z = 350 to 2000 in the MS mode and for ions with a charge state 2-6 the fragmentation spectra were acquired. A Proteome Discoverer 2.5 (Thermo Fisher Scientific) was used for peptide and protein identification using Sesquest HS and MS Amanda as search engines and databases of protein sequences and common contaminants.

2.13.3 General procedure II for nano-LC-MS/MS analysis of proteolytic digests

The sample was dissolved in 15 µL of 0.1% trifluoroacetic acid (TFA, aq.) and 5 µL aliquot of the sample was injected on an UltiMate 3000 RSLC nano system coupled to a mass spectrometer Orbitrap Fusion. The peptides were trapped on a PepMap100 column (5 µm, 5 mm by 300 µm internal diameter (ID), Thermo Fisher Scientific) and desalted with 2% acetonitrile in 0.1% formic acid (FA, aq.) at a low rate of 17.5 µL/min. Eluted peptides were separated using an EASY-Spray PepMap100 C18 analytical column (2 µm, 50 cm by 75 µm ID, Thermo Fisher Scientific). The 85 min elution gradient was set to start at 4% phase B (0.1% FA in 99.9% acetonitrile) and 96% phase A (0.1% FA, aq.). Then the content of phase B was gradually increased gradually up to 35% of phase B. The flow rate was set 400 nL/min (0-6 min), 300 nL/min (6.1-64 min) and 400 nL/ (65-85 min). The ion trap mass range was set from m/z = 350 to 1400 in the MS mode and for ions with a charge state 2-7 the fragmentation spectra were acquired. A Proteome Discoverer 2.5 (Thermo Fisher Scientific) was used for peptide and protein identification using Sesquest HS and MS Amanda as search engines and databases of protein sequences and common contaminants.

2.13.4 Intact ESI-MS analysis of 20RNA_1A$^{CA\text{-}HuR}$ conjugate

The reaction mixture (30 µL) containing **20RNA_1A^{CA}** (9 µM, 1.53 equiv., prepared according to above-mentioned procedures: Enzymatic synthesis in semi-preparative scale, Section 2.6.8 and General purification procedure I, Section 2.5.1) in 30 mM HEPES-NaOH buffer (pH = 7.45 at 25 °C) was incubated with HuR protein (5.88 µM, 1 equiv., prepared according to above-mentioned procedure, Section 2.4.2) at 25 °C for 20 h in a thermal cycler (with heated lid at 65 °C). 25 µL of the sample were used for the above-mentioned mass analysis procedure (Section 2.13.1). [ESI-MS analysis: Figure S61 of **20RNA_1A$^{CA\text{-}HuR}$** raw spectrum, Figure S62 of **20RNA_1A$^{CA\text{-}HuR}$** spectrum after deconvolution].

2.13.5 Intact ESI-MS analysis of 20RNA_1A$^{CA\text{-}HIV\text{-}RT}$ conjugate

The reaction mixture (10 µL) containing **20RNA_1A^{CA}** (33.75 µM, 1.63 equiv., prepared according to above-mentioned procedures: Enzymatic synthesis in semi-preparative scale, Section 2.6.8 and General purification procedure I, Section 2.5.1) in 30 mM HEPES-NaOH buffer (pH = 7.45 at 25 °C) was incubated with HIV-RT (20.72 µM, 1 equiv., prepared according to above-mentioned procedure, Section 2.4.1) at 20 °C for 26 h in a thermal cycler (with heated lid at 65 °C). The sample was 5X diluted with 10 mM ammonium acetate buffer (pH = 9.0) and 5 µL aliquot was used for the above-mentioned mass analysis procedure (Section 2.13.1). [ESI-MS analysis: Figure S63 of **20RNA_1A$^{CA\text{-}HIV\text{-}RT}$** raw spectrum, Figure S64 of **20RNA_1A$^{CA\text{-}HIV\text{-}RT}$** spectrum after deconvolution].

2.13.6 LC-MS analysis of conjugation mixture of 20RNA_1A^{CA} with HIV-RT protein

The reaction mixture (50 µL) containing **20RNA_1A^{CA}** (15 µM, 1.5 equiv., prepared according to above-mentioned procedures: Enzymatic synthesis in preparative scale, Section 2.6.12 and General purification procedure III, Section 2.5.3) in 25 mM HEPES-NaOH buffer (pH = 7.45 at 25 °C) was incubated with HIV-RT (10 µM, 1 equiv., prepared according to above-mentioned procedure, Section 2.4.1) at 20 °C for 22 h in a thermal cycler (with heated lid at 65 °C). After this time, the sample was concentrated to 20 µL using Amicon Ultra-0.5 centrifugal concentrator (10 kDa MWCO). LC-MS analysis was carried out on Agilent 1290 Infinity II Bio system. 20 µL of the purified reaction mixture were injected on Waters ACQUITY Premier CSH C18 1.7 µm, 2.1 X 150 mm column. [LC-MS separation chromatogram: Figure S31]. [ESI-MS spectra: Figures S65-S70]. Separation was performed at 45 °C column temperature with flow rate 0.25 mL/min with mobile phases: A (36 mM TEAB + 0.25 % Et_3N in water) and B (36 mM TEAB + 0.25 % Et_3N in 90% MeCN). The gradient was as follows:

**Time (min)**	**Mobile phase A (%)**	**Mobile phase B (%)**
0.00	95	5
5.00	95	5
8.00	65	35
28.00	35	65
28.01	0	100
32.00	0	100

MS settings were as follows: capillary voltage –5 kV, drying gas flow 12 L/min, nebulizer pressure 35 psig, drying gas temperature 350 °C, fragmentor 70 V, mass range 500 – 3000 m/z, 0.1 Da step size, 1.46 sec per scan cycle.

Note: LC-MS system required extensive cleaning from triethylamine contamination with 50 % iPrOH, 40% H_2O, 10% AcOH mixture to enable further use for IP-RP-HPLC oligonucleotide measurements.

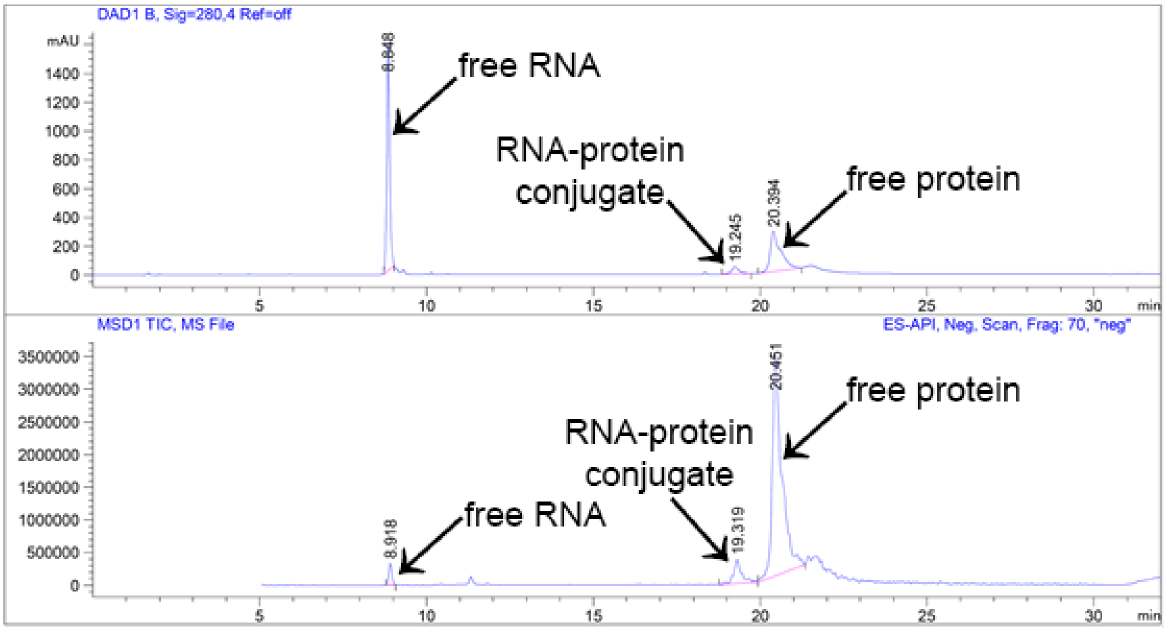

Figure S31. LC-MS separation chromatogram of conjugation mixture of **20RNA_1A^{CA}** with HIV-RT protein.

Quantitative data from UV (280 nm) chromatogram:

Peak	Retention time (min)	Width (min)	Area (mAU·s)	Height (mAU)	Area (%)
2 (conjugate)	19.245	0.2431	741.80029	50.85138	10.7
3 (free protein)	20.394	0.3680	6207.61719	281.12140	89.3

The peak 1 at 8.848 min (UV)/ 8.918 min (TIC) is free unreacted, modified RNA.

2.13.7 Preparation of 20RNA_1A$^{CA\text{-}HuR}$ conjugate digest for nano-LC-MS/MS analysis

The reaction mixture (100 µL) containing **20RNA_1A^{CA}** (7.5 µM, 1.23 equiv., prepared according to above-mentioned procedures: Enzymatic synthesis in semi-preparative scale, Section 2.6.8 and General purification procedure I, Section 2.5.1) in 30 mM HEPES-NaOH buffer (pH = 7.45 at 25 °C) was incubated with HuR protein (6.08 µM, 1 equiv., prepared according to above-mentioned procedure, Section 2.4.2) at 37 °C for 25 h in a thermal cycler (with heated lid at 65 °C). After this time, urea (8 M) and DTT (10 mM) were added to the mixture and incubated at 37 °C for 1 h with shaking (800 rpm) in a thermal block. Then 9.67 µL of freshly prepared saturated solution of iodoacetamide (dissolved in H_2O) were added to the mixture and further incubated at 37 °C for 1 h with shaking (800 rpm) in a thermal block. The solution was diluted with 700 µL of H_2O and 175 µL of the cleavage buffer (500 mM Tris, 10 mM $CaCl_2$, pH = 8.0 at 25 °C). Then 0.96 µg of chymotrypsin protease (1:25 protease/protein ratio) were added to the mixture and incubated at 37 °C with gentle rotation on Hula-mixer overnight. The protease was denatured by heating at 70 °C for 10 min and the sample was concentrated on vacuum concentrator. Then the solution was purified (General purification procedure I, Section 2.5.1) and eluted in 50 µL of H_2O followed by evaporation to dryness. The solid sample was dissolved in 200 µL of concentrated

hydrofluoric acid (HF)[9] and incubated overnight at 4 °C. The HF was evaporated and the sample was purified on C18 spin columns according to standard supplier´s protocol. Elution was performed with 3X 50 μL of 70% acetonitrile and the sample was evaporated to dryness prior to mass analysis (Section 2.13.2). [nano-LC-MS/MS spectrum: Figure S71].

2.13.8 Preparation of 20RNA_1A$^{CA\text{-}HIV\text{-}RT}$ conjugate digest for nano-LC-MS/MS analysis

The reaction mixture (25 μL) containing **20RNA_1A^{CA}** (30 μM, 1.13 equiv., prepared according to above-mentioned procedures: Enzymatic synthesis in semi-preparative scale, Section 2.6.8 and General purification procedure I, Section 2.5.1) in 30 mM HEPES-NaOH buffer (pH = 7.45 at 25 °C) was incubated with HIV-RT (26.64 μM, 1 equiv., prepared according to above-mentioned procedure, Section 2.4.1) at 20 °C for 25 h in a thermal cycler (with heated lid at 65 °C). After this time, 75 μL of H_2O were added to the mixture followed by urea (8 M) and DTT (10 mM). The mixture was incubated at 37 °C for 1 h with shaking (800 rpm) in a thermal block. Then 9.67 μL of freshly prepared saturated solution of iodoacetamide (dissolved in H_2O) were added to the mixture and further incubated at 37 °C for 1 h with shaking (800 rpm) in a thermal block. The solution was diluted with 700 μL of H_2O and 175 μL of the cleavage buffer (500 mM ammonium bicarbonate, pH = 8.0 at 25 °C). Then 3.12 μg of trypsin protease (1:25 protease/protein ratio) were added to the mixture and incubated at 37 °C with gentle rotation on Hula-mixer overnight. The protease was denatured by heating at 70 °C for 10 min and the sample was concentrated on vacuum concentrator. Then the solution was purified (General purification procedure I, Section 2.5.1) and eluted in 50 μL of H_2O followed by evaporation to dryness. The solid sample was dissolved in 200 μL of concentrated hydrofluoric acid (HF)[9] and incubated overnight at 4 °C. The HF was evaporated and the sample was purified on C18 spin columns according to standard supplier´s protocol. Elution was performed with 3X 50 μL of 70% acetonitrile and the sample was evaporated to dryness prior to mass analysis (Section 2.13.2). [nano-LC-MS/MS spectra: Figures S72, S73].

2.13.9 Preparation of 20RNA_1A$^{CA\text{-}hAgo2}$ conjugate digest for nano-LC-MS/MS analysis

The reaction mixture (105 μL) containing **20RNA_1A^{CA}** (7.86 μM, 1.23 equiv., prepared according to above-mentioned procedures: Enzymatic synthesis in semi-preparative scale, Section 2.6.8 and General purification procedure I, Section 2.5.1) in 30 mM HEPES-NaOH buffer (pH = 7.45 at 25 °C) was incubated with hAgo2 protein (6.38 μM, 1 equiv., Sino Biological) at 37 °C for 24 h in a thermal cycler (with heated lid at 65 °C). After this time, urea (8 M) and DTT (10 mM) were added to the mixture and incubated at 37 °C for 1 h with shaking (800 rpm) in a thermal block. Then 10.15 μL of freshly prepared saturated solution of iodoacetamide (dissolved in H_2O) were added to the mixture and further incubated at 37 °C for 1 h with shaking (800 rpm) in a thermal block. The solution was diluted with 729 μL of H_2O and 216 μL of the cleavage buffer (500 mM ammonium bicarbonate, pH = 8.0 at 25 °C). Then 2.68 μg of trypsin protease (1:25 protease/protein ratio) were added to the mixture and incubated at 37 °C with gentle rotation on Hula-mixer overnight. The protease was denatured by heating at 70 °C for 10 min and the sample was concentrated on vacuum concentrator. Then the solution was purified (General purification procedure I, Section 2.5.1)

and eluted in 50 μL of H_2O followed by evaporation to dryness. The solid sample was dissolved in 200 μL of concentrated hydrofluoric acid (HF)[9] and incubated overnight at 4 °C. The HF was evaporated and the sample was purified on C18 spin columns according to standard supplier´s protocol. Elution was performed with 3X 50 μL of 70% acetonitrile and the sample was evaporated to dryness prior to mass analysis (Section 2.13.2). [nano-LC-MS/MS spectra: Figures S74-S77]. The sample was prepared in two more replicates. One following exactly the same procedure with 24 h incubation length for RNA-protein cross-linking [nano-LC-MS/MS spectra: Figures S78-S80] and the other one following the same procedure just with shorter 6 h incubation time for RNA-protein cross-linking reaction [nano-LC-MS/MS spectra: Figures S81-S83]. The samples were purified (C18 spin columns), eluted with 3X 50 μL of 70% acetonitrile and evaporated to dryness prior to mass analysis (Section 2.13.3).

2.13.9.1 Supplementary note no. 2 – Discussion of the specificity of the cross-linking with different cysteins of hAgo2

Two replicates of cross-linking experiment of modified **20RNA_1A^{CA}** and hAgo2 protein were carried out to enhance identification confidence and third replicate was performed with reduced (6 h) incubation time to showcase cross-linking efficiency in shorter period. From all three experiments we identified together five conjugated cysteines. We mapped cross-linked amino acids on the available crystal structure of Ago2-siRNA complex (PDB: 4W5N) (Figure S84 in SI). Three cysteines have close interaction with RNA according to this model: Cys362 (8.6 Å), Cys84 (7.0 Å) and Cys345 (6.7 Å). Other two cysteines are more distant. Cys206 (18.3 Å) is part of disordered linker L1 region and Cys480 (15.8 Å) is on the edge of the RNA binding groove. These data illustrate approximate distance required for cross-linking, however, we assume that certain spatial orientation and accessibility of the particular amino acid might be also required for efficient conjugation, since many cysteines in proximity to RNA were not captured. We expect, that adjusting length and flexibility of the linker connecting reactive group and nucleobase would allow further tuning of crosslinking specificity and efficiency. Above-mentioned measurements are based on crystal structure, which is static snapshot of rather dynamic interaction (as discussed in R. Kong, L. Xu, L. Piao, D. Zhang, T. J. Hou, S. Chang, *Chem. Biol. Drug Des.* **2017**, *90*, 753–763). Further insight would be gained through other biophysical methods (Bio-NMR, EPR, etc.).

There are also general proteomic workflow weaknesses, which might lead to loss of cross-linked, yet unidentified amino acids:

1) For hAgo2 we used trypsin protease, which produced best coverage in screening with non-cross-linked protein. However, this might not be the same case for cross-linked conjugate, since RNA can hinder trypsin cleavage sites and resulting long peptides are not identified by nano-LC-MS/MS. Several proteases could be used for digestion of the conjugates in question to enhance protein coverage.

2) For enrichment of peptide-RNA conjugates we used silica-based spin columns. Since retention here is based on RNA portion of the molecule, larger peptides can diminish capture of the conjugate, thereby leading to its loss. Complementary enrichment methods can be considered to minimize this bottleneck (e.g., tagging RNA with biotin for streptavidin enrichment or poly(T) oligo capture of mRNA).

3) For preliminary desalting and removal of HF traces we used C18 columns. Retention here (and on LC-MS separation column downstream) are based on hydrophobicity. Different purification methods for polar peptides (e.g. graphite columns) might be used to broaden overall coverage.

3 Copies of mass spectra

3.1 Copies of MS-MALDI-TOF spectra

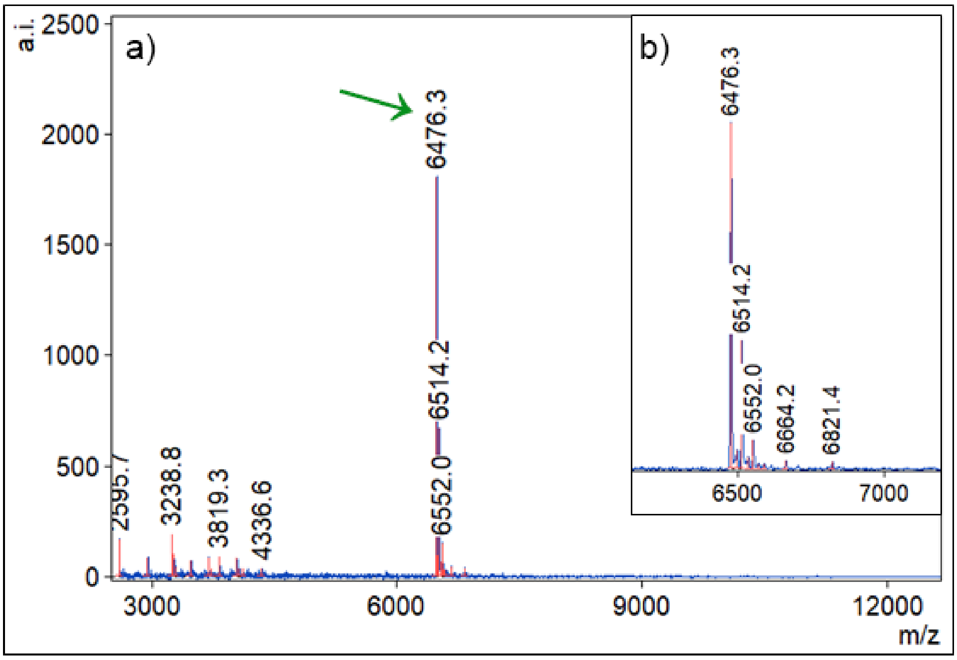

Figure S32. MS-MALDI-TOF spectrum of **20RNA_1A**; a) full spectrum; b) magnified area of interest; calculated: 6472.24 Da; found: 6476.3 Da; Δ = 4.06 Da. The peak at *m*/*z* = 6514.2 Da can be assigned to the adduct [**20RNA_1A** + K^+].

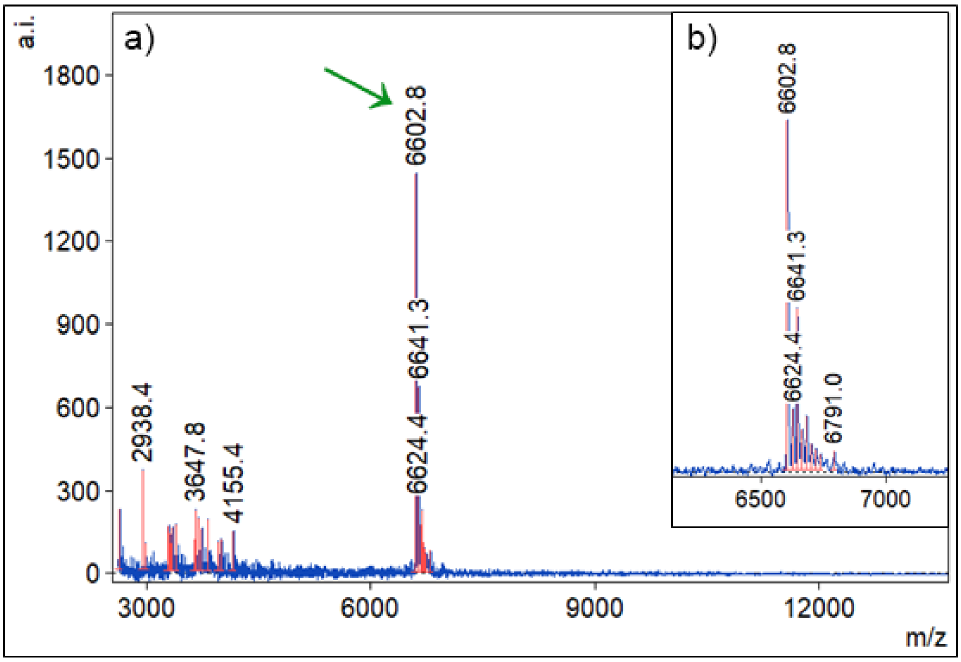

Figure S33. MS-MALDI-TOF spectrum of **20RNA_1A^{CA}**; a) full spectrum; b) magnified area of interest; calculated: 6601.2 Da; found: 6602.8 Da; Δ = 1.6 Da. The peak at *m*/*z* = 6641.3 Da can be assigned to the adduct [**20RNA_ 1A^{CA}** + K^+]. The peak at *m*/*z* = 6624.4 Da can be assigned to the adduct [**20RNA_ 1A^{CA}** + Na^+].

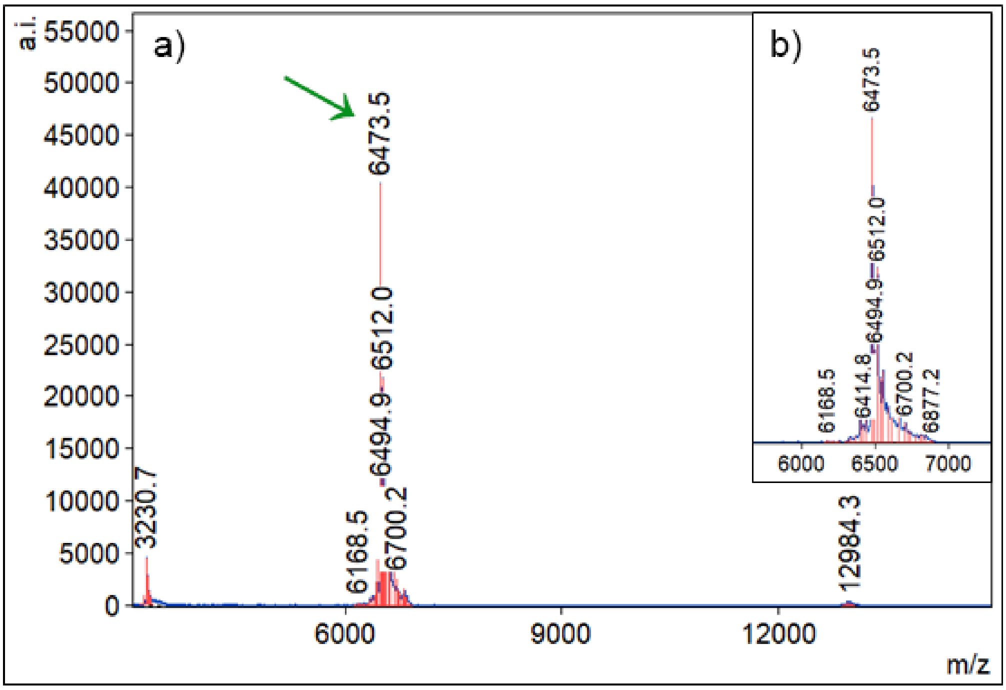

Figure S34. MS-MALDI-TOF spectrum of **20RNA_1A**; a) full spectrum; b) magnified area of interest; calculated: 6472.14 Da; found: 6473.5 Da; Δ = 1.36 Da. The peak at *m/z* = 6512.0 Da can be assigned to the adduct [**20RNA_ 1A** + K^+]. The peak at *m/z* = 6494.9 Da can be assigned to the adduct [**20RNA_ 1A** + Na^+].

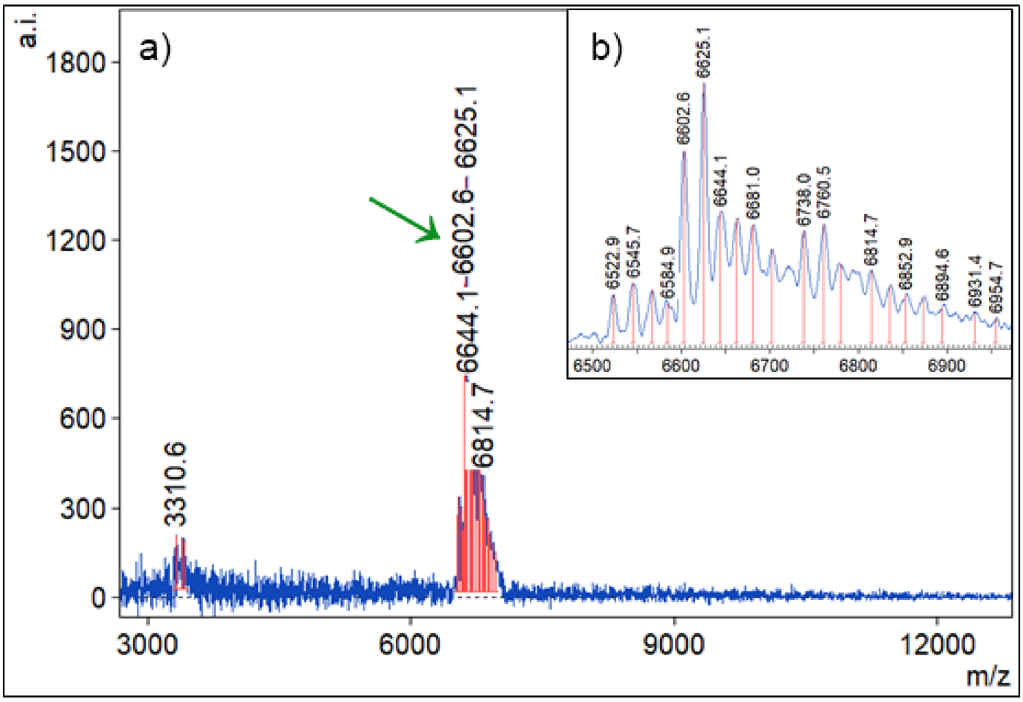

Figure S35. MS-MALDI-TOF spectrum of **20RNA_1A^{CA}**; a) full spectrum; b) magnified area of interest; calculated: 6601.2 Da; found: 6602.6 Da; Δ = 1.4 Da. The peak at *m/z* = 6625.1 Da can be assigned to the adduct [**20RNA_ 1A^{CA}** + Na^+]. The peak at *m/z* = 6644.1 Da can be assigned to the adduct [**20RNA_ 1A^{CA}** + K^+].

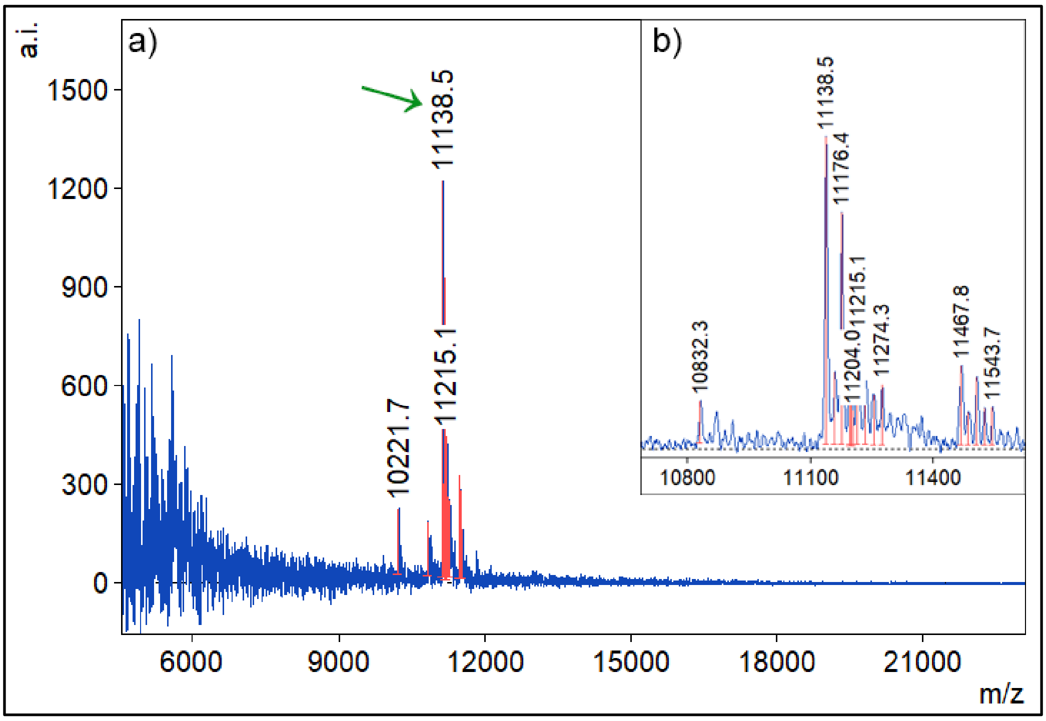

Figure S36. MS-MALDI-TOF spectrum of **35RNA_1A**; a) full spectrum; b) magnified area of interest; calculated: 11138.31 Da; found: 11138.50 Da; Δ = 0.2 Da. The peak at m/z = 11176.4 Da can be assigned to the adduct [**35RNA_ 1A** + K^+].

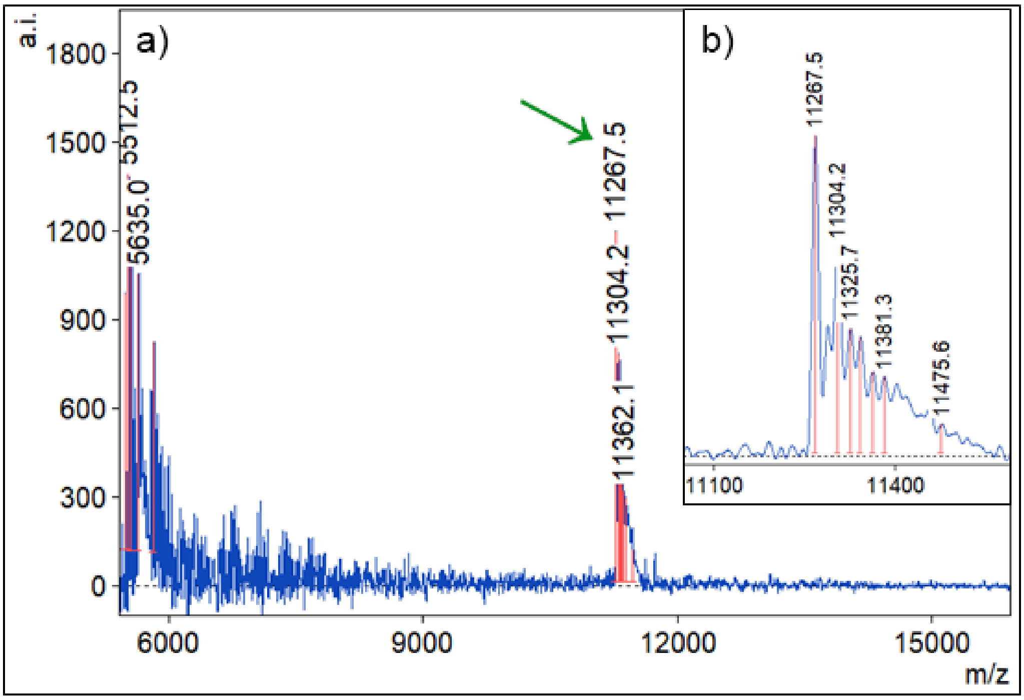

Figure S37. MS-MALDI-TOF spectrum of **35RNA_1A^{CA}**; a) full spectrum; b) magnified area of interest; calculated: 11266.86 Da; found: 11267.5 Da; Δ = 0.64 Da. The peak at m/z = 11304.2 Da can be assigned to the adduct [**35RNA_1A^{CA}** + K^+]. The peak at m/z = 11325.7 Da can be assigned to the adduct [**35RNA_1A^{CA}** + K^+ + Na^+].

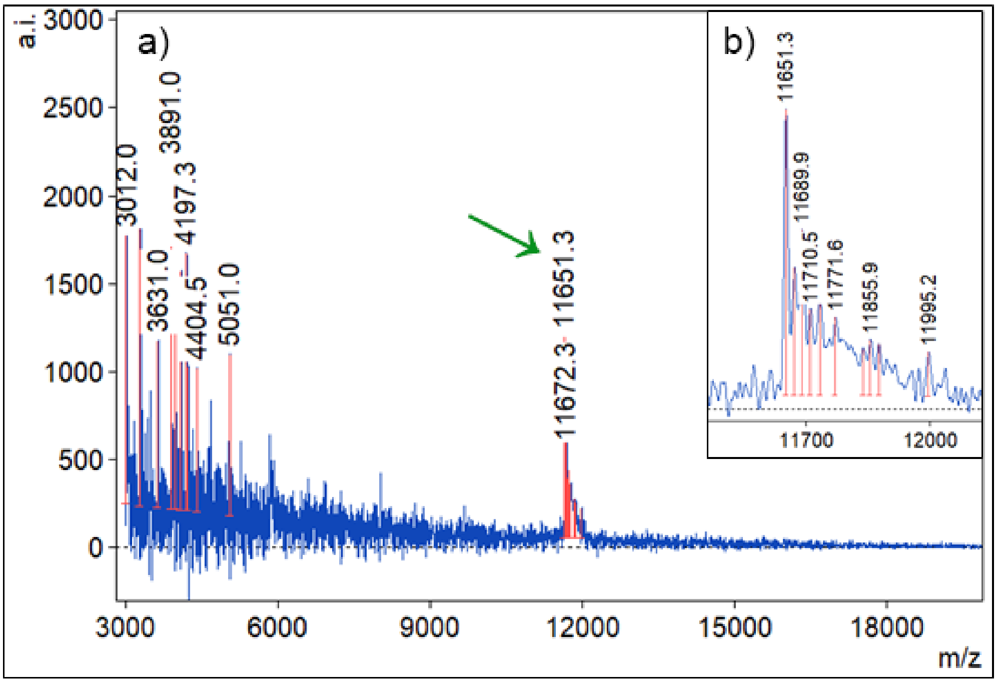

Figure S38. MS-MALDI-TOF spectrum of **35RNA_3A^{CA}**; a) full spectrum; b) magnified area of interest; calculated: 11650.1 Da; found: 11651.3 Da; Δ = 1.2 Da. The peak at *m/z* = 11689.9 Da can be assigned to the adduct [**35RNA_ 3A^{CA}** + K^+].

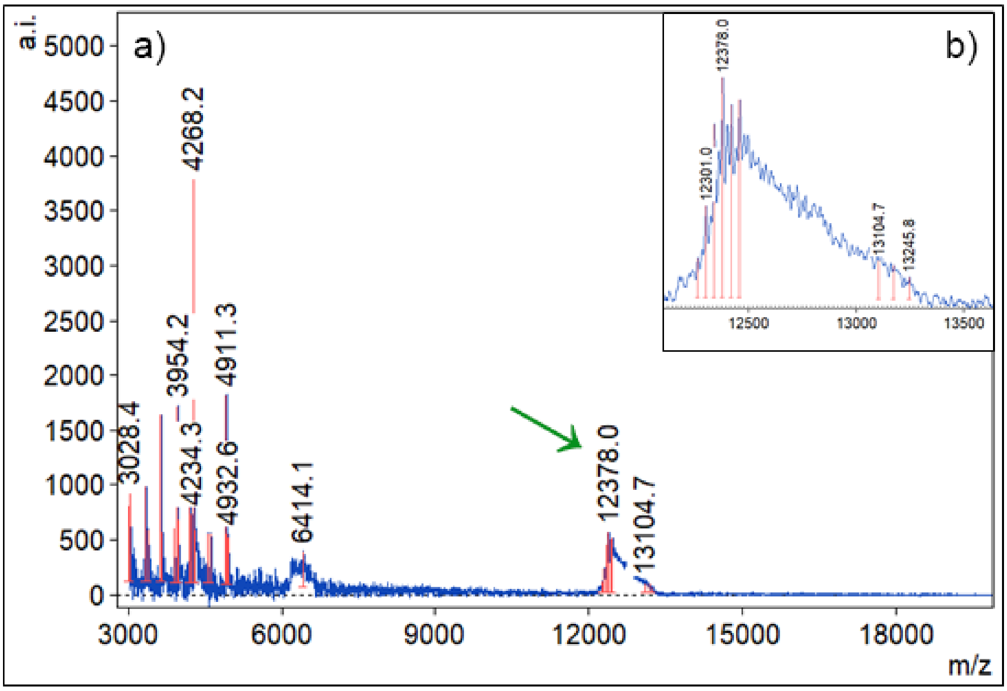

Figure S39. MS-MALDI-TOF spectrum of **35RNA_7A^{CA}**; a) full spectrum; b) magnified area of interest; calculated: 12376.57 Da; found: 12378.0 Da; Δ = 1.43 Da.

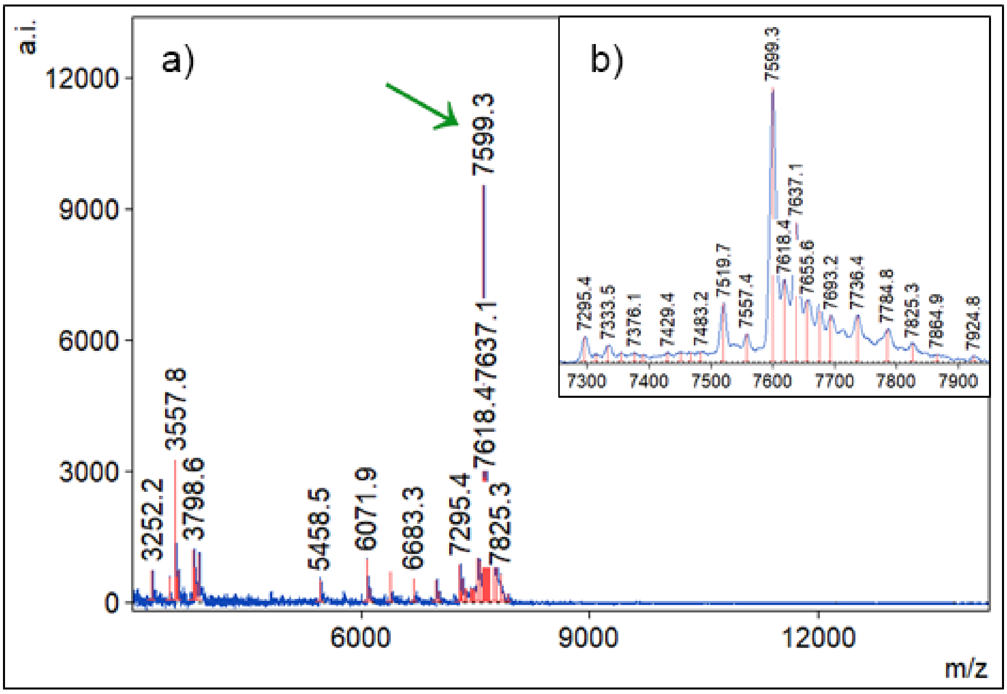

Figure S40. MS-MALDI-TOF spectrum of **21RNA_1A-Cy5**; a) full spectrum; b) magnified area of interest; calculated: 7595.77 Da; found: 7599.3 Da; Δ = 3.53 Da. The peak at *m/z* = 7637.1 Da can be assigned to the adduct [**21RNA_1A-Cy5** + K^+].

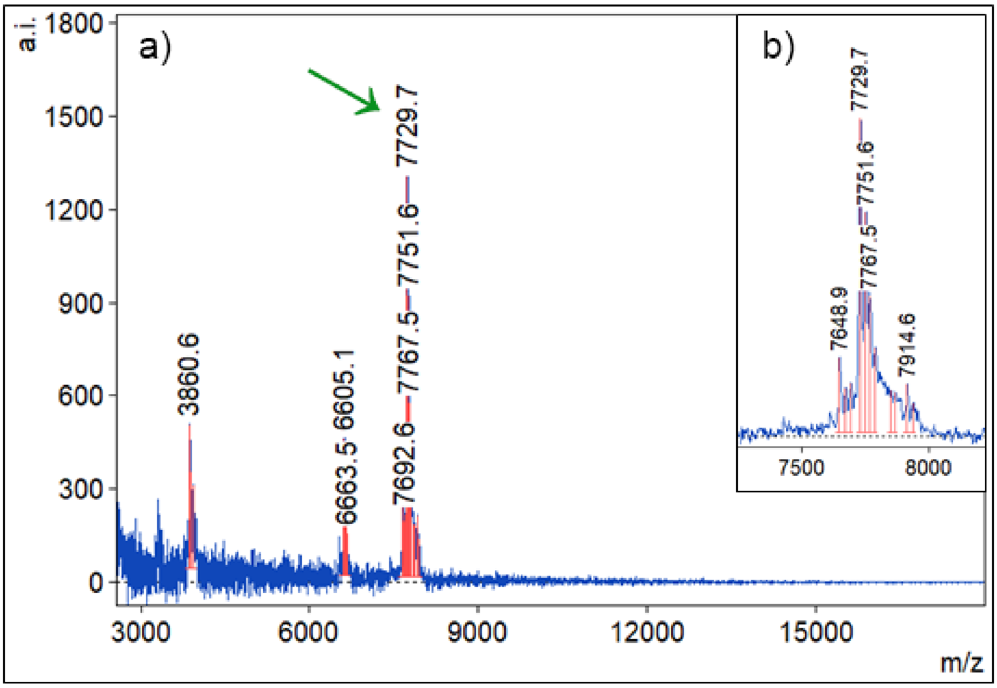

Figure S41. MS-MALDI-TOF spectrum of **21RNA_1A^{CA}-Cy5**; a) full spectrum; b) magnified area of interest; calculated: 7724.33 Da; found: 7729.7 Da; Δ = 5.37 Da. The peak at *m/z* = 7751.6 Da can be assigned to the adduct [**21RNA_1A^{CA} -Cy5** + Na^+]. The peak at *m/z* = 7767.5 Da can be assigned to the adduct [**21RNA_1A^{CA} -Cy5** + K^+].

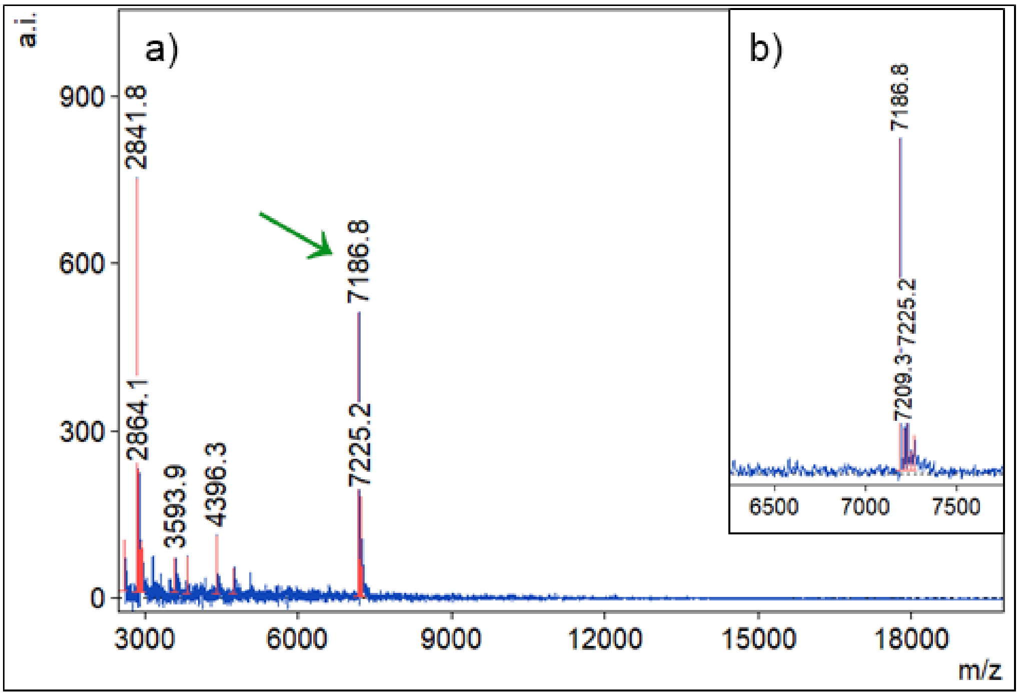

Figure S42. MS-MALDI-TOF spectrum of **21RNA_1A-Bio**; a) full spectrum; b) magnified area of interest; calculated: 7183.27 Da; found: 7186.8 Da; Δ = 3.53 Da. The peak at *m/z* = 7225.2 Da can be assigned to the adduct [**21RNA_1A-Bio** + K^+].

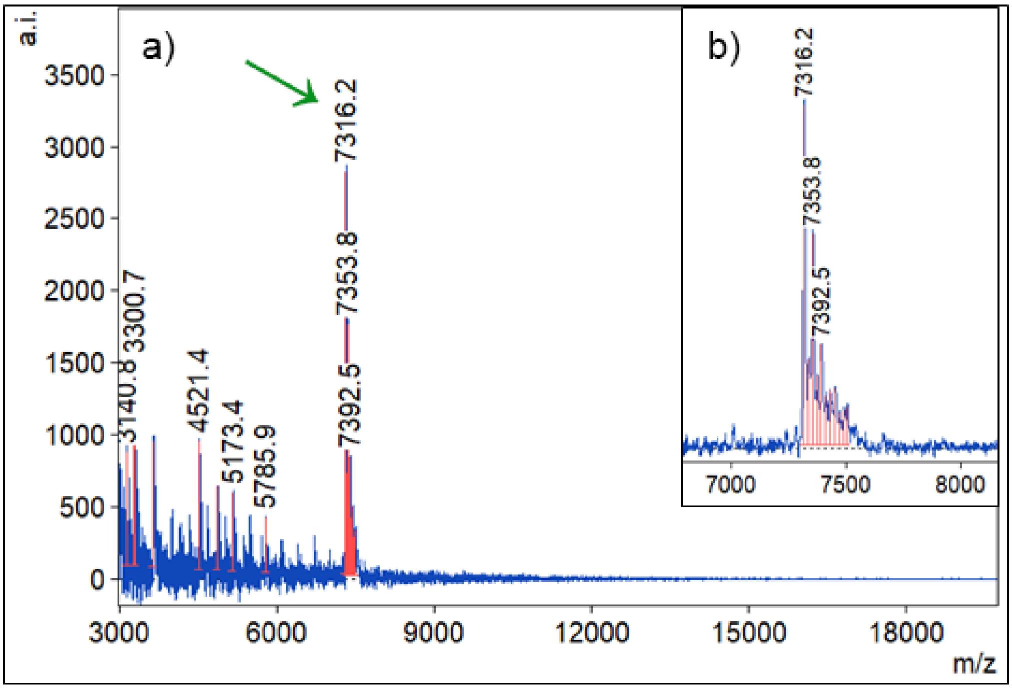

Figure S43. MS-MALDI-TOF spectrum of **21RNA_1A^{CA}-Bio**; a) full spectrum; b) magnified area of interest; calculated: 7312.23 Da; found: 7316.2 Da; Δ = 3.97 Da. The peak at *m/z* = 7353.8 Da can be assigned to the adduct [**21RNA_1A^{CA} -Bio** + K^+]. The peak at *m/z* = 7392.5 Da can be assigned to the adduct [**21RNA_1A^{CA} -Bio** + $2K^+$].

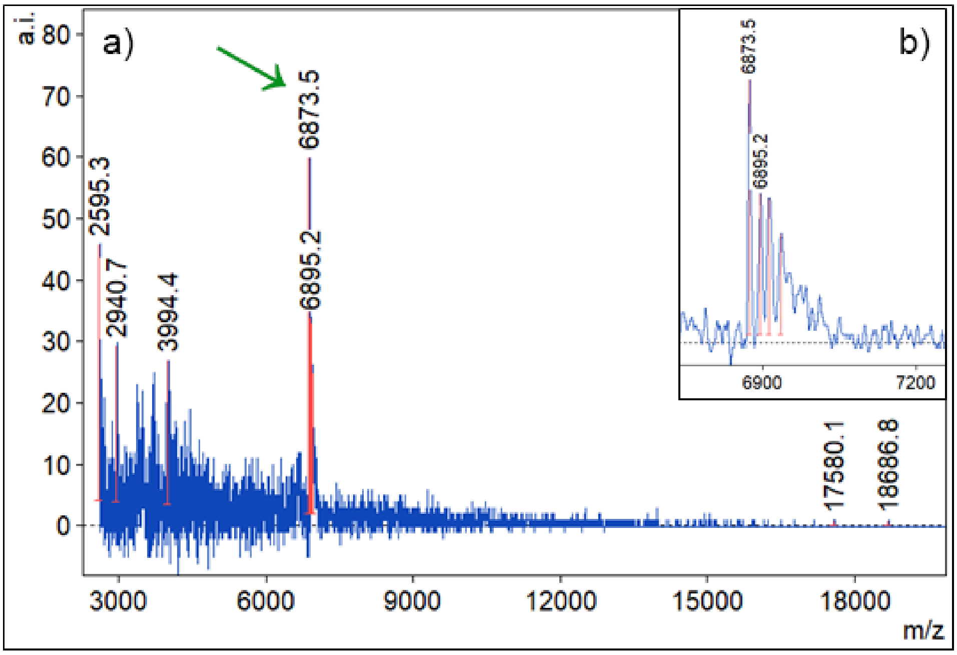

Figure S44. MS-MALDI-TOF spectrum of **20RNA_1A$^{\mathbf{CA\text{-}GSH}}$** conjugate; a) full spectrum; b) magnified area of interest; calculated: 6872.07 Da; found: 6873.5 Da; Δ = 1.43 Da. The peak at *m*/*z* = 6895.2 Da can be assigned to the adduct [**20RNA_1A$^{\mathbf{CA\text{-}GSH}}$** + Na^+].

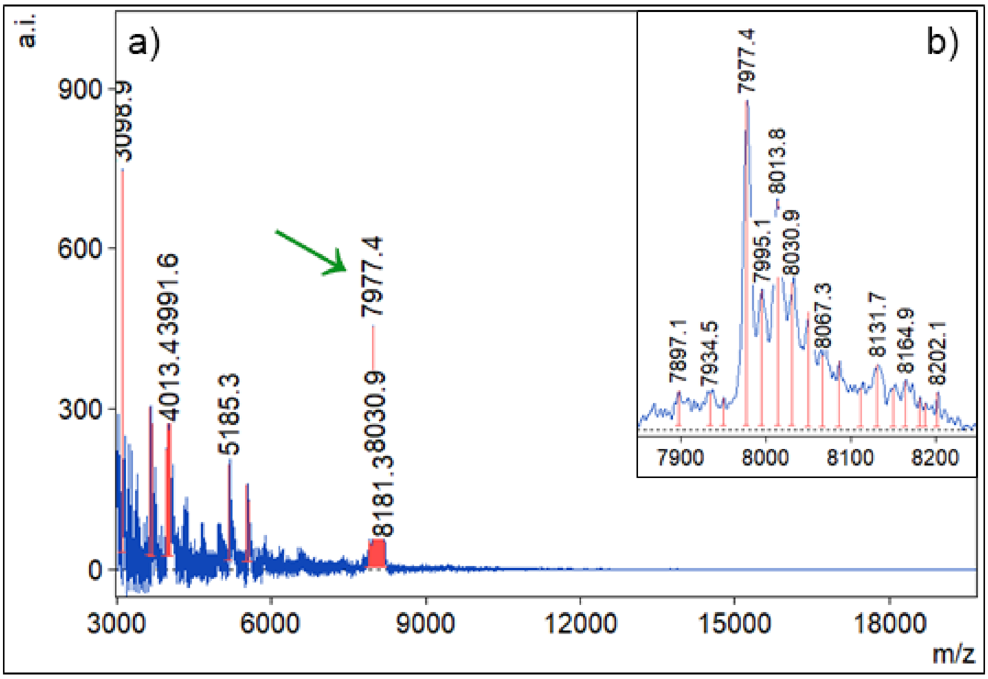

Figure S45. MS-MALDI-TOF spectrum of **20RNA_1A$^{\mathbf{CA\text{-}pept\text{-}(+)\text{-}C}}$** conjugate; a) full spectrum; b) magnified area of interest; calculated: 7974.55 Da; found: 7977.4 Da; Δ = 2.85 Da. The peak at *m*/*z* = 8013.8 Da can be assigned to the adduct [**20RNA_1A$^{\mathbf{CA\text{-}pept\text{-}(+)\text{-}C}}$** + K^+].

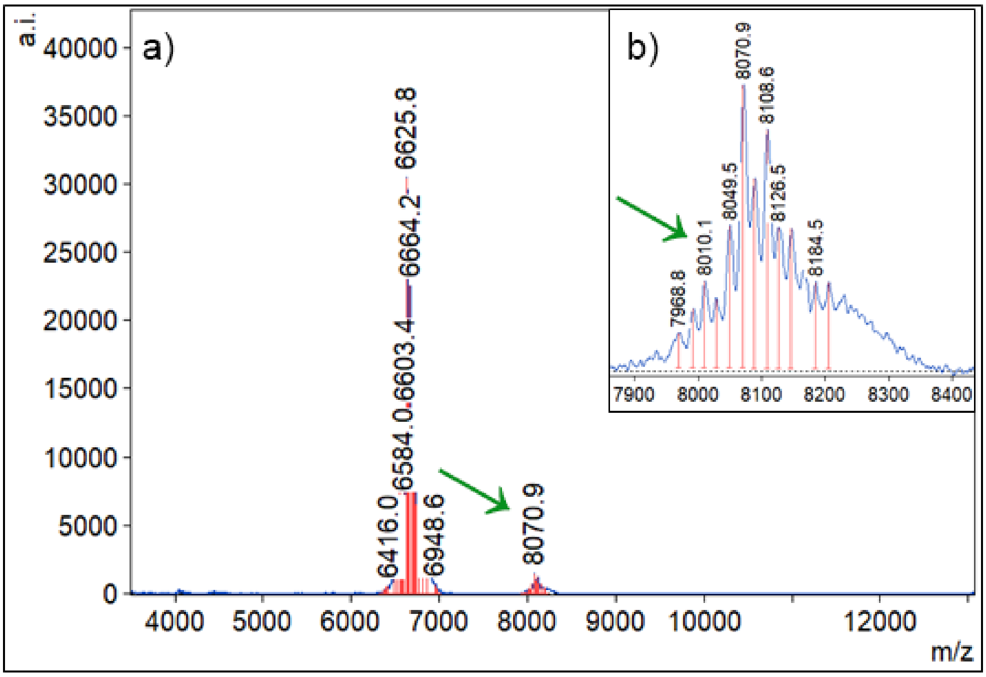

Figure S46. MS-MALDI-TOF spectrum of **20RNA_1A$^{\mathbf{CA\text{-}pept\text{-}(+)\text{-}H}}$** conjugate; a) full spectrum; b) magnified area of interest; calculated: 8008.55 Da; found: 8010.1 Da; Δ = 1.55 Da. The peak at *m/z* = 6603.2 Da can be assigned to unreacted **20RNA_1A$^{\mathbf{CA}}$**. The peak at *m/z* = 6625.8 Da can be assigned to the adduct [**20RNA_1A$^{\mathbf{CA}}$** + Na^+]. The peak at *m/z* = 6664.2 Da can be assigned to the adduct [**20RNA_1A$^{\mathbf{CA}}$** + Na^+ + K^+]. The peak at *m/z* = 8049.5 Da can be assigned to the adduct [**20RNA_1A$^{\mathbf{CA\text{-}pept\text{-}(+)\text{-}H}}$** + K^+]. The peak at *m/z* = 8070.9 Da can be assigned to the adduct [**20RNA_1A$^{\mathbf{CA\text{-}pept\text{-}(+)\text{-}H}}$** + K^+ + Na^+]. The peak at *m/z* = 8108.6 Da can be assigned to the adduct [**20RNA_1A$^{\mathbf{CA\text{-}pept\text{-}(+)\text{-}H}}$** + $2K^+$ + Na^+].

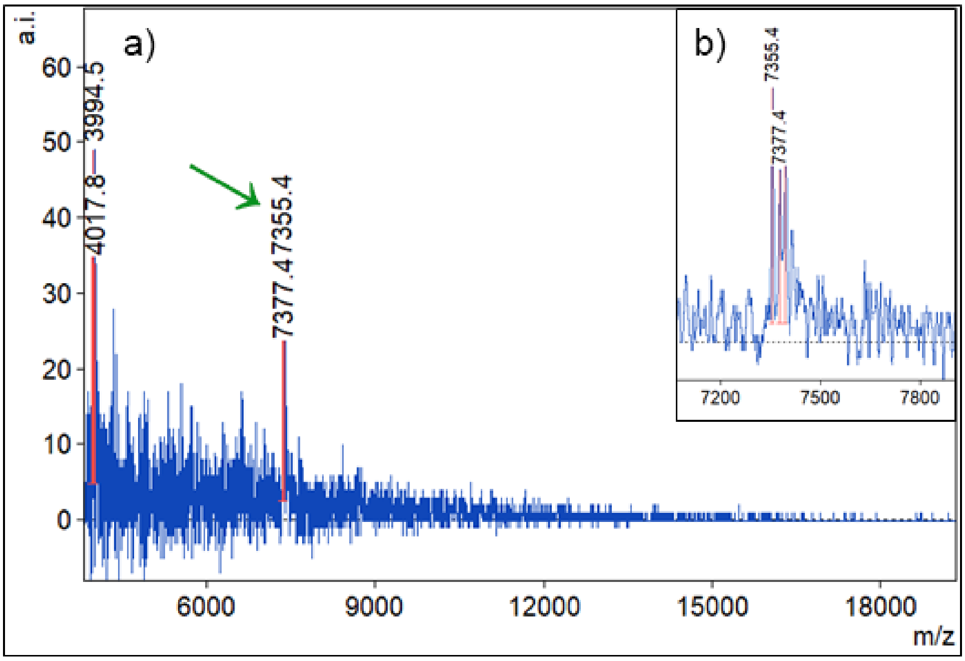

Figure S47. MS-MALDI-TOF spectrum of **20RNA_1A$^{\mathbf{CA\text{-}biotin}}$** conjugate; a) full spectrum; b) magnified area of interest; calculated: 7352.75 Da; found: 7355.4 Da; Δ = 2.65 Da. The peak at *m/z* = 7377.4 Da can be assigned to the adduct [**20RNA_1A$^{\mathbf{CA\text{-}biotin}}$** + Na^+].

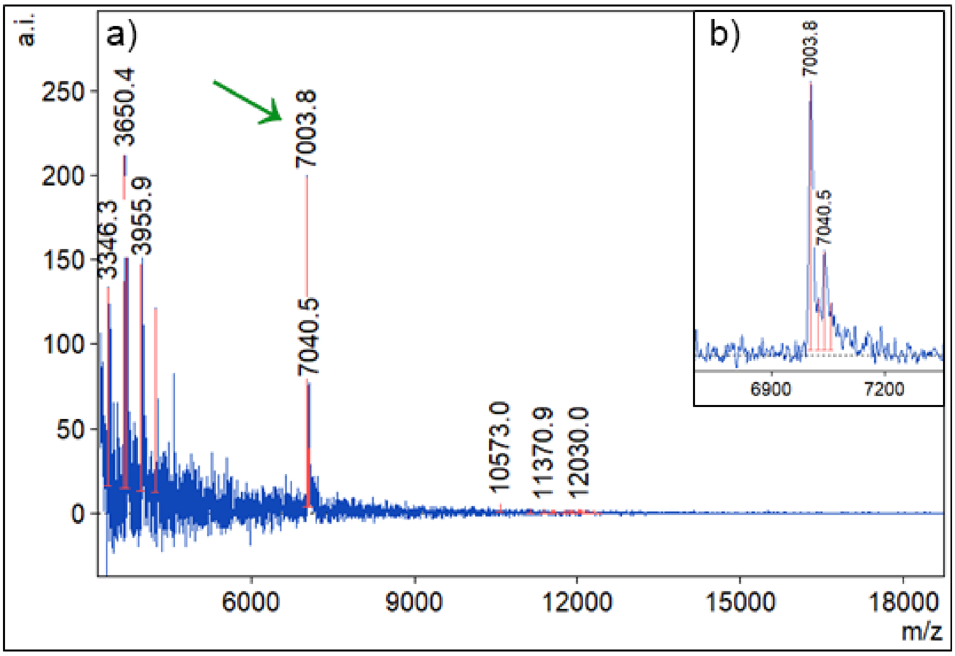

Figure S48. MS-MALDI-TOF spectrum of **20RNA_1A$^{\text{CA-FL}}$** conjugate; a) full spectrum; b) magnified area of interest; calculated: 7002.22 Da; found: 7003.8 Da; $\Delta = 1.58$ Da. The peak at *m*/*z* = 7040.5 Da can be assigned to the adduct [**20RNA_1A$^{\text{CA-FL}}$** + K^+].

3.2 Copies of ESI-MS spectra

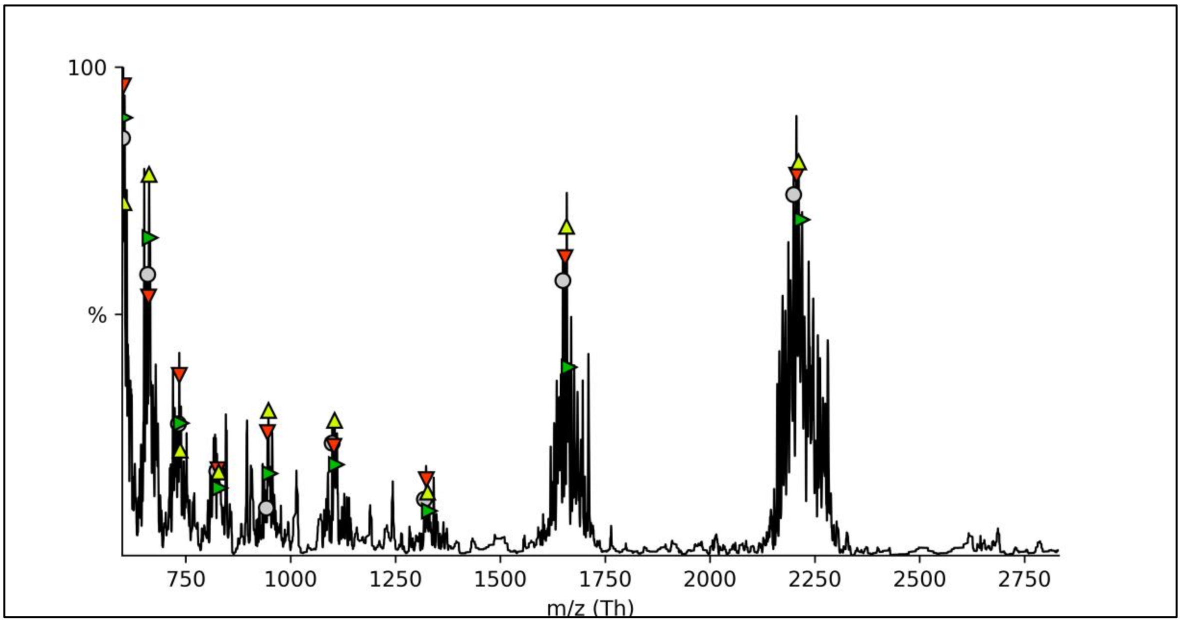

Figure S49. MS spectrum from LC-MS analysis of bioconjugation of **20RNA_1A^{CA}** and pept-(+)-C. MS spectrum of peak 2 (free, unreacted RNA). Raw mass spectrum.

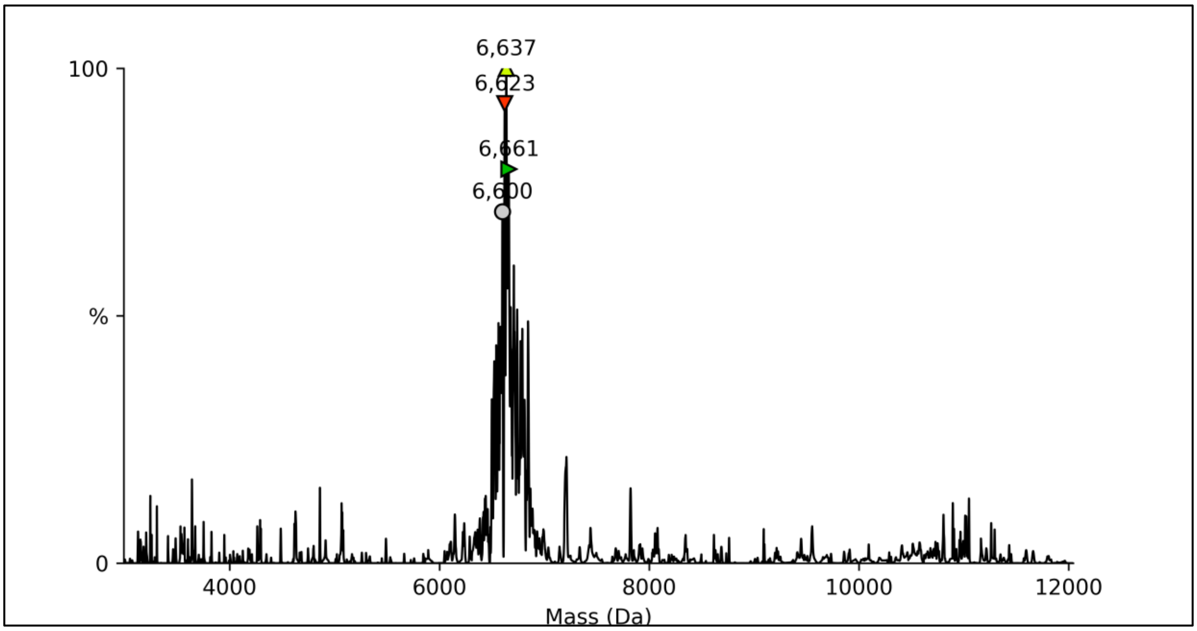

Figure S50. MS spectrum from LC-MS analysis of bioconjugation of **20RNA_1A$^{\mathbf{CA}}$** and pept-(+)-C. MS spectrum of peak 2 (free, unreacted RNA). Deconvoluted mass spectrum. calculated: 6601.2 Da; found: 6600.0 Da; Δ = 1.2 Da. The peak at *m*/*z* = 6623.0 Da can be assigned to the adduct [**20RNA_ 1A$^{\mathbf{CA}}$** + Na^+]. The peak at *m*/*z* = 6637.0 Da can be assigned to the adduct [**20RNA_ 1A$^{\mathbf{CA}}$** + K^+]. The peak at *m*/*z* = 6661.0 Da can be assigned to the adduct [**20RNA_ 1A$^{\mathbf{CA}}$** + Na^+ + K^+].

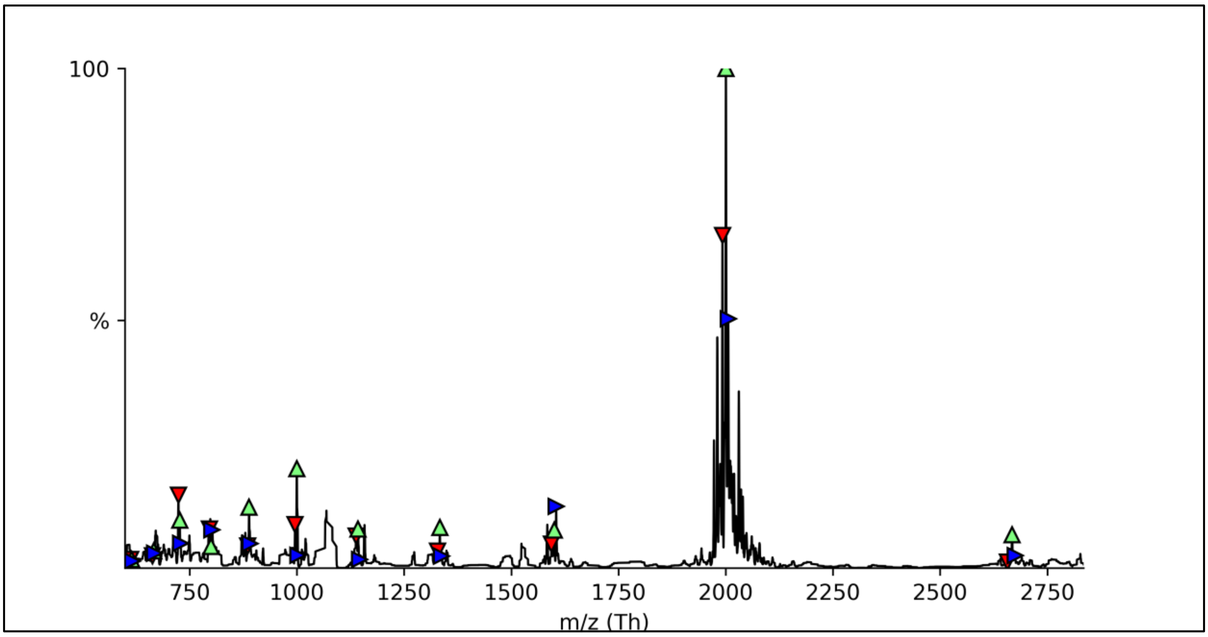

Figure S51. MS spectrum from LC-MS analysis of bioconjugation of **20RNA_1A$^{\mathbf{CA}}$** and pept-(+)-C. MS spectrum of peak 1 (conjugate **20RNA_1A$^{\mathbf{CA\text{-}pept\text{-}(+)\text{-}C}}$**). Raw mass spectrum.

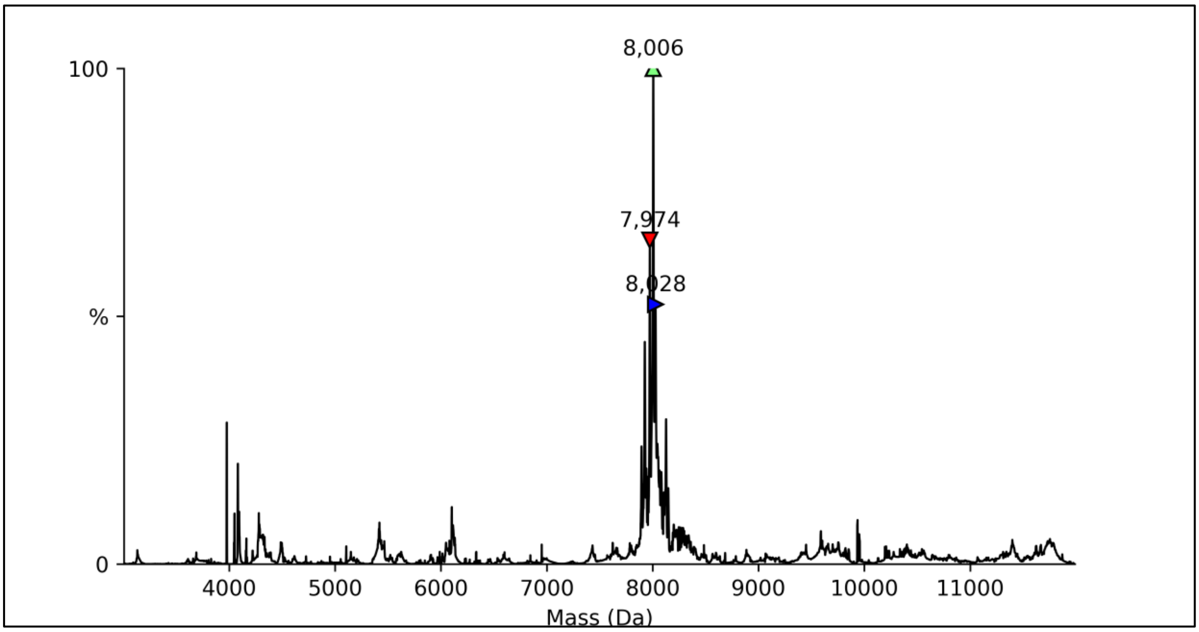

Figure S52. MS spectrum from LC-MS analysis of bioconjugation of **20RNA_1A^{CA}** and pept-(+)-C. MS spectrum of peak 1 (conjugate **20RNA_1A$^{CA-pept-(+)-C}$**). Deconvoluted mass spectrum. calculated: 7974.55 Da; found: 7974.0 Da; Δ = 0.55 Da. The peak at *m*/*z* = 8006.0 Da can be assigned to the adduct [**20RNA_1A$^{CA-pept-(+)-C}$** + MeOH].

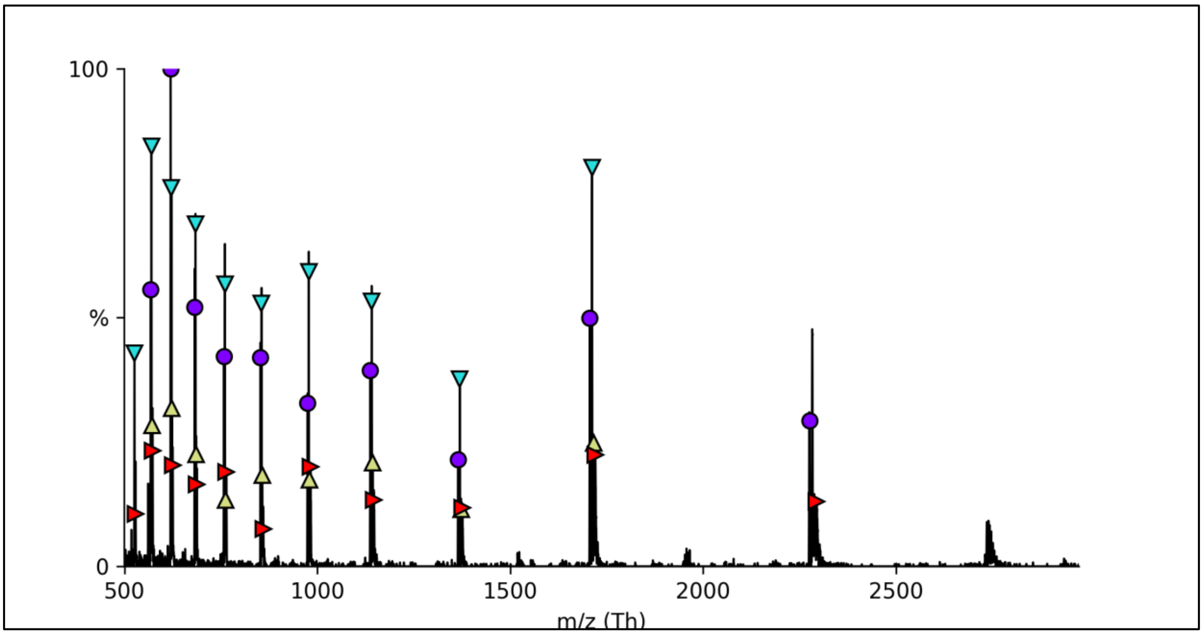

Figure S53. MS spectrum from LC-MS analysis of transcription reaction of **21RNA_3A-bind**. Raw mass spectrum.

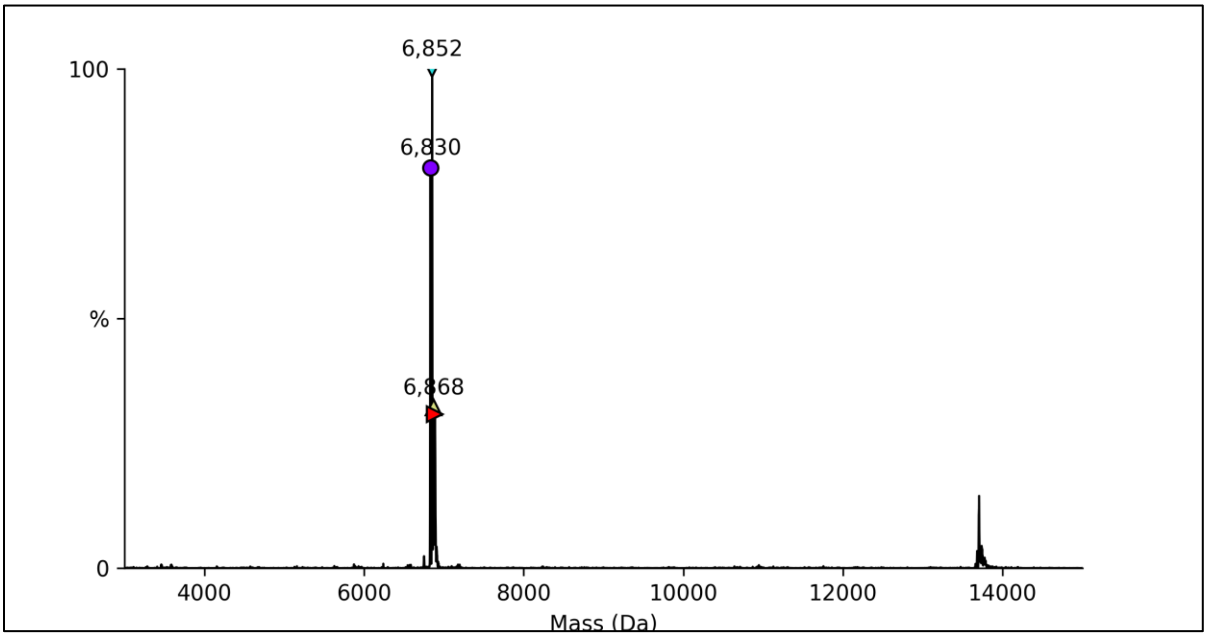

Figure S54. MS spectrum from LC-MS analysis of transcription reaction of **21RNA_3A-bind**. Deconvoluted mass spectrum. calculated: 6830.1 Da; found: 6830.0 Da; Δ = 0.1 Da. The peak at *m*/*z* = 6852.0 Da can be assigned to the adduct [**21RNA_3A-bind** + Na^+].

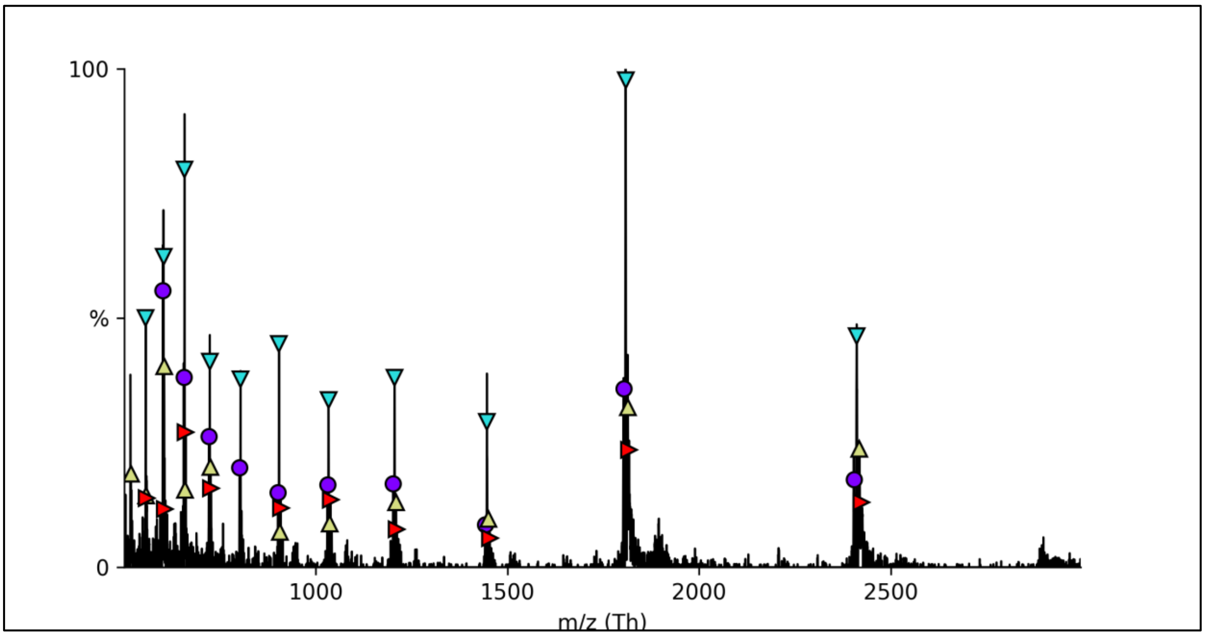

Figure S55. MS spectrum from LC-MS analysis of transcription reaction of **21RNA_3A^{CA}-bind**. Raw mass spectrum.

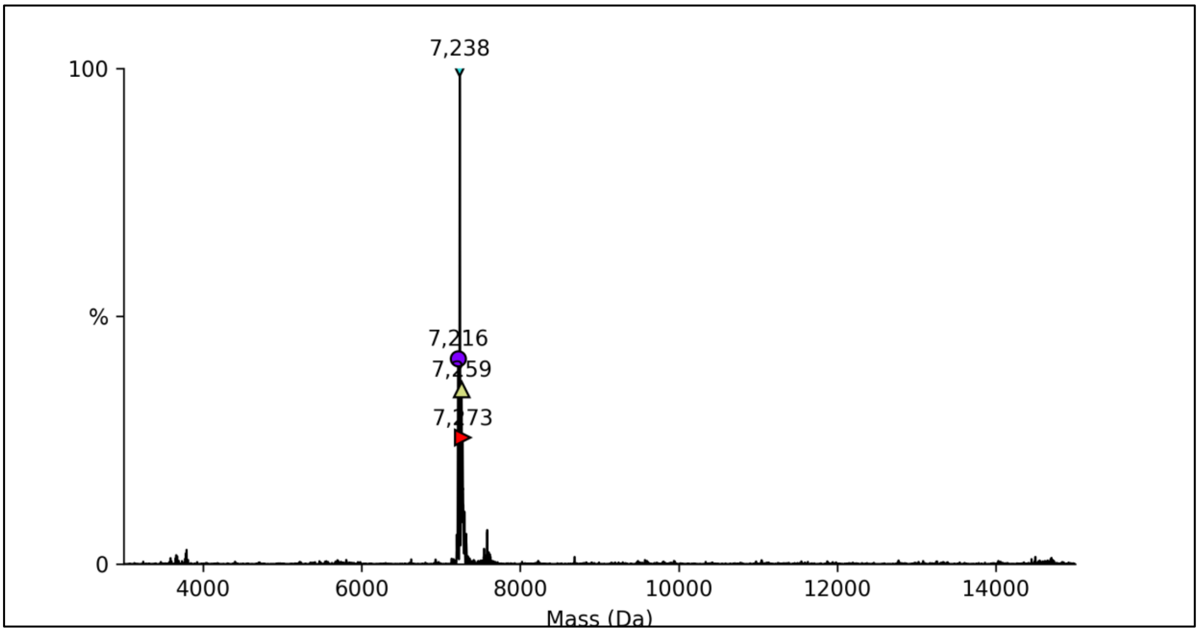

Figure S56. MS spectrum from LC-MS analysis of transcription reaction of **21RNA_3A^{CA}-bind**. Deconvoluted mass spectrum. calculated: 7216.4 Da; found: 7216.0 Da; Δ = 0.4 Da. The peak at *m*/*z* = 7238.0 Da can be assigned to the adduct [**21RNA_3A^{CA}-bind** + Na^+].

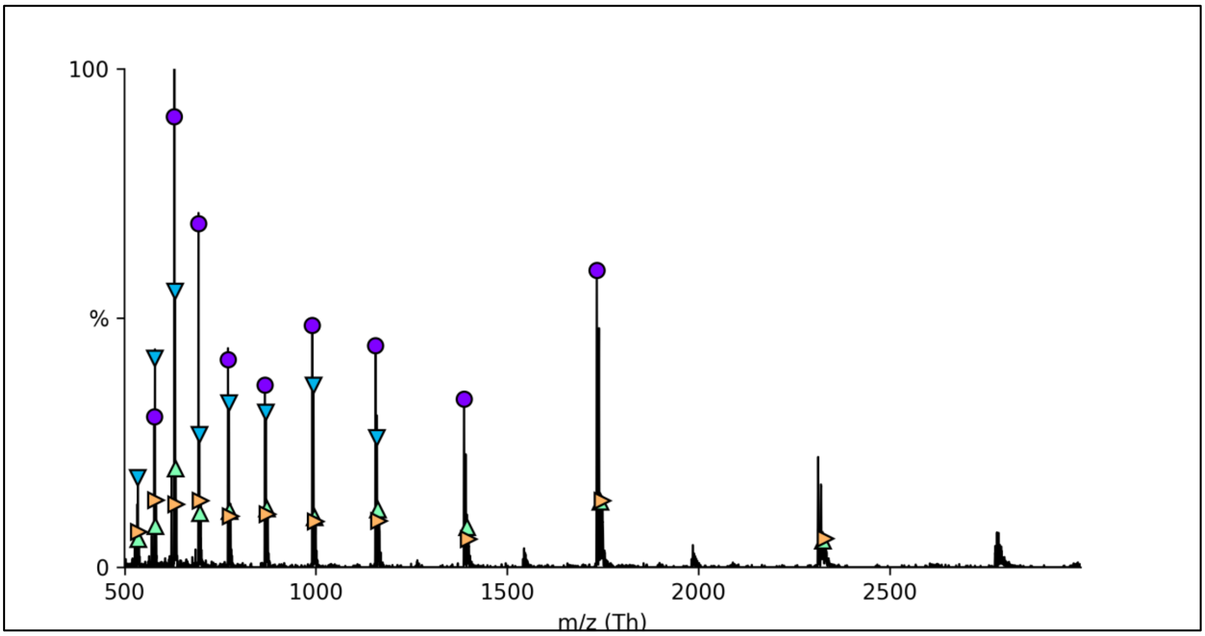

Figure S57. MS spectrum from LC-MS analysis of transcription reaction of **21RNA_3A-non-bind**. Raw mass spectrum.

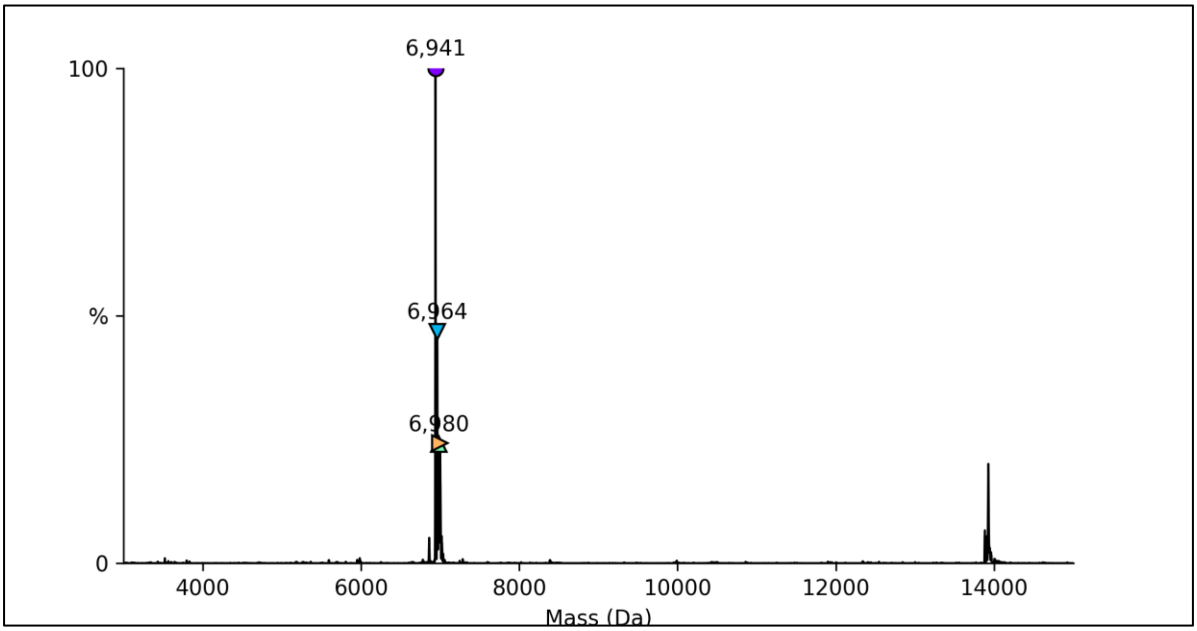

Figure S58. MS spectrum from LC-MS analysis of transcription reaction of **21RNA_3A-non-bind**. Deconvoluted mass spectrum. calculated: 6941.9 Da; found: 6941.0 Da; Δ = 0.9 Da. The peak at *m*/*z* = 6964.0 Da can be assigned to the adduct [**21RNA_3A-non-bind** + Na^+].

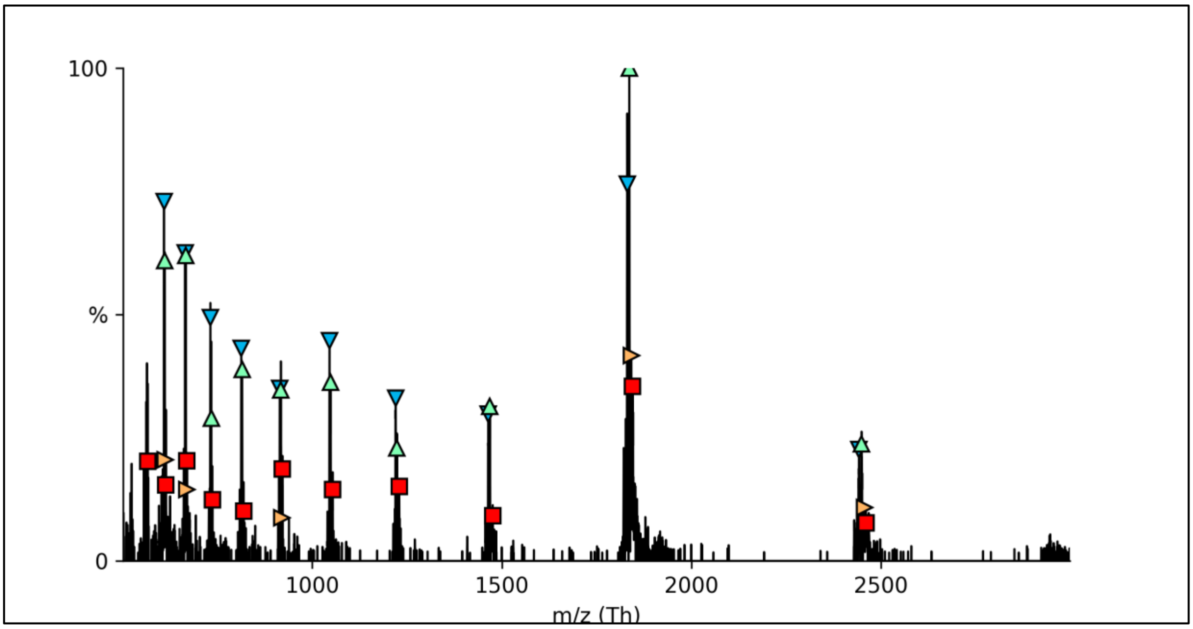

Figure S59. MS spectrum from LC-MS analysis of transcription reaction of **21RNA_3A^{CA}-non-bind**. Raw mass spectrum.

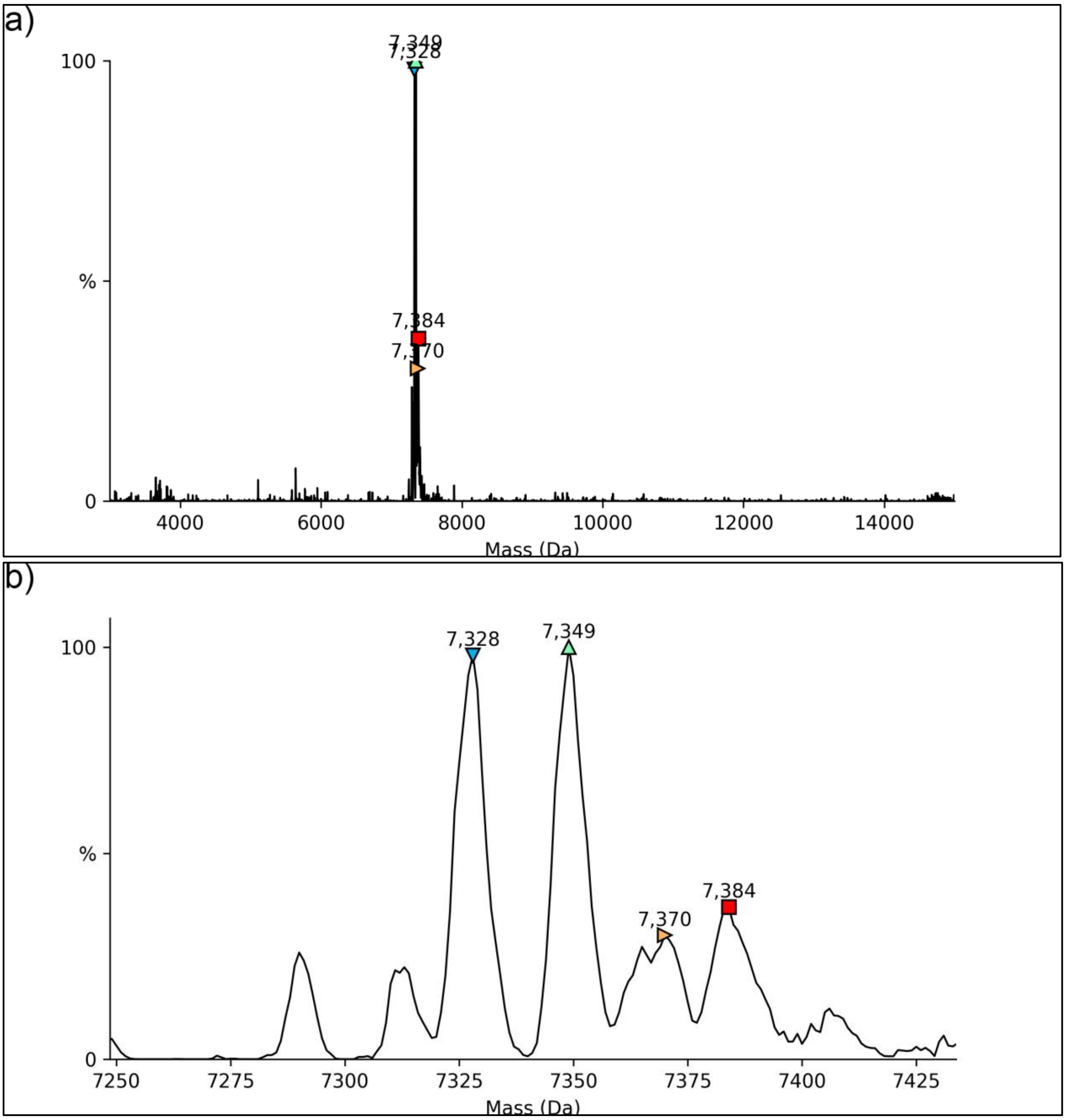

Figure S60. a) Full MS spectrum from LC-MS analysis of transcription reaction of **21RNA_3A^{CA}-non-bind**. Deconvoluted mass spectrum. b) Magnified area of interest. calculated: 7327.6 Da; found: 7328.0 Da; Δ = 0.4 Da. The peak at *m*/*z* = 7349.0 Da can be assigned to the adduct [**21RNA_3A^{CA}-non-bind** + Na^+].

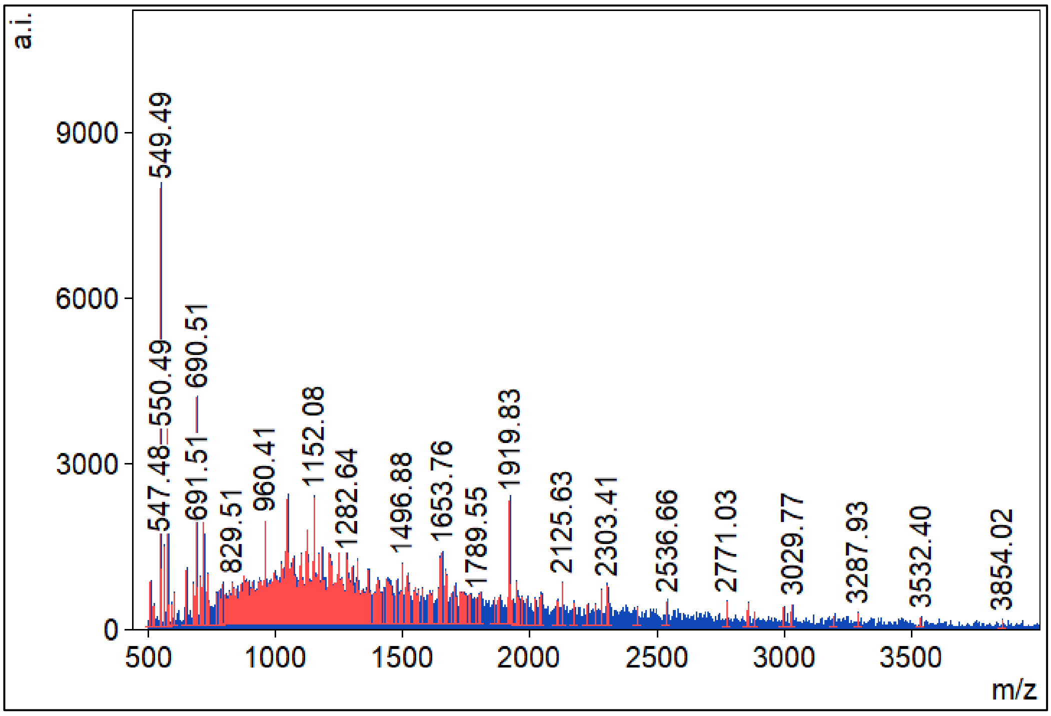

Figure S61. Raw mass spectrum of **20RNA_1A$^{\mathbf{CA\text{-}HuR}}$** conjugate.

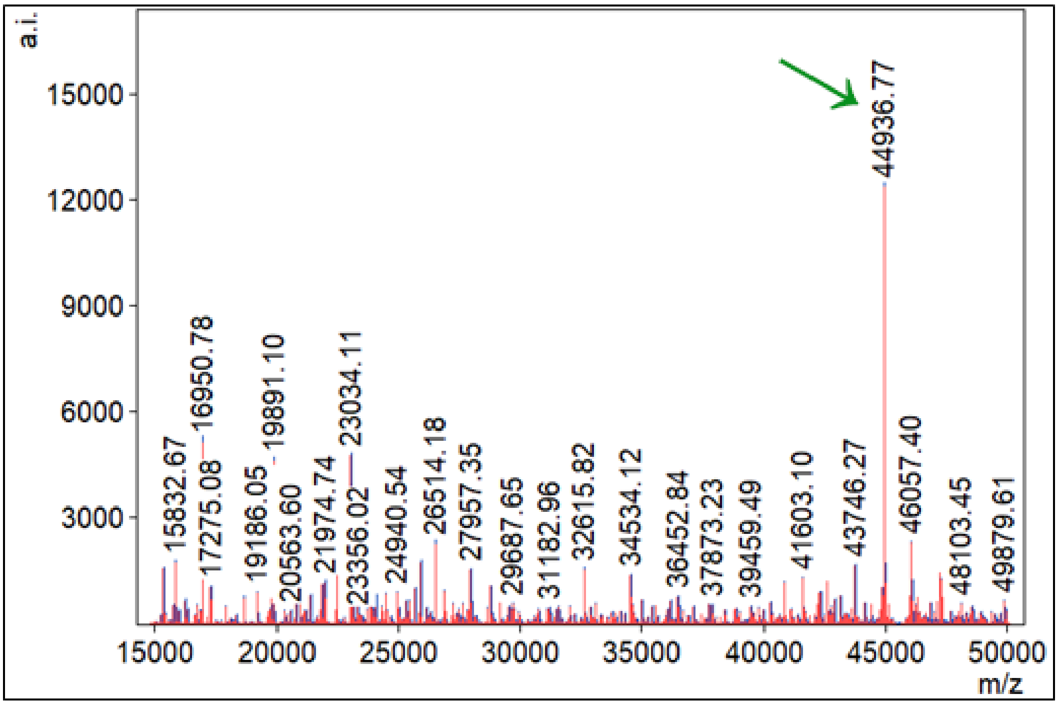

Figure S62. Deconvoluted mass spectrum of **20RNA_1A$^{\mathbf{CA\text{-}HuR}}$** conjugate; calculated: 44937.89 Da (with loss of N-terminal methionine); found: 44936.77 Da; Δ = 1.12 Da.

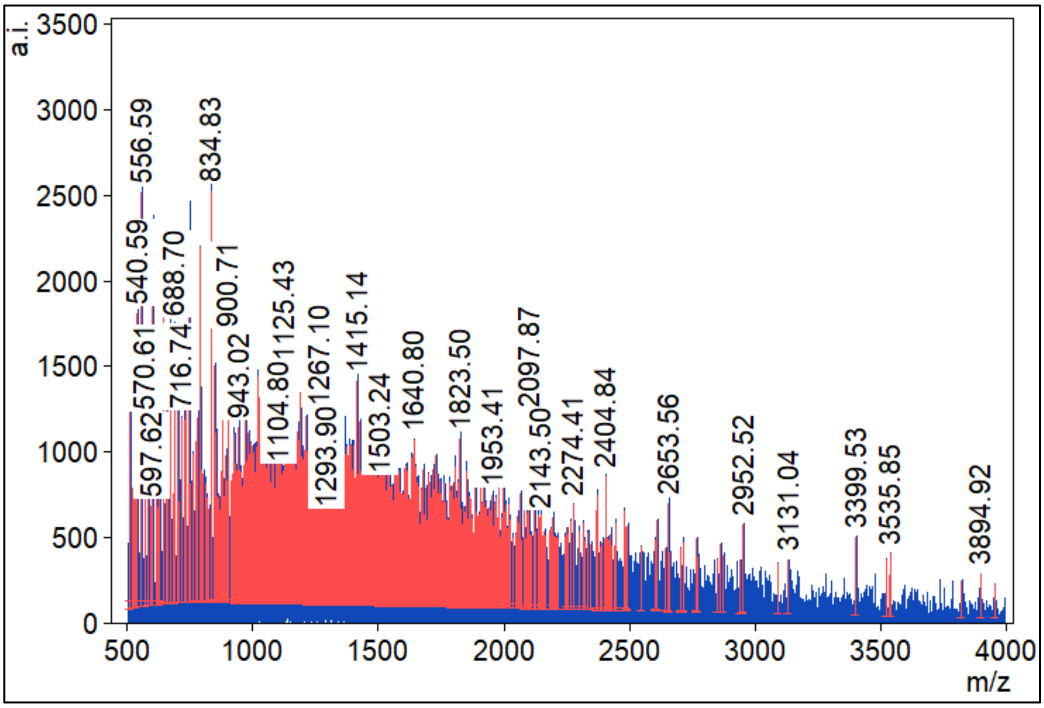

Figure S63. Raw mass spectrum of **20RNA_1A$^{\mathbf{CA\text{-}HIV\text{-}RT}}$** conjugate.

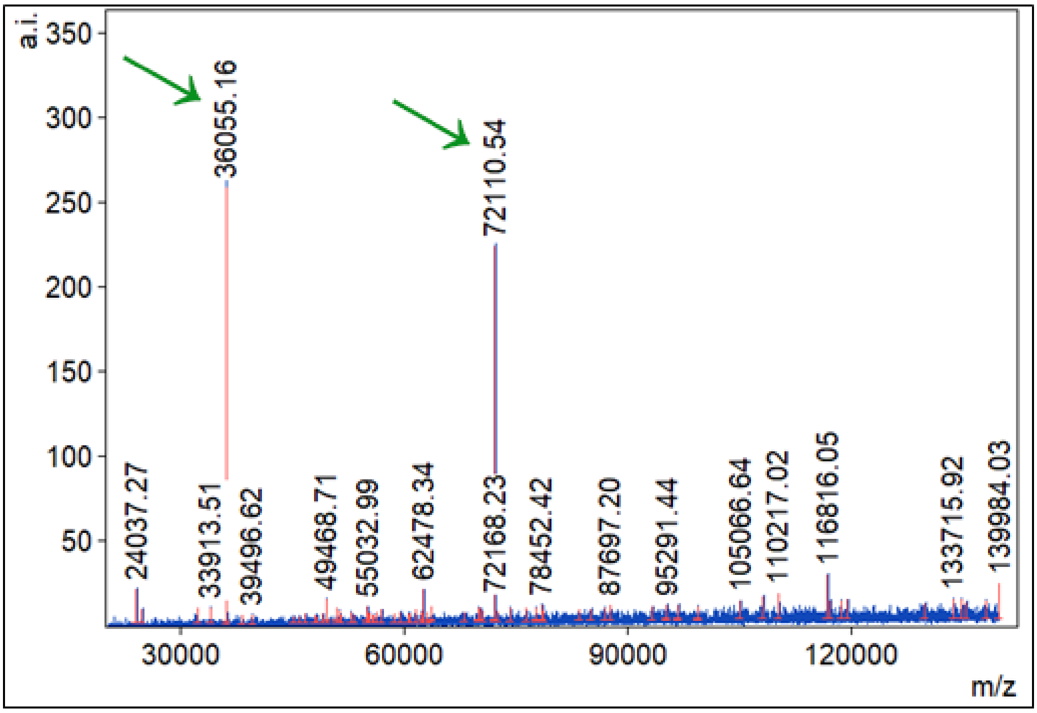

Figure S64. Deconvoluted mass spectrum of **20RNA_1A$^{\mathbf{CA\text{-}HIV\text{-}RT}}$** conjugate; calculated: 72094.0 Da; found: 72110.54 Da; $\Delta = 16.54$ Da. The peak at m/z = 36055.16 Da can be assigned to $[M+2H]^{2+}$ of **20RNA_1A$^{\mathbf{CA\text{-}HIV\text{-}RT}}$** conjugate.

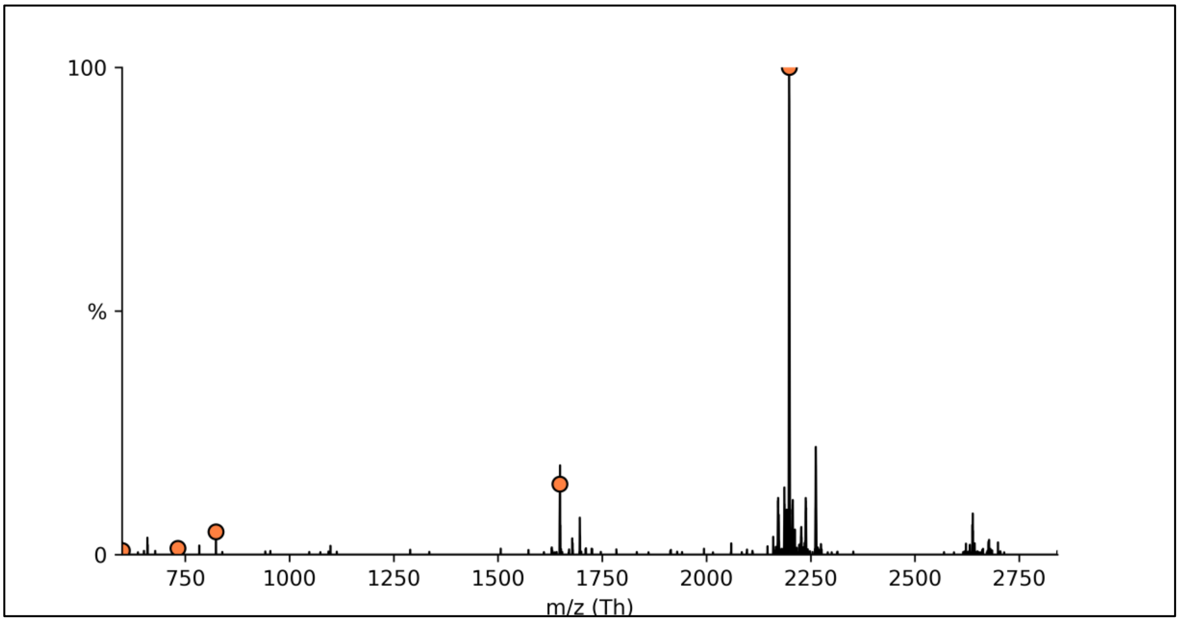

Figure S65. MS spectrum from LC-MS analysis of cross-linking of **20RNA_1A^{CA}** and HIV-RT protein. MS spectrum of peak 1 (free, unreacted RNA). Raw mass spectrum.

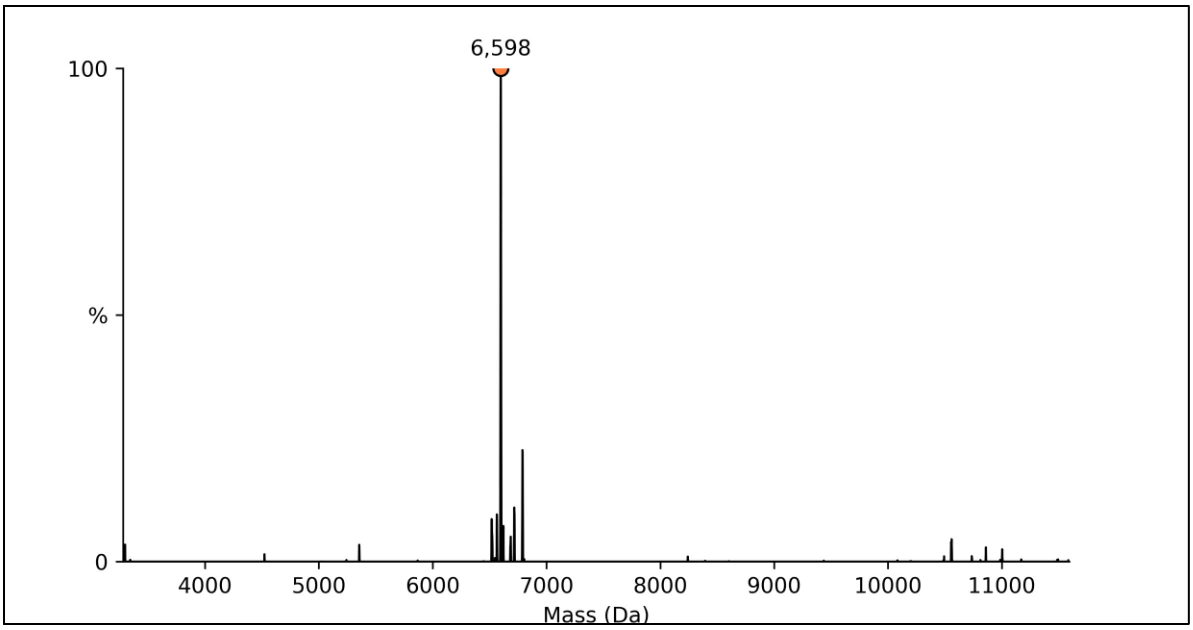

Figure S66. MS spectrum from LC-MS analysis of cross-linking of **20RNA_1A^{CA}** and HIV-RT protein. MS spectrum of peak 1 (free, unreacted RNA). Deconvoluted mass spectrum. calculated: 6601.2 Da; found: 6598.0 Da; Δ = 3.2 Da.

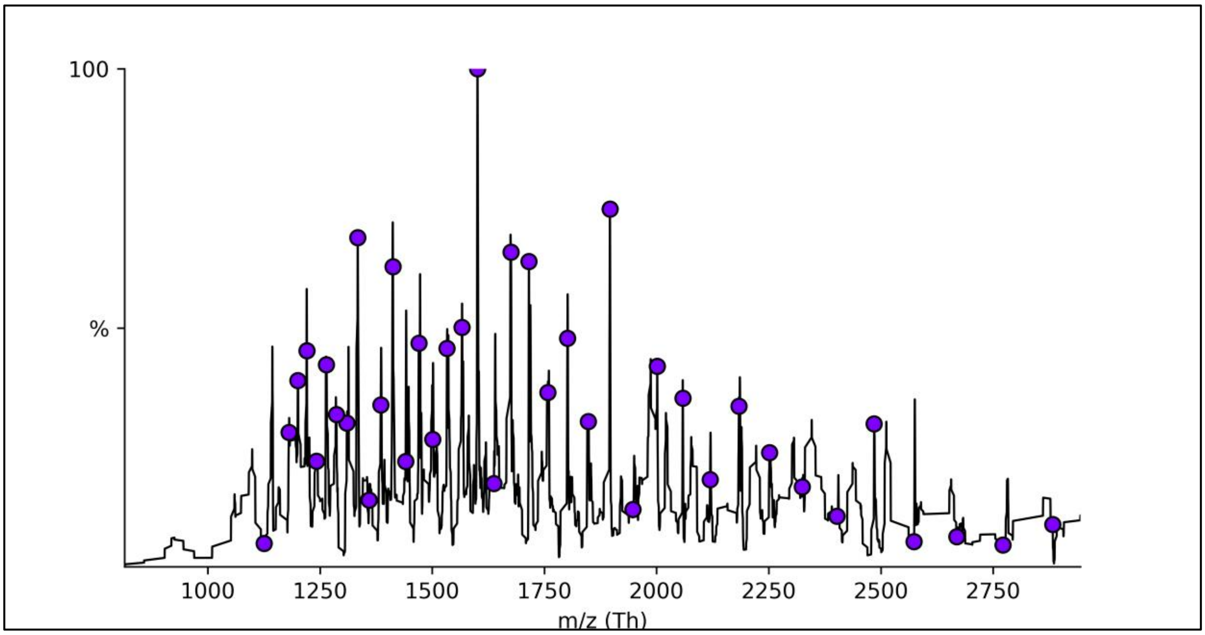

Figure S67. MS spectrum from LC-MS analysis of cross-linking of **20RNA_1A^{CA}** and HIV-RT protein. MS spectrum of peak 2 (**20RNA_1A$^{CA\text{-}HIV\text{-}RT}$** conjugate). Raw mass spectrum.

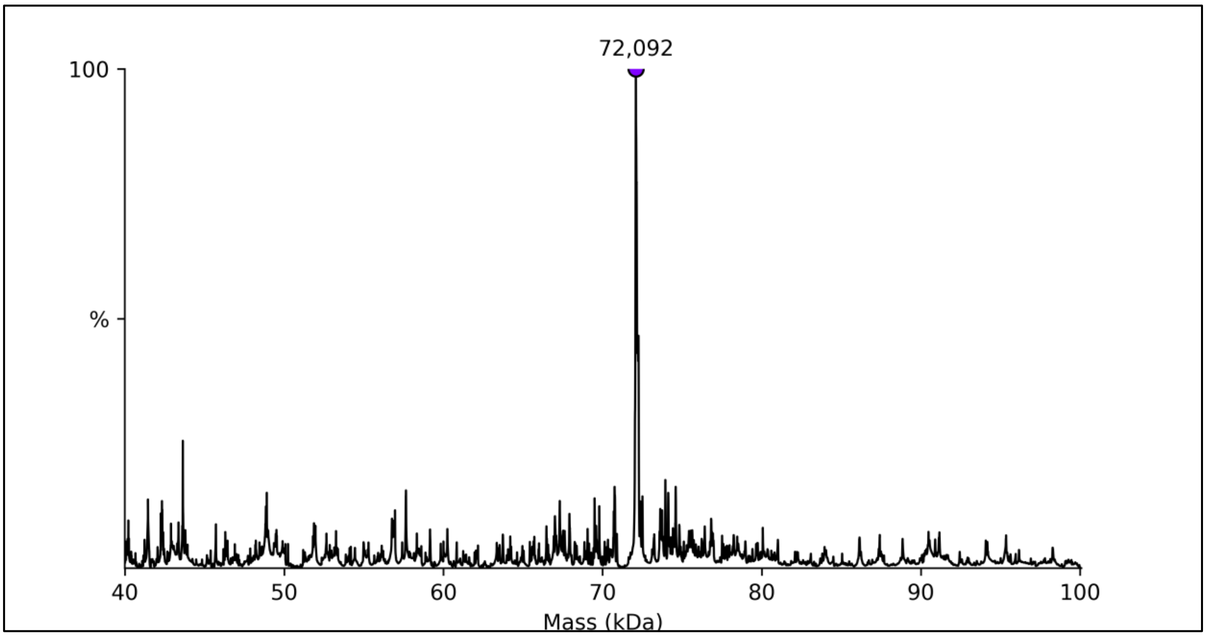

Figure S68. MS spectrum from LC-MS analysis of cross-linking of **20RNA_1A^{CA}** and HIV-RT protein. MS spectrum of peak 2 (**20RNA_1A$^{CA\text{-}HIV\text{-}RT}$** conjugate). Deconvoluted mass spectrum. calculated: 72094.0 Da; found: 72092.0 Da; Δ = 2.0 Da.

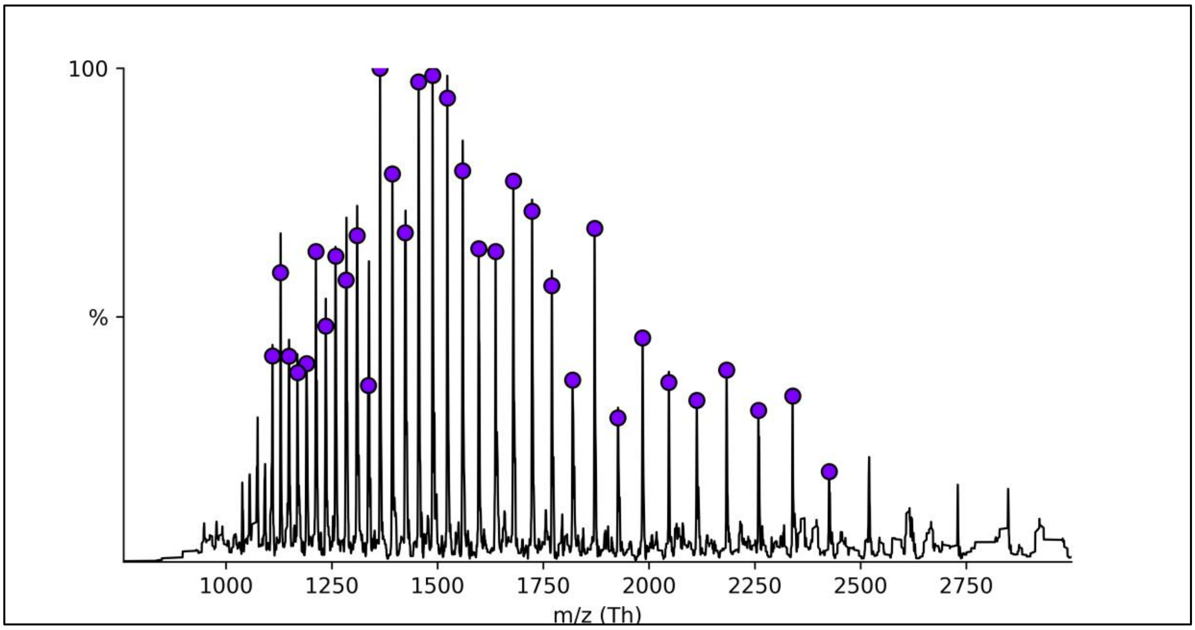

Figure S69. MS spectrum from LC-MS analysis of cross-linking of **20RNA_1A^{CA}** and HIV-RT protein. MS spectrum of peak 3 (free, unreacted protein). Raw mass spectrum.

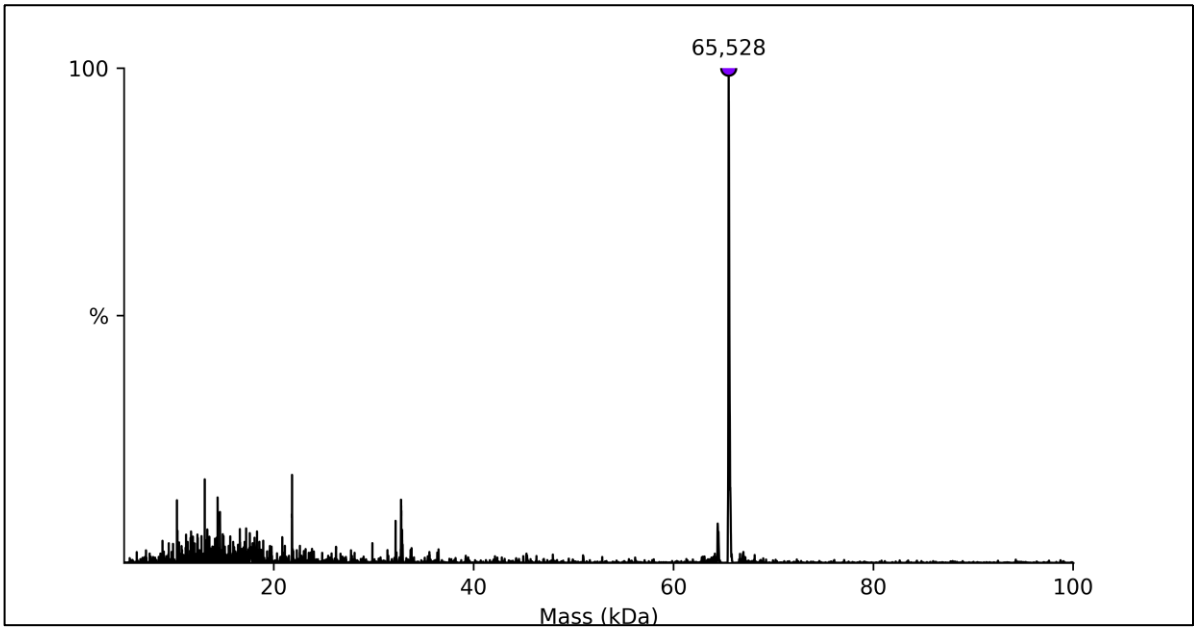

Figure S70. MS spectrum from LC-MS analysis of cross-linking of **20RNA_1A^{CA}** and HIV-RT protein. MS spectrum of peak 3 (free, unreacted protein). Deconvoluted mass spectrum. calculated: 65529.3 Da; found: 65528.0 Da; Δ = 1.3 Da.

3.3 Copies of nano-LC-MS/MS spectra

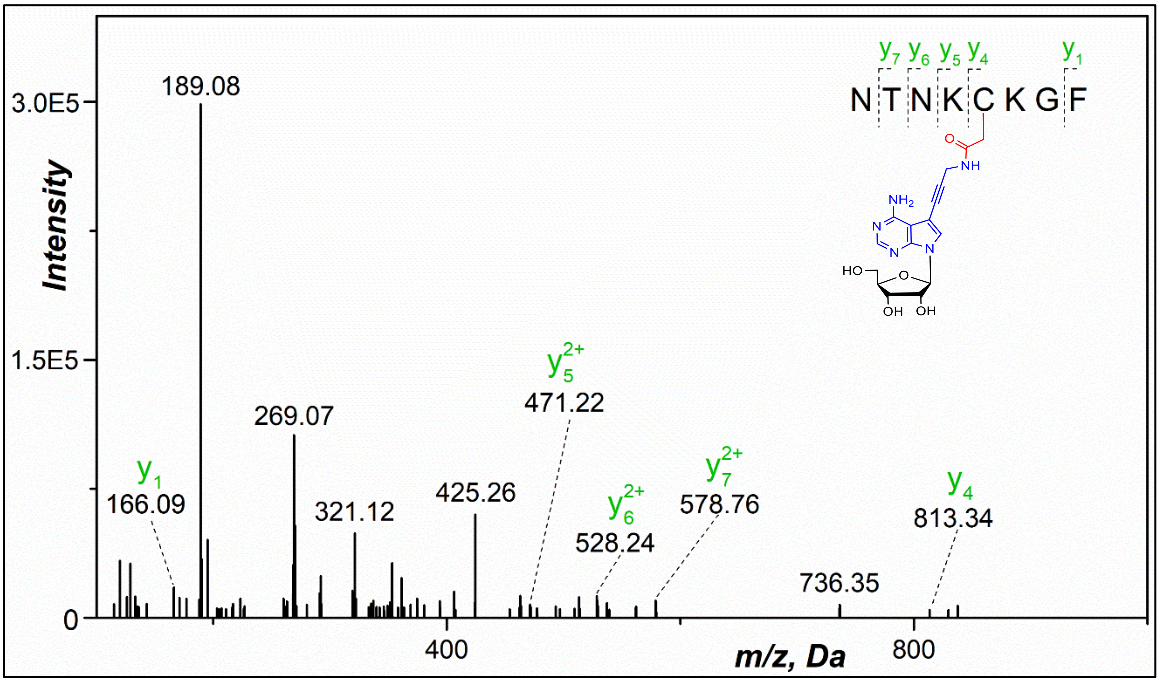

Figure S71. Nano-ESI$^+$-MS/MS spectrum of peptide from **20RNA_1A^{CA-HuR}** conjugate digest. Modification cross-linked to cysteine (position C306). *m/z* acquired: 424.1934 Da, z = 3+.

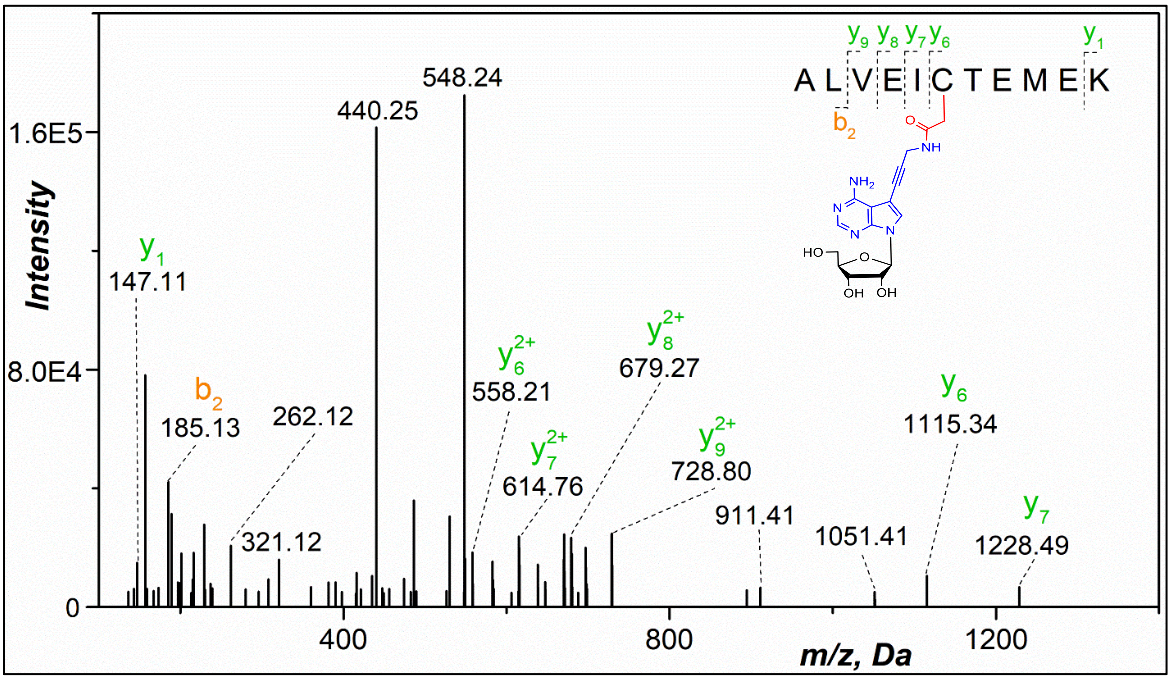

Figure S72. Nano-ESI$^+$-MS/MS spectrum of peptide from **20RNA_1A$^{CA-HIV-RT}$** conjugate digest. Modification cross-linked to cysteine (position C38). *m/z* acquired: 547.5820 Da, z = 3+.

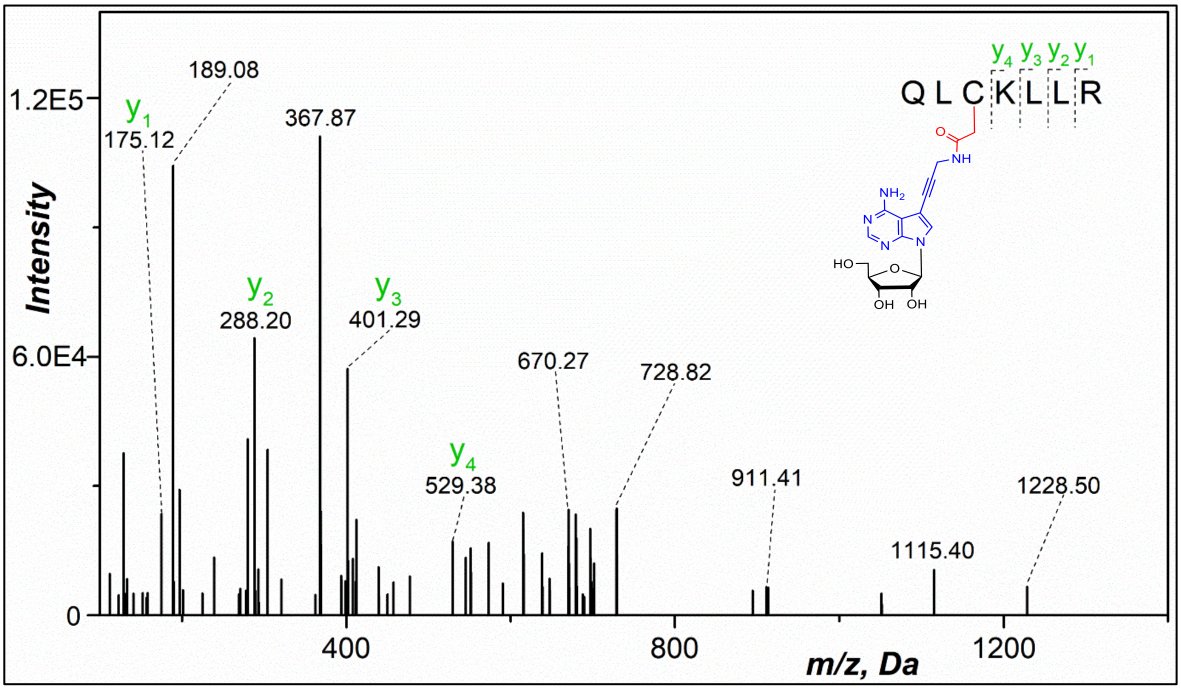

Figure S73. Nano-ESI$^+$-MS/MS spectrum of peptide from **20RNA_1A$^{CA\text{-}HIV\text{-}RT}$** conjugate digest. Modification cross-linked to cysteine (position C280). *m*/*z* acquired: 411.8861 Da, z = 3+.

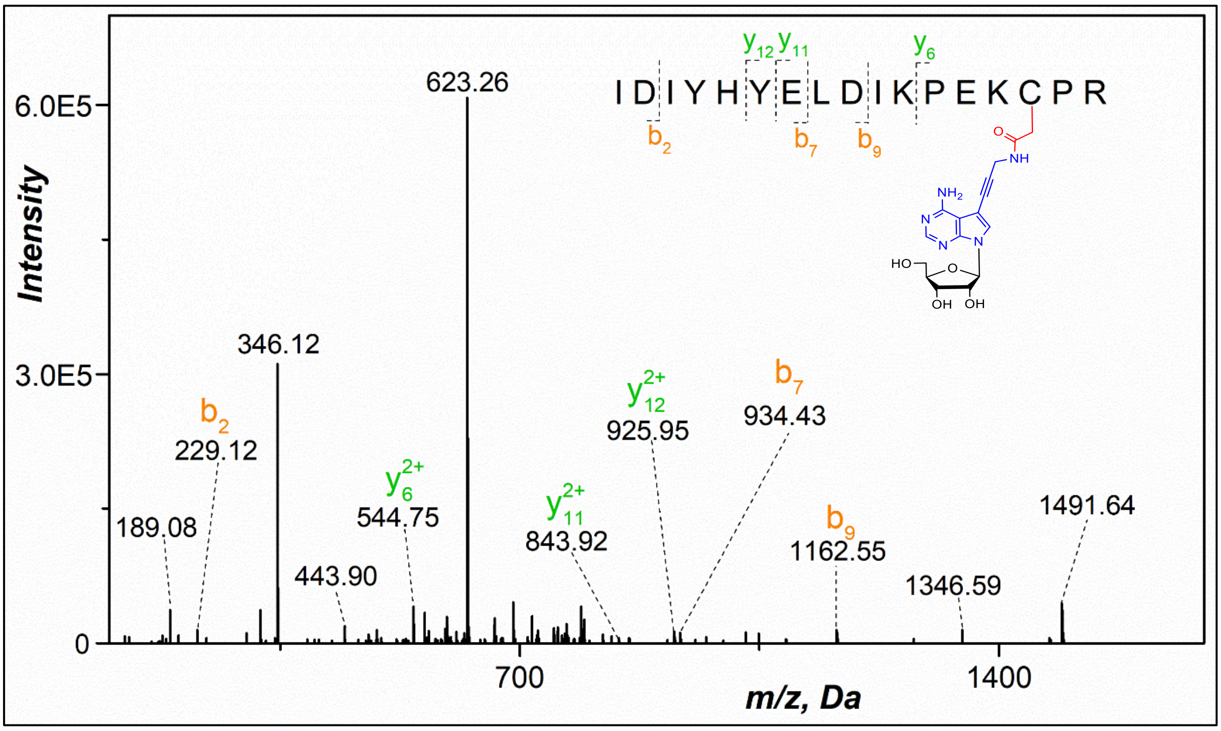

Figure S74. Nano-ESI$^+$-MS/MS spectrum of peptide from **20RNA_1A$^{CA\text{-}hAgo2}$** conjugate digest. Modification cross-linked to cysteine (position C84). *m*/*z* acquired: 623.5595 Da, z = 4+.

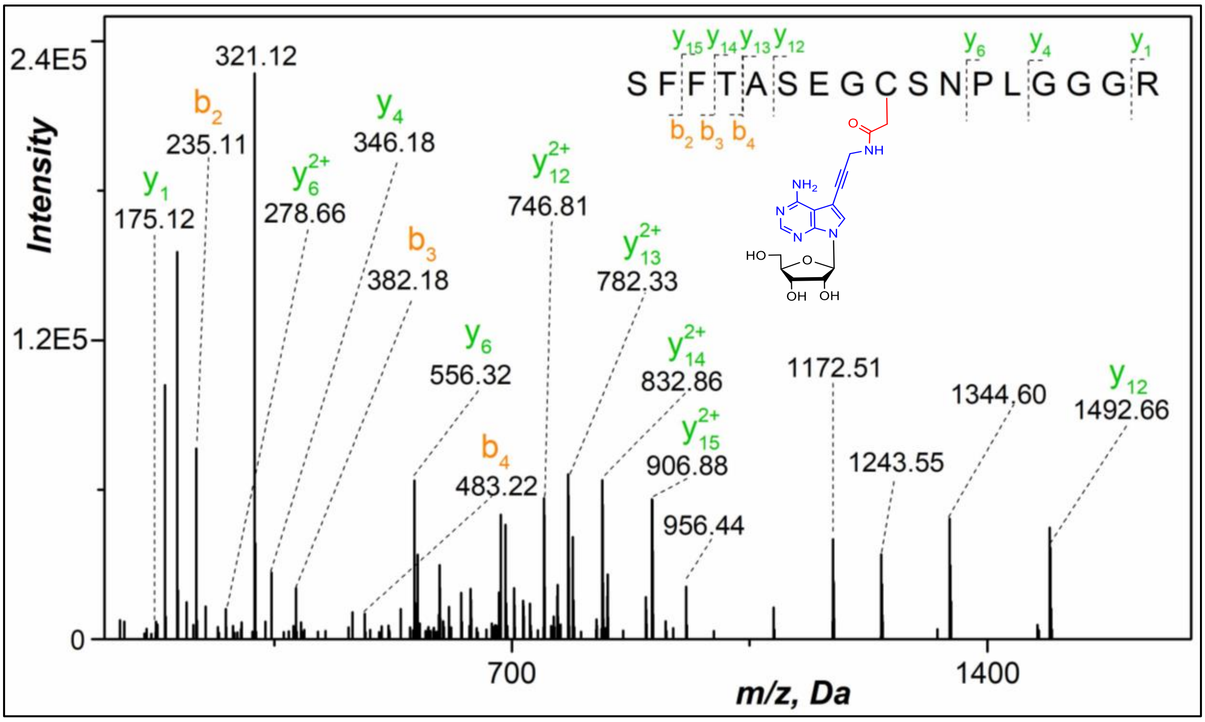

Figure S75. Nano-ESI$^+$-MS/MS spectrum of peptide from **20RNA_1A$^{CA\text{-}hAgo2}$** conjugate digest. Modification cross-linked to cysteine (position C206). *m/z* acquired: 682.6306 Da, z = 3+.

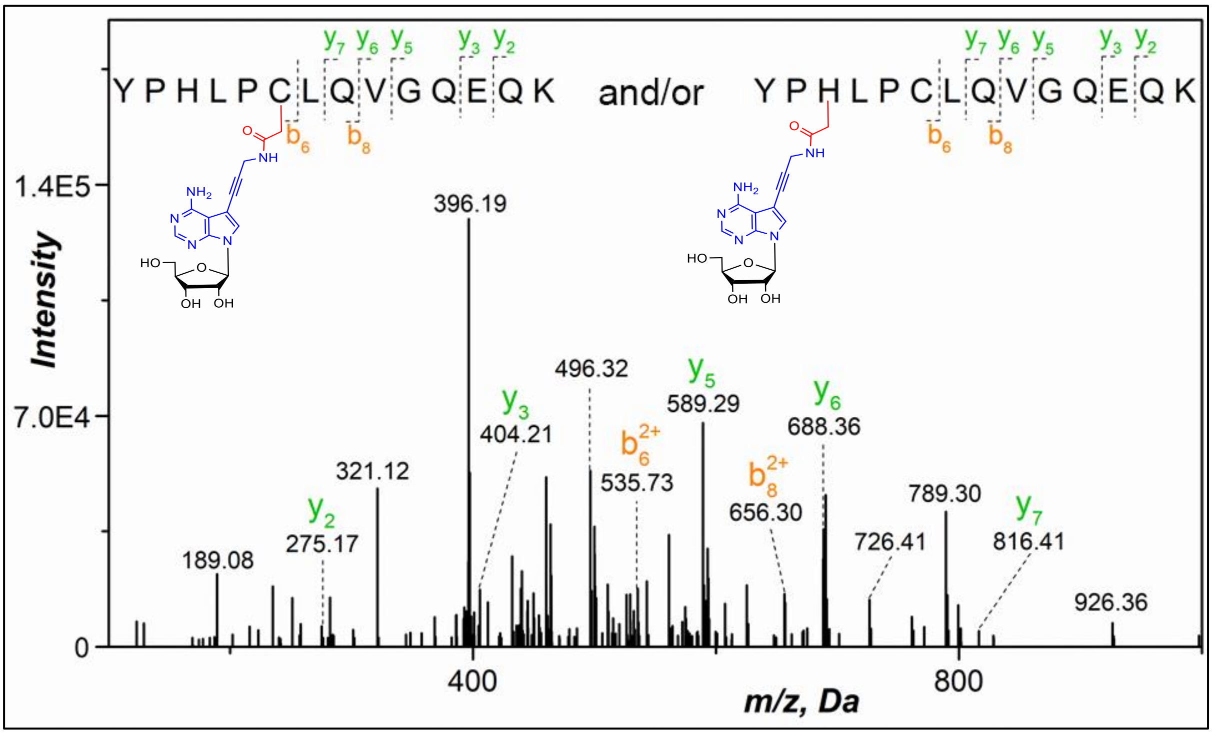

Figure S76. Nano-ESI$^+$-MS/MS spectrum of peptide from **20RNA_1A$^{CA\text{-}hAgo2}$** conjugate digest. Modification cross-linked to cysteine (position C345) and/or to histidine (position H342). *m/z* acquired: 500.4941 Da, z = 4+.

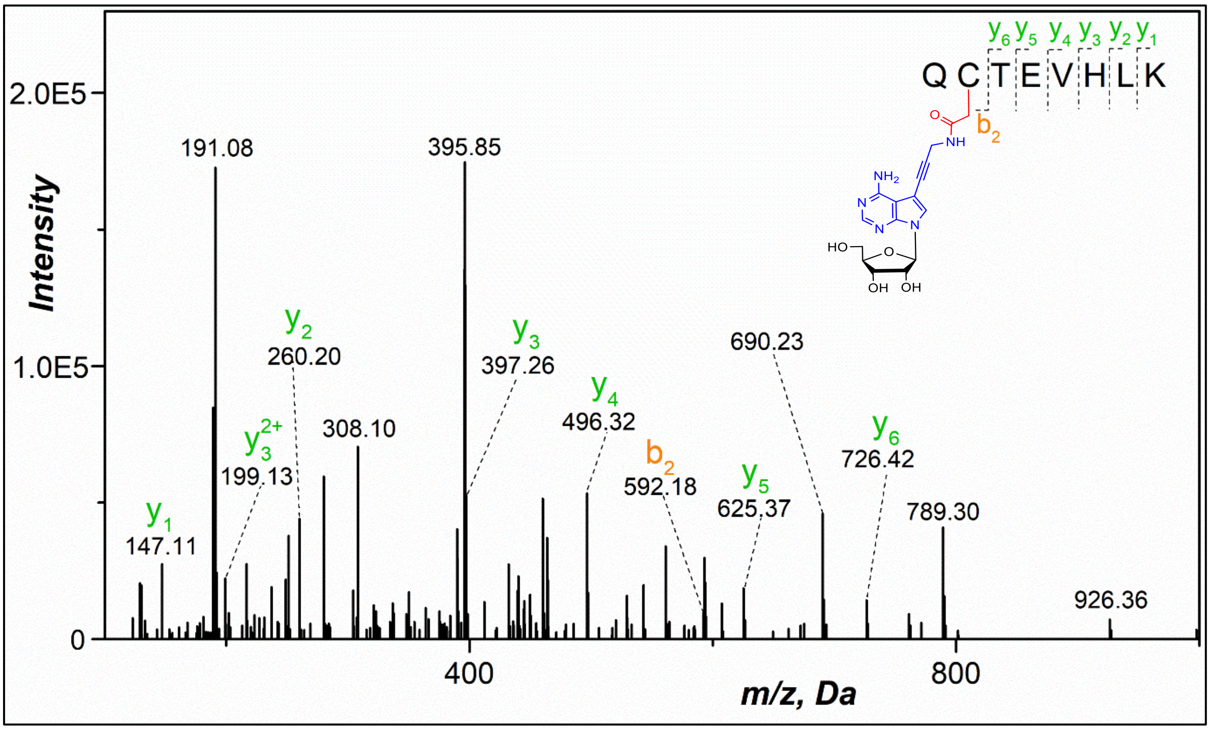

Figure S77. Nano-ESI$^+$-MS/MS spectrum of peptide from **20RNA_1A$^{\mathbf{CA\text{-}hAgo2}}$** conjugate digest. Modification cross-linked to cysteine (position C480). *m/z* acquired: 439.8680 Da, z = 3+.

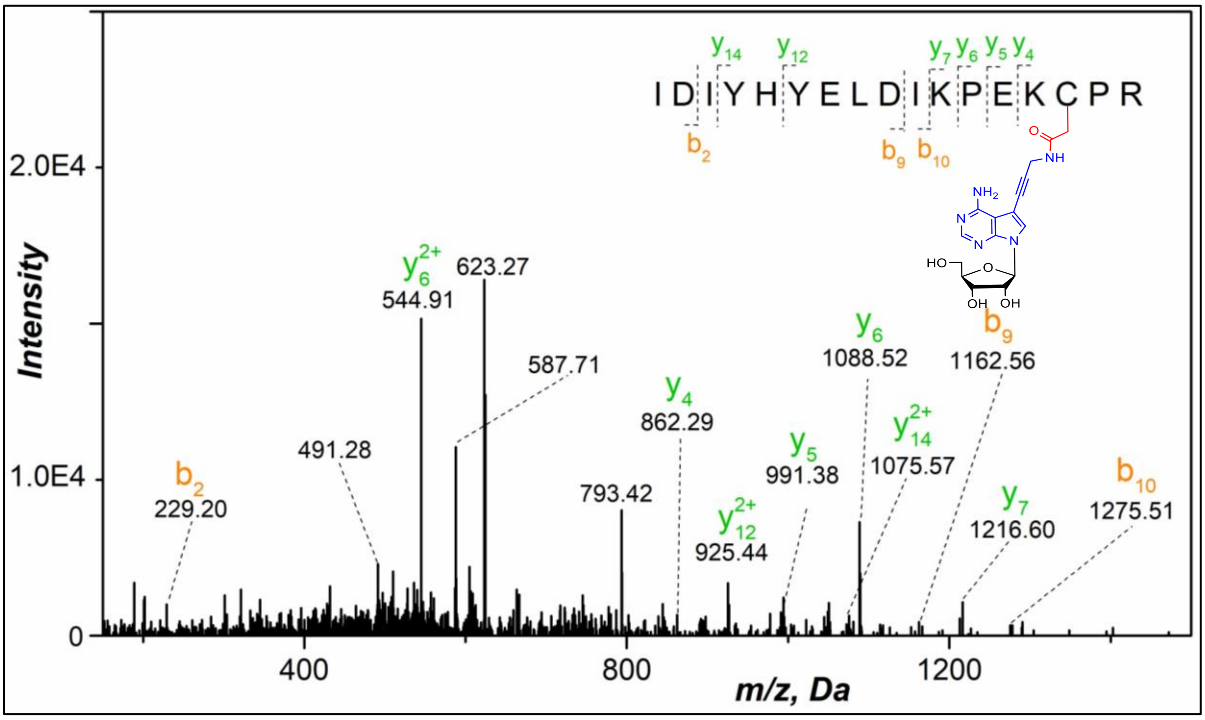

Figure S78. Nano-ESI$^+$-MS/MS spectrum of peptide from **20RNA_1A$^{\mathbf{CA\text{-}hAgo2}}$** conjugate digest. Modification cross-linked to cysteine (position C84). *m/z* acquired: 623.5577 Da, z = 4+.

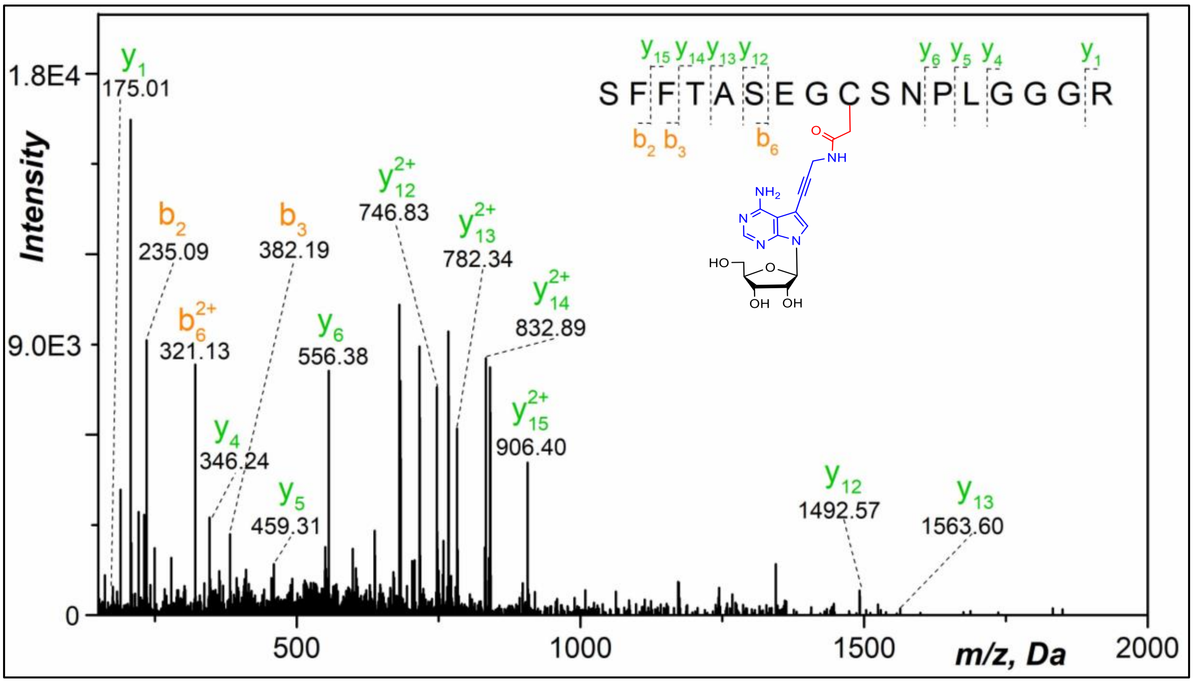

Figure S79. Nano-ESI$^+$-MS/MS spectrum of peptide from **20RNA_1A$^{\textbf{CA-hAgo2}}$** conjugate digest. Modification cross-linked to cysteine (position C206). *m*/*z* acquired: 682.6306 Da, z = 3+.

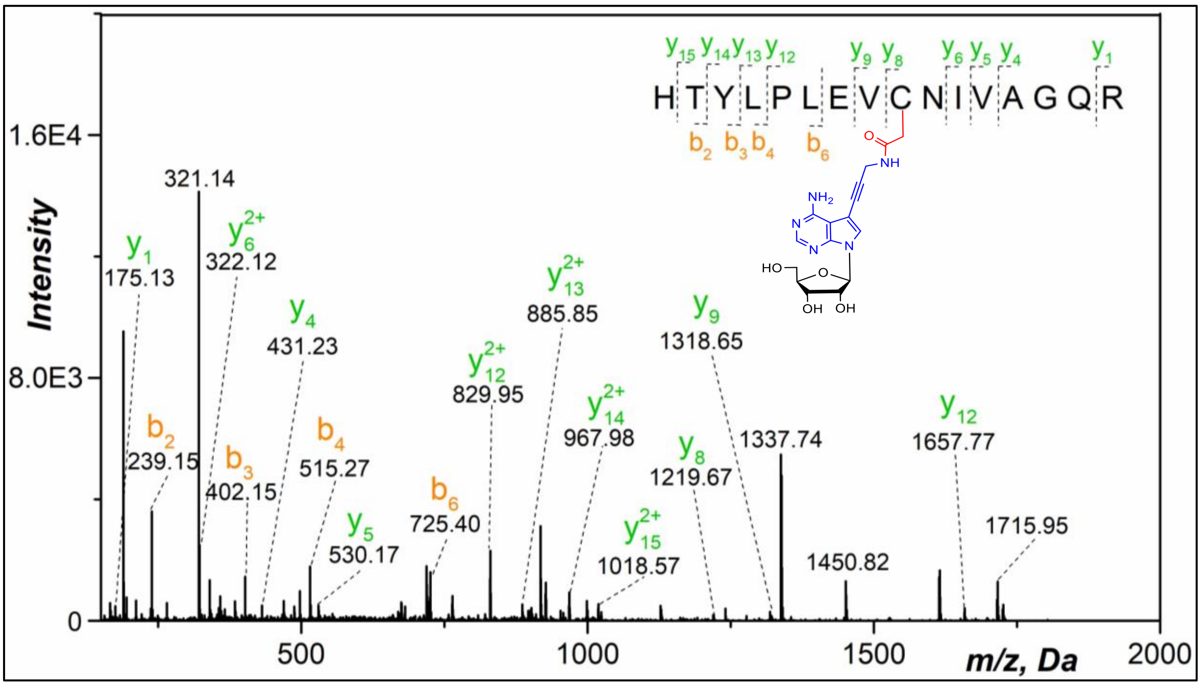

Figure S80. Nano-ESI$^+$-MS/MS spectrum of peptide from **20RNA_1A$^{\textbf{CA-hAgo2}}$** conjugate digest. Modification cross-linked to cysteine (position C362). *m*/*z* acquired: 724.6937 Da, z = 3+.

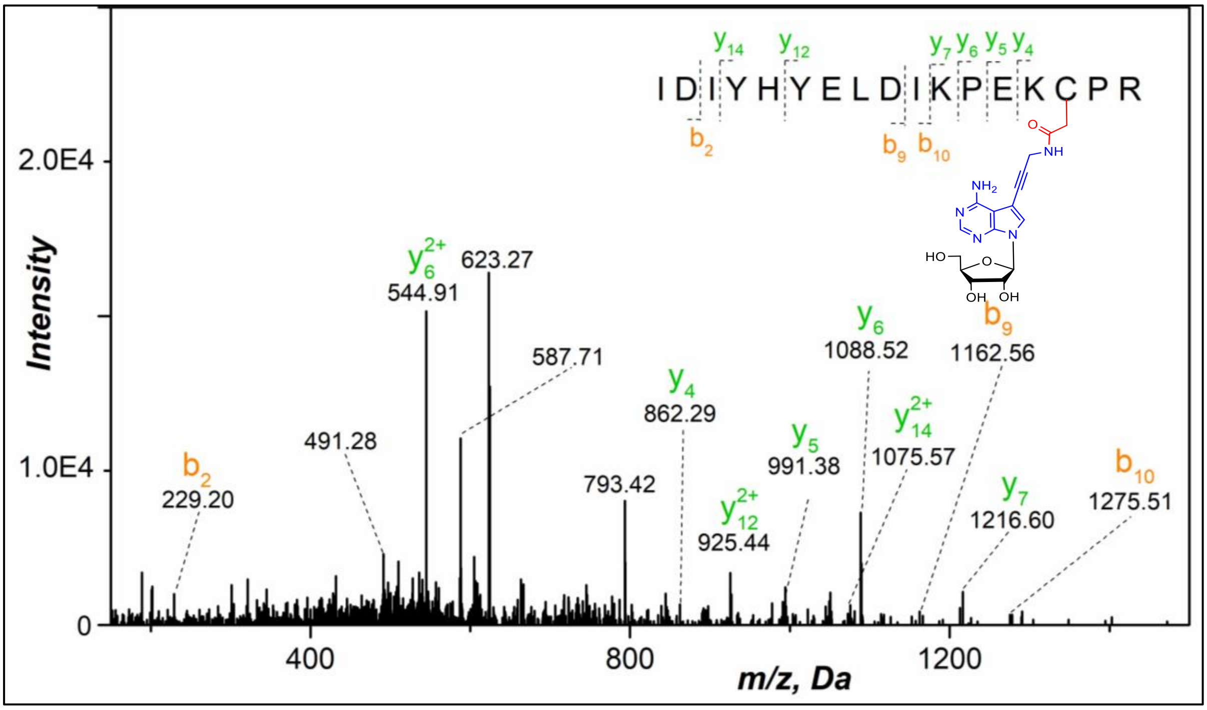

Figure S81. Nano-ESI+-MS/MS spectrum of peptide from **20RNA_1A$^{\mathbf{CA\text{-}hAgo2}}$** conjugate digest. Modification cross-linked to cysteine (position C84). *m*/*z* acquired: 623.5577 Da, z = 4+.

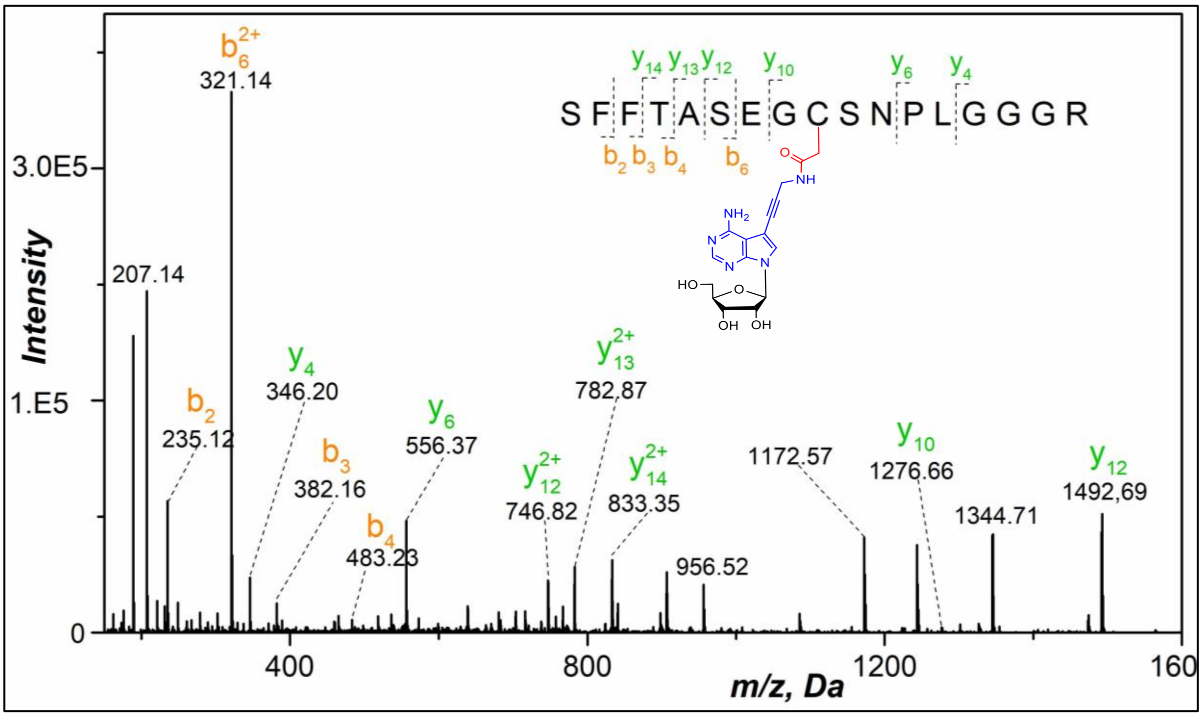

Figure S82. Nano-ESI+-MS/MS spectrum of peptide from **20RNA_1A$^{\mathbf{CA\text{-}hAgo2}}$** conjugate digest. Modification cross-linked to cysteine (position C206). *m*/*z* acquired: 682.6306 Da, z = 3+.

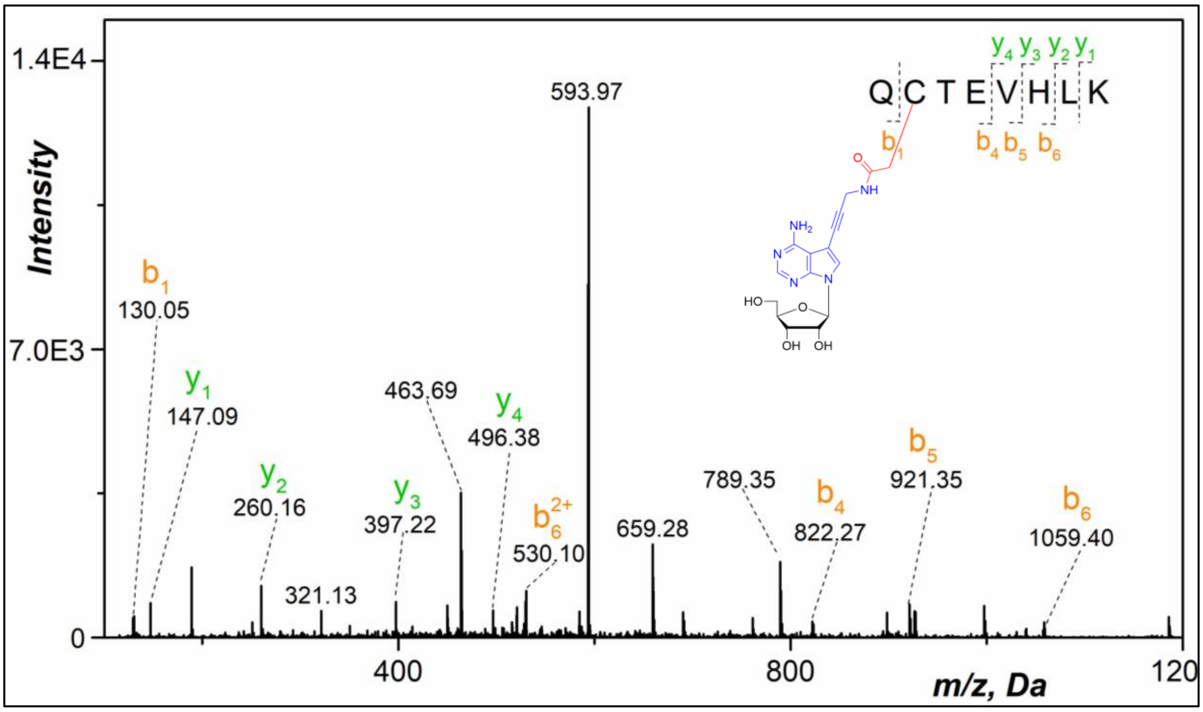

Figure S83. Nano-ESI$^+$-MS/MS spectrum of peptide from **20RNA_1A$^{\text{CA-hAgo2}}$** conjugate digest. Modification cross-linked to cysteine (position C480). *m*/*z* acquired: 659.2984 Da, z = 2+.

4 Additional information

4.1 Protein sequence of human antigen R protein (HuR) used in this study

```
        10         20         30         40         50         60
MGSSHHHHHH SSGENLYFQG SGMSNGYEDH MAEDCRGDIG RTNLIVNYLP QNMTQDELRS

        70         80         90        100        110        120
LFSSIGEVES AKLIRDKVAG HSLGYGFVNY VTAKDAERAI NTLNGLRLQS KTIKVSYARP

       130        140        150        160        170        180
SSEVIKDANL YISGLPRTMT QKDVEDMFSR FGRIINSRVL VDQTTGLSRG VAFIRFDKRS

       190        200        210        220        230        240
EAEEAITSFN GHKPPGSSEP ITVKFAANPN QNKNVALLSQ LYHSPARRFG GPVHHQAQRF

       250        260        270        280        290        300
RFSPMGVDHM SGLSGVNVPG NASSGWCIFI YNLGQDADEG ILWQMFGPFG AVTNVKVIRD

       310        320        330        340
FNTNKCKGFG FVTMTNYEEA AMAIASLNGY RLGDKILQVS FKTNKSHK
```

All cysteines are highlighted in green and histidines are highlighted in yellow. Cross-linked cysteine is highlighted in red.

4.2 Protein sequence of HIV reverse-transcriptase (HIV-RT) used in this study

Large subunit

```
        10         20         30         40         50         60
PISPIETVPV KLKPGMDGPK VKQWPLTEEK IKALVEICTE MEKEGKISKI GPENPYNTPV

        70         80         90        100        110        120
FAIKKKDSTK WRKLVDFREL NKRTQDFWEV QLGIPHPAGL KKKKSVTVLD VGDAYFSVPL

       130        140        150        160        170        180
DEDFRKYTAF TIPSINNETP GIRYQYNVLP QGWKGSPAIF QSSMTKILEP FKKQNPDIVI

       190        200        210        220        230        240
YQYMDDLYVG SDLEIGQHRT KIEELRQHLL RWGLTTPDKK HQKEPPFLWM GYELHPDKWT

       250        260        270        280        290        300
VQPIVLPEKD SWTVNDIQKL VGKLNWASQI YPGIKVRQLC KLLRGTKALT EVIPLTEEAE

       310        320        330        340        350        360
LELAENREIL KEPVHGVYYD PSKDLIAEIQ KQGQGQWTYQ IYQEPFKNLK TGKYARMRGA

       370        380        390        400        410        420
HTNDVKQLTE AVQKITTESI VIWGKTPKFK LPIQKETWET WWTEYWQATW IPEWEFVNTP

       430        440        450        460        470        480
PLVKLWYQLE KEPIVGAETF YVDGAANRET KLGKAGYVTN KGRQKVVPLT NTTNQKTELQ

       490        500        510        520        530        540
AIYLALQDSG LEVNIVTDSQ YALGIIQAQP DKSESELVNQ IIEQLIKKEK VYLAWVPAHK
```

```
       550        560        570
GIGGNEQVDK LVSAGIRNIL GSGSHHHHHH
```

Small subunit

```
        10         20         30         40         50         60
PISPIETVPV KLKPGMDGPK VKQWPLTEEK IKALVEICTE MEKEGKISKI GPENPYNTPV

        70         80         90        100        110        120
FAIKKKDSTK WRKLVDFREL NKRTQDFWEV QLGIPHPAGL KKKKSVTVLD VGDAYFSVPL

       130        140        150        160        170        180
DEDFRKYTAF TIPSINNETP GIRYQYNVLP QGWKGSPAIF QSSMTKILEP FKKQNPDIVI

       190        200        210        220        230        240
YQYMDDLYVG SDLEIGQHRT KIEELRQHLL RWGLTTPDKK HQKEPPFLWM GYELHPDKWT

       250        260        270        280        290        300
VQPIVLPEKD SWTVNDIQKL VGKLNWASQI YPGIKVRQLC KLLRGTKALT EVIPLTEEAE

       310        320        330        340        350        360
LELAENREIL KEPVHGVYYD PSKDLIAEIQ KQGQGQWTYQ IYQEPFKNLK TGKYARMRGA

       370        380        390        400        410        420
HTNDVKQLTE AVQKITTESI VIWGKTPKFK LPIQKETWET WWTEYWQATW IPEWEFVNTP

       430        440
PLVKLWYQLE KEPIVGAETF
```

All histidines are highlighted in yellow. Cross-linked cysteines are highlighted in red.

4.3 Protein sequence of human argonaute 2 protein (hAgo2) used in this study

```
        10         20         30         40         50         60
HHHHHHHHHH LEVLFQGPMY SGAGPALAPP APPPPIQGYA FKPPPRPDFG TSGRTIKLQA

        70         80         90        100        110        120
NFFEMDIPKI DIYHYELDIK PEKCPRRVNR EIVEHMVQHF KTQIFGDRKP VFDGRKNLYT

       130        140        150        160        170        180
AMPLPIGRDK VELEVTLPGE GKDRIFKVSI KWVSCVSLQA LHDALSGRLP SVPFETIQAL

       190        200        210        220        230        240
DVVMRHLPSM RYTPVGRSFF TASEGCSNPL GGGREVWFGF HQSVRPSLWK MMLNIDVSAT

       250        260        270        280        290        300
AFYKAQPVIE FVCEVLDFKS IEEQQKPLTD SQRVKFTKEI KGLKVEITHC GQMKRKYRVC

       310        320        330        340        350        360
NVTRRPASHQ TFPLQQESGQ TVECTVAQYF KDRHKLVLRY PHLPCLQVGQ EQKHTYLPLE

       370        380        390        400        410        420
VCNIVAGQRC IKKLTDNQTS TMIRATARSA PDRQEEISKL MRSASFNTDP YVREFGIMVK

       430        440        450        460        470        480
DEMTDVTGRV LQPPSILYGG RNKAIATPVQ GVWDMRNKQF HTGIEIKVWA IACFAPQRQC

       490        500        510        520        530        540
TEVHLKSFTE QLRKISRDAG MPIQGQPCFC KYAQGADSVE PMFRHLKNTY AGLQLVVVIL
```

```
       550        560        570        580        590        600
PGKTPVYAEV KRVGDTVLGM ATQCVQMKNV QRTTPQTLSN LCLKINVKLG GVNNILLPQG

       610        620        630        640        650        660
RPPVFQQPVI FLGADVTHPP AGDGKKPSIA AVVGSMDAHP NRYCATVRVQ QHRQEIIQDL

       670        680        690        700        710        720
AAMVRELLIQ FYKSTRFKPT RIIFYRDGVS EGQFQQVLHH ELLAIREACI KLEKDYQPGI

       730        740        750        760        770        780
TFIVVQKRHH TRLFCTDKNE RVGKSGNIPA GTTVDTKITH PTEFDFYLCS HAGIQGTSRP

       790        800        810        820        830        840
SHYHVLWDDN RFSSDELQIL TYQLCHTYVR CTRSVSIPAP AYYAHLVAFR ARYHLVDKEH

       850        860        870
DSAEGSHTSG QSNGRDHQAL AKAVQVHQDT LRTMYFA
```

All cysteines are highlighted in green and histidines are highlighted in yellow. Cross-linked cysteines and/or histidine are highlighted in red.

4.4 Protein sequence of galectin 1 protein (Gal1) used in this study

```
        10         20         30         40         50         60
ACGLVASNLN LKPGECLRVR GEVAPDAKSF VLNLGKDSNN LCLHFNPRFN AHGDANTIVC

        70         80         90        100        110        120
NSKDGGAWGT EQREAVFPFQ PGSVAEVCIT FDQANLTVKL PDGYEFKFPN RLNLEAINYM

       130        140   Bio   150
AADGDFKIKC VAFDGLNDIF EAQKIEWHGH HHHHH
```

All cysteines are highlighted in green and histidines are highlighted in yellow.
Bio (biotin).

4.5 Mapping of crosslinked amino acids on crystal structures

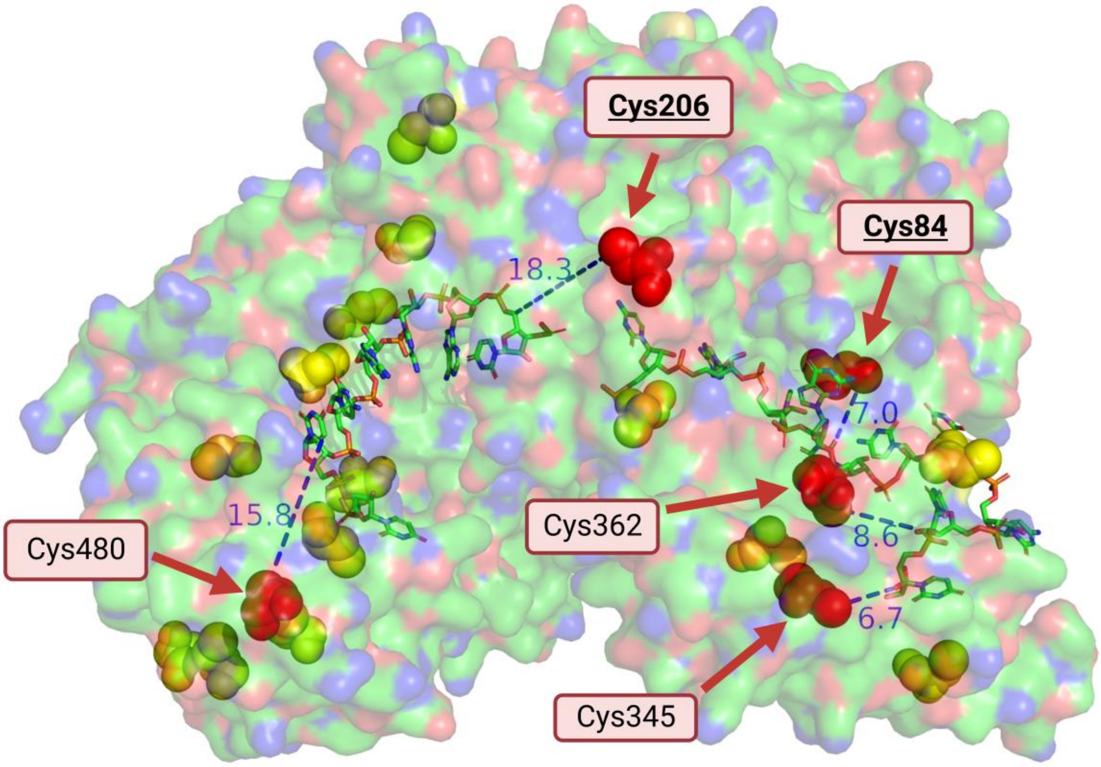

Figure S84. Identified cross-linked cysteines are mapped on hAgo2-siRNA crystal structure (PDB: 4W5N)[10]. Residues are numbered according to amino acid position in our expression construct. Yellow-highlighted cysteines are within 18Å but were not cross-linked. Red-highlighted cysteines were identified in at least one cross-linking replicate. Cysteines with underlined bold label have been identified in all three cross-linking experiments. Distances between sulphur atom and the closest atom in RNA molecule are calculated in ångströms (in blue).

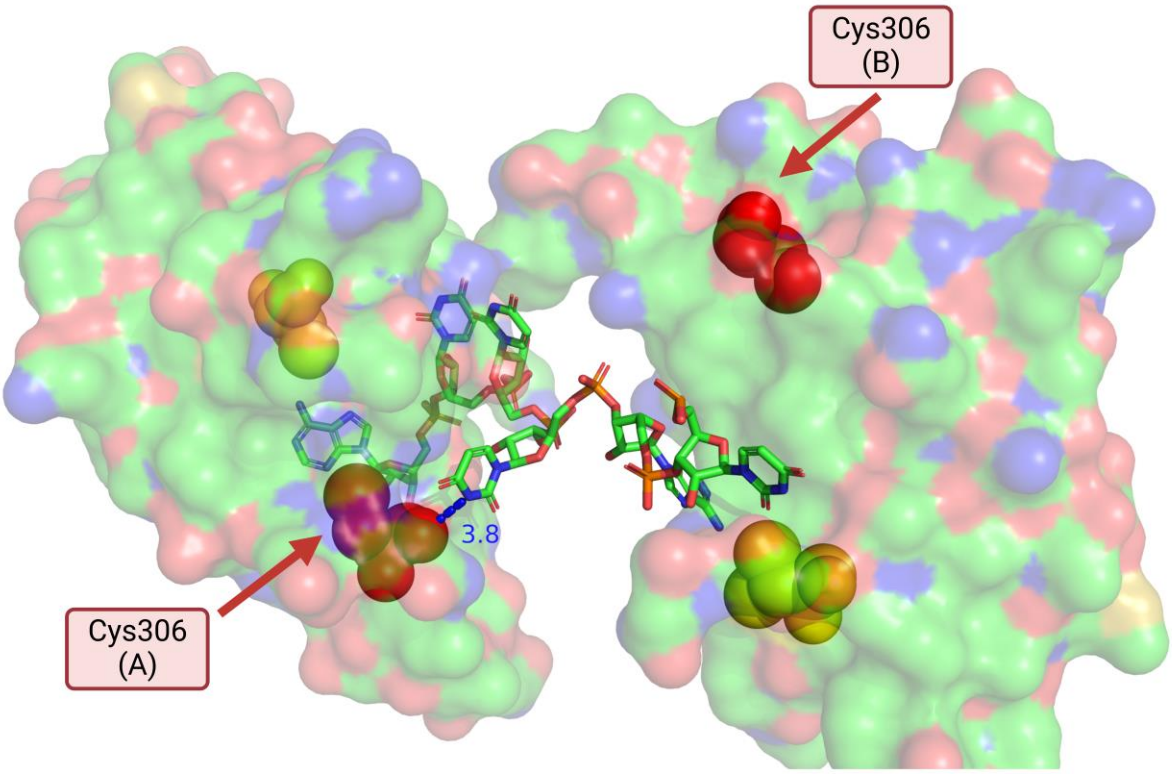

Figure S85. Identified cross-linked cysteines are mapped on HuR (RRM3 domain)-RNA crystal structure (PDB: 6GD3) [11]. Residues are numbered according to amino acid position in our expression construct. Red-highlighted cysteines labelled with magenta box have been identified in the cross-linking experiment. HuR is here present as a dimer and therefore, we are not able to identify, which subunit cross-linked to our probe. The distance is measured for more closer cysteine. Distances between sulphur atom and the closest atom in RNA molecule are calculated in ångströms (in blue).

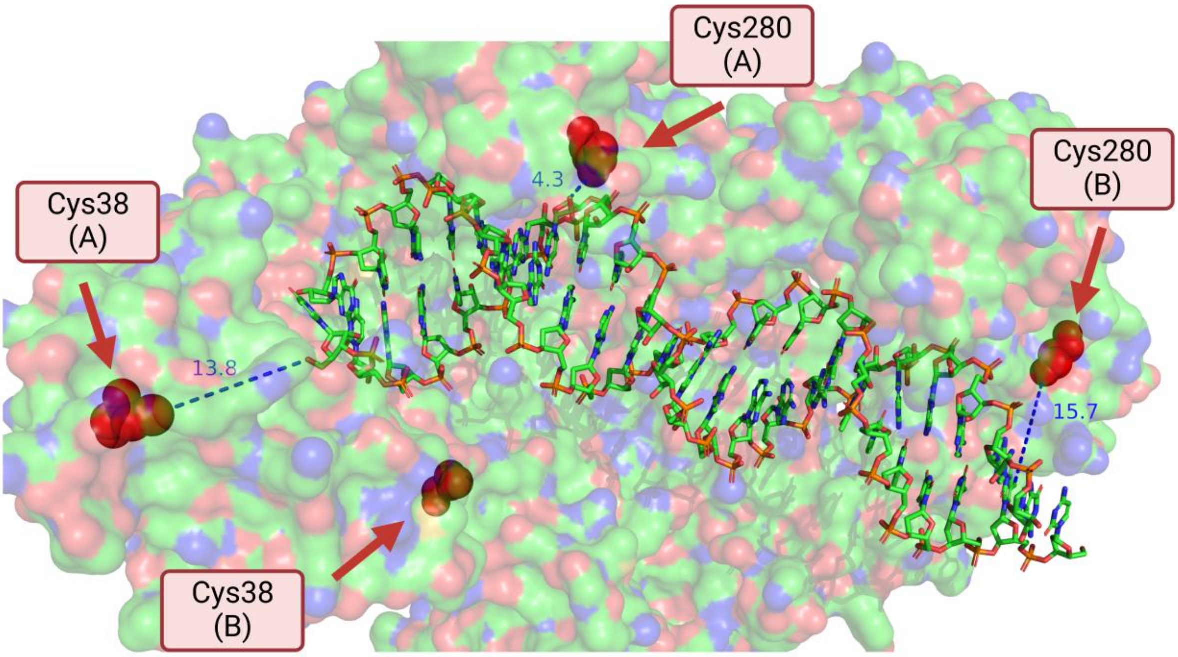

Figure S86. Identified cross-linked cysteines are mapped on HIVRT-RNA:DNA duplex crystal structure (PDB: 1HYS) [12]. Residues are numbered according to amino acid position in our expression construct. Red-highlighted cysteines labelled with magenta box have been identified in the cross-linking experiment. HIV-RT is here present as a dimer and therefore, we are not able to identify, which subunit cross-linked to our probe. The distance is measured for more closer and accessible cysteines. Distances between sulphur atom and the closest atom in RNA molecule are calculated in ångströms (in blue).

5 References

1. A. Bourderioux, P. Nauš, P. Perlíková, R. Pohl, I. Pichová, I. Votruba, P. Džubák, P. Konečný, M. Hajdúch, K. M. Stray, T. Wang, A. S. Ray, J. Y. Feng, G. Birkus, T. Cihlar, M. Hocek, *J Med Chem* **2011**, *54*, 5498–5507.
2. H. Tian, Y. Xu, S. Liu, D. Jin, J. Zhang, L. Duan, W. Tan, *Molecules* **2017**, *22*.
3. M. T. Marty, A. J. Baldwin, E. G. Marklund, G. K. A. Hochberg, J. L. P. Benesch, C. V. Robinson, *Anal. Chem.* **2015**, *87*, 4370–4376.
4. C. Kao, M. Zheng, S. Rüdisser, *RNA* **1999**, *5*, 1268–1272.
5. M. J. Kellner, J. J. Ross, J. Schnabl, M. P. S. Dekens, M. Matl, R. Heinen, I. Grishkovskaya, B. Bauer, J. Stadlmann, L. Menéndez-Arias, A. D. Straw, R. Fritsche-Polanz, M. Traugott, T. Seitz, A. Zoufaly, M. Födinger, C. Wenisch, J. Zuber, A. Pauli, J. Brennecke, *Front Mol Biosci* **2022**, *9*, 270.
6. J. R. Poganik, M. J. C. Long, M. T. Disare, X. Liu, S. H. Chang, T. Hla, Y. Aye, *FASEB Journal* **2019**, *33*, 14636–14652.
7. H. Wang, R. A. Ach, B. O. Curry, *RNA* **2007**, *13*, 151–159.
8. V. Berndt, M. Beckstette, M. Volk, P. Dersch, M. Brönstrup, *Sci Rep* **2019**, *9*, 1–15.
9. J. W. Bae, S. C. Kwon, Y. Na, V. N. Kim, J. S. Kim, *Nat Struct Mol Biol* **2020**, *27*, 678–682.
10. N. T. Schirle, J. Sheu-Gruttadauria, I. J. MacRae, *Science* **2014**, *346*, 608–613.
11. M. Pabis, G. M. Popowicz, R. Stehle, D. Fernández-Ramos, S. Asami, L. Warner, S. M. García-Mauriño, A. Schlundt, M. L. Martínez-Chantar, I. Díaz-Moreno, M. Sattler, *Nucleic Acids Res.* **2019**, *47*, 1011–1029.
12. S. G. Sarafianos, K. Das, C. Tantillo, A. D. Clark, J. Ding, J. M. Whitcomb, P. L. Boyer, S. H. Hughes, E. Arnold, *EMBO J.* **2001**, *20*, 1449–1461.
